# C-Terminal-Modified Oligourea Foldamers as a Result of Terminal Methyl Ester Reactions under Alkaline Conditions

**DOI:** 10.3390/ijms24076806

**Published:** 2023-04-06

**Authors:** Katarzyna Kedzia, Lukasz Dobrzycki, Marcin Wilczek, Karolina Pulka-Ziach

**Affiliations:** 1 Faculty of Chemistry, University of Warsaw, Pasteura 1, 02-093 Warsaw, Poland; km.kedzia@chem.uw.edu.pl (K.K.); wilczek@chem.uw.edu.pl (M.W.); 2 Laboratory of Advanced Crystal Engineering, Faculty of Chemistry, University of Warsaw, Żwirki i Wigury 101, 02-089 Warsaw, Poland; dobrzyc@chem.uw.edu.pl

**Keywords:** foldamers, oligourea amino acid hybrids, hydantoin ring stability

## Abstract

Hybrids of short oligourea foldamers with residues of α, β and γ-amino acids esters at the C-terminus were obtained and subjected to a reaction with LiOH. There are two possible transformations under such conditions, one of which is ester hydrolysis and the formation of a carboxylic group and the other is the cyclization reaction after abstraction of a proton from urea by a base. We have investigated this reaction with difference C-terminal residue structures, as well as under different work-up conditions, especially for oligourea hybrids with α-amino acid esters. For these compounds, an oligourea–hydantoin combination is the product of cyclization. The stability of the hydantoin ring under alkaline conditions has been alsotested. Furthermore, this work reports data related to the structure of C-terminal-modified oligourea foldamers in solution and, for one compound, in the solid state. Helical folding is preserved both for cyclized and linear modifications, with oligourea–acid hybrids appearing to be more conformationally stable, as they are stabilized by an additional intramolecular hydrogen bond in comparison to cyclic derivatives.

## 1. Introduction

In recent years, oligourea foldamers with the general formula [NHCH(R)CH_2_NHCO]_n_ have gained much attention as structural and functional mimetics of peptides [1,2,3,4,5,6,7,8]. It has been shown that oligoureas can mimic the helical secondary structure of peptides. Oligourea foldamers fold into 2.5 helices, stabilized by a network of 3-centered hydrogen bonds, forming 12- and 14-membered pseudorings [2,4]. The secondary structure is independent of the sequence, and even short oligomers tend to form stable spatial structures in solution and in the solid state [2]. It has been shown that water-soluble oligourea foldamers with carefully designed sequences are also able to form higher-ordered structures such as bundles or channels [9,10,11,12]. Moreover, it is possible to encapsulate guest molecules inside the bundle [13,14]. It has also been demonstrated that oligoureas can act as electron transport mediators, similar to peptides and proteins [15,16,17]. The stability of the helical conformation of these oligomers has enabled studies which have elucidated the mechanism of electron transfer to be length dependent, changing from tunneling to hopping with the length of the molecule and thereby the thickness of the monolayer [15,16].

Over the years the (oligo)urea backbone has undergone various modifications and the impact of these changes on the structural properties of the new analogs has been investigated. This has led to the conclusion that the 2.5-helical structures of oligoureas are robust and it is possible to substitute the urea residue with γ-amino acid [6,18], carbamate [18,19], thiourea [20], guanidinium [21] and also N-methylated [22] or urea residues with noncanonical substitution patterns [23] without the loss of the structural integrity of the new molecule. It has also been shown that urea fragments as short as three residues can induce a helical conformation when fused to a peptide too short to fold into a stable secondary structure [24,25]. Such α-peptide/urea chimeras adopt a continuous and well-defined helical structure spanning the entire sequence stabilized by a H bond network, regardless of the position (e.g., N-terminal or C-terminal) of the urea segment on the peptide fragment [24]. 

**Figure 1:**
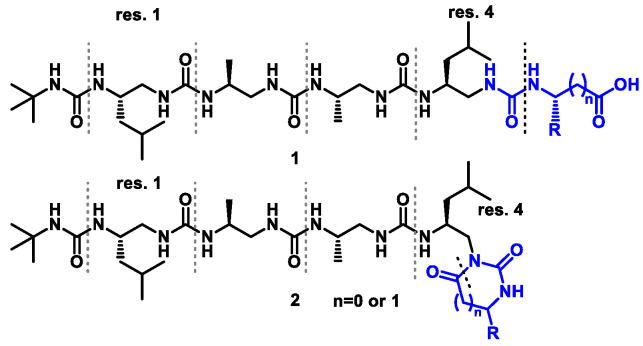
C-terminal-modified oligourea foldamers. The numbering of residues is carried out from the N- to the C-terminus, in contrast to what is usually used in the literature.

The aim of the work reported here was to obtain oligourea foldamers containing carboxyl groups at the C-terminus under alkaline conditions. Similar connections of urea fragments with α-amino acid residues were reported by Liskamp and co-workers [26]. They developed a solid phase synthetic methodology allowing them to obtain the desired compounds [26]. We performed our syntheses in solution and decided to expand the types of C-terminal residues to include β- and γ-amino acids. It turned out that in addition to the expected product **1**, compound **2** (Figure 1), as the result of the cyclization of the last residue, was also formed. The type of the resultant product (**1** or **2**) depended on the structure of the C-terminal residue, as well as on the work-up conditions. In this paper we also report the influence of C-terminal modifications on the secondary structure of the obtained compounds. 

## 2. Results

### 2.1. Synthesis of Oligourea Methyl Esters and Their Transformations under Alkaline Conditions and Stability Studies of Oligourea-Hydantoin Derivatives

**Scheme 1:**
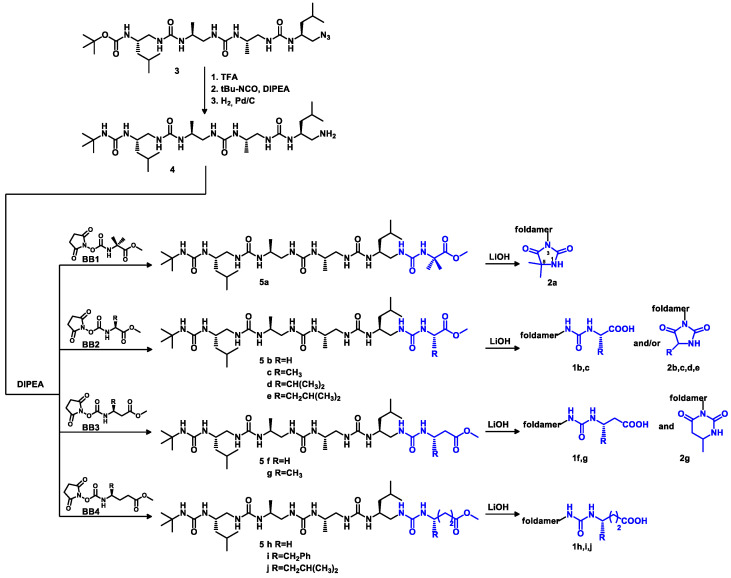
Synthesis of methyl esters of oligourea foldamers and subsequent reaction under alkaline conditions.

Short chain oligoureas were obtained following well-established methods in solution, utilizing Boc-protected activated succinimidyl carbamates [4,27]. At the C-terminus, the methyl esters of varied amino acids in the form of succinimidyl carbamates were coupled (see the Supporting Information, Figure S1) to form a urea bond. To facilitate the coupling of different amino acids residues, oligourea synthesis was initiated by Boc-protected 1-isobutyl-2-azidoethylamine and then carried out by sequential linkage of individual succinimidyl carbamates from the so-called C-terminus to the N-teminus to obtain the desired tetramer, as it has previously been reported [15]. To incorporate the C-terminal residues, the azide function in compound **3** (Scheme 1) was reduced to an amine group with hydrogen in the presence of Pd/C, and then succinimidyl carbamate derivatives of methyl esters of α-, β- and γ-amino acids (**BB1**–**BB4**) were used in the coupling reaction. Compounds of type **5** were reacted at RT with excess (10 eq.) LiOH in a 4:1 mixture of MeOH and H_2_O (Scheme 1). The reaction progress was monitored by RP-HPLC (see the Supporting Information, Figures S49–S60). Under such conditions, two types of products were possible: a linear acid as a result of the hydrolysis reaction and/or a cyclic product as a result of proton abstraction of the urea group and intramolecular 5- or 6-membered ring formation (Figure 1, see also the Supporting Information, Figures S2 and S3). In our case, the result of the reaction strongly depended on the structure of the last C-terminal residue of the foldamers as well as on the work-up conditions. For derivatives of oligoureas containing α-amino acids, the disappearance of the substrate was observed after 2h, and for the Gly residue at the C-terminus (**5b**), both products **1** and **2** containing a hydantoin ring [28,29,30] were formed in the ratio 3:7. For Aib, Ala, Val and Leu (**5a,c,d,e**) at the C-terminus, products of type **2** were formed in more than 95% yield in the crude reaction mixture. In all cases we observed the epimerization of the stereogenic center of the amino acid residue and so a mixture of diastereomeric products was formed. According to the NMR spectra, the ratio of diastereomers was defined as approximately 1:1 (see the Supporting Information, Figure S47). For Ala derivative **5c**, in the crude product after the work-up, the amount of acid **1c** increased to 25% (see the explanation below). For foldamers with Aib, Val and Leu at the C-terminus, acid **1** was not observed after the work-up. 

It is known from the literature that ureido esters form hydantoin analogs under alkaline conditions, but most of the examples refer to solid phase synthetic procedures [26,31,32]. Liskamp and co-workers [26] observed that resin-bound oligourea was cleaved off the Tentagel resin as substituted hydantoin under basic conditions in the presence of KCN in MeOH. Subsequently, various hydroxides were tested by them on the model compound and it was discovered that the highest amount of hydantoin was formed with NaOH (45%), whereas TBAOH favored the acid form (>98%) of the peptidomimetic. 

Next, we decided to investigate the outcome of the reaction of β- and γ-amino acid residues at the C-terminus. The linear compound **1f** was formed for unbranched βhGly, whereas for β^3^hAla, a mixture (around 1:1, see the Supporting Information, Figure S56) of two products (**1g** and **2g**) was obtained. For γ-amino acid derivatives, only compounds **1h–j** were formed as products of the simple hydrolysis of methyl esters and no cyclic products were observed. 

We speculate that the outcome (cyclisation versus hydrolysis) of the reaction of oligourea methyl esters of type **5** with LiOH and the dependence on the structure of the last amino acid residue may be caused, among other reasons, by steric factors. The reaction occurs with oligomers long enough to fold into 2.5 helices, as confirmed by 2D NMR ROESY spectra [2]. All urea groups adopt a *trans,trans* conformation (see the Supporting Information, Figure S48), which is necessary for helical folding and formation of the intramolecular hydrogen bonding network. For terminal amino acid residues with short distances between the last urea group at the C-terminus and the methyl ester moiety, as present in α-amino acids, the attack of the OH^−^ anion on the carbonyl carbon may be hampered because of the helix. On the other hand, cyclization is favored by the proximity of the reacting groups. For Gly derivatives, the unbranched chain seems to be flexible enough to give both products. The elongation of the terminal amino acid residues by additional methylene groups, as in β- and further γ-derivatives, causes more lability of the carbon chain and extends the distance of the ester group, which facilitates alkaline hydrolysis and the formation of products of type **1**. 

**Figure 2:**
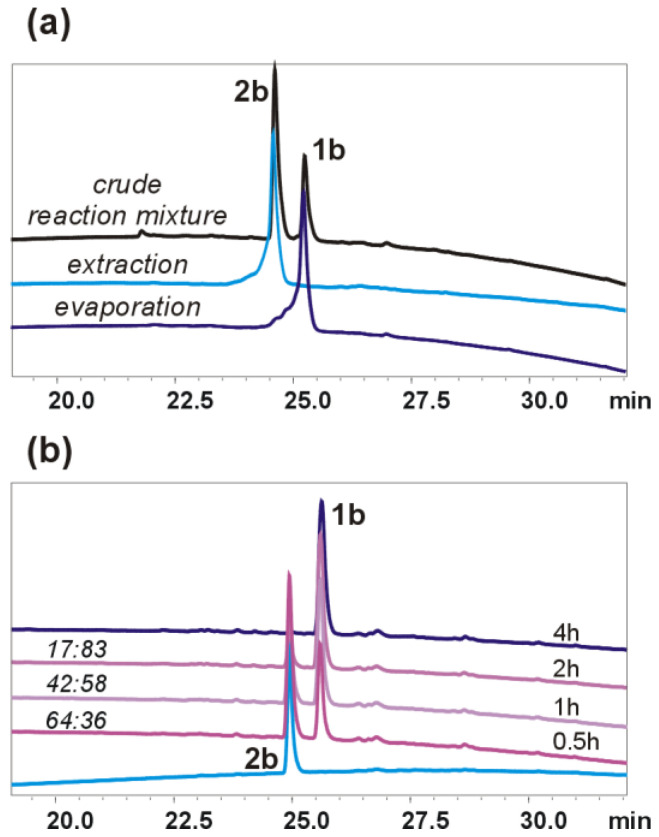
(**a**) Dependence of the products of the reaction on the work-up method. (**b**) Stability studies of compound **2b** at 45 °C under alkaline conditions.

The reaction of oligourea **5b** was then studied in more detail. We have discovered that the ratio of cyclic to linear products before and after isolation strongly depended on the work-up method used. In the crude reaction mixture after 2 h, the ratio of **1b** and **2b** determined by HPLC was 3:7. The crude was then highly diluted with water and DCM and after extraction, the compound **2b** was obtained from the organic phase, whereas **1b** was isolated from the aqueous phase after acidification. On the other hand, when the crude reaction mixture was evaporated to dryness and then re-dissolved in a mixture of DCM and water, acidified with 1M HCl and extracted, the only isolated product was compound **1b** (Figure 2a). It was evident that during evaporation, the hydantoin ring in **2b** underwent hydrolysis and oligourea **1b** with a glycine residue at the C-terminus was formed [29,33,34]. Interestingly, the foldamer with a cyclic residue at the C-terminus is slightly more polar than the foldamer with a carboxylic moiety (Figure 2a). 

To investigate the influence of the temperature on the conversion of the hydantoin residue into a carboxylic acid moiety, we took compound **2b** and performed the reaction at 45 °C (Figure 2b). The concentration of compound **2b** was twice as high, but the excess of LiOH relative to the compound was the same. The reaction was stirred in a 1:1 MeOH:H_2_O mixture, i.e., with a higher amount of water. This was to mimic the conditions during the evaporation and reduction of organic solvent in favor of water. By HPLC, we observed a gradual disappearance of hydantion derivative **2b** and its conversion into oligourea acid **1b** (Figure 2b). After 4 h of stirring at 45 °C, the conversion was complete. The reaction using the same concentration and mixture of solvents was also performed at RT (23 °C). After 2 h of stirring, compound **2b** still dominated (64% by HPLC) and even after 24 h it was still observed in the reaction mixture (5%, see the Supporting Information, Figure S95a). To test the influence of the substituent at position 5 of the hydantoin ring and its size, we performed the same reaction on diastereomers of **2e** (see the Supporting Information, Figure S95b,c). After 2 h at 45 °C, we observed the formation of only 9% of the acidic form, whereas at RT, the hydantoin ring was stable and did not undergo hydrolysis. These observations confirmed that the isolated products after the reaction with LiOH strongly depended on the work-up conditions for oligoureas with unsubstituted residues (such as Gly) or those with small side chains (such as Ala). 

### 2.2. Structural Studies of Hydantoin and Carboxylic Acid Derivatives of Oligoureas

The structural consequences of the presence of a hydantoin ring or carboxylic group at the C-terminus of short oligourea chains were investigated in solution by NMR and ECD. Additionally, for compound **2a**, we obtained a monocrystal suitable for X-ray analysis. 

NMR spectra of all compounds were recorded at a concentration of ca. 3 mM in 10% DMSO-d_6_ in CD_3_CN. Proton resonances were assigned by using a combination of COSY, TOCSY, ROESY and heteronuclear HSQC 2D experiments (see the Supporting Information, Tables S1 and S2). To compare the propensity of folding into a 2.5 helix for C-terminal-modified oligoureas, compounds **1f** and **2a** were chosen.

**Figure 3:**
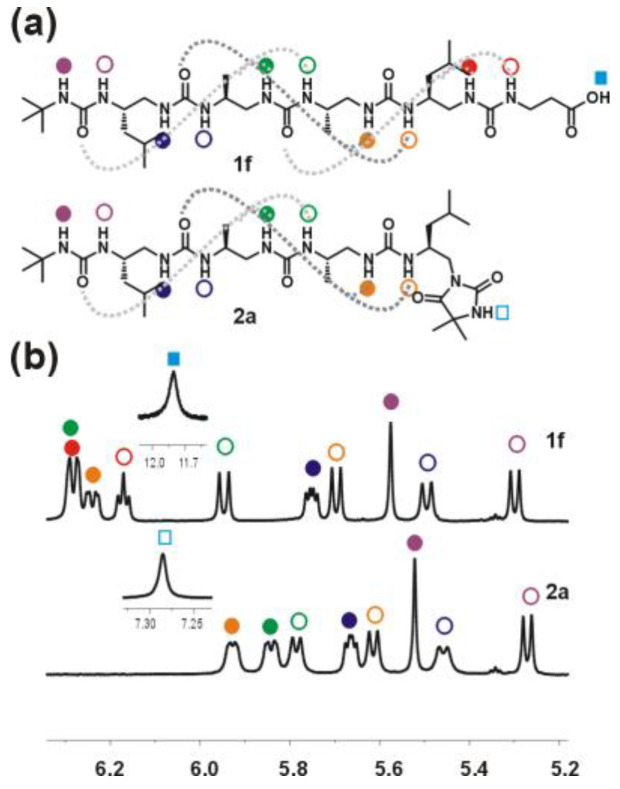
(**a**) Structures of compounds **1f** and **2a**, with grey curves showing the scheme of intramolecular hydrogen bonds of the 2.5 helix. (**b**) Parts of the ^1^H NMR spectra (500 MHz) of compounds **1f** and **2a** in 10% DMSO-d_6_ in CD_3_CN (3 mM) showing the NH/N’H region.

As shown in Figure 3b, the chemical shifts of urea protons are visible between 5.2 and 6.3 ppm for **1f**, whereas for **2a**, the highest chemical shift is less than 6.0 ppm. Only slight changes in chemical shifts (0.03–0.09 ppm) were observed for urea protons at the N-terminus not involved in intramolecular hydrogen bonds. The highest downfield shifts (0.16–0.44 ppm) for oligourea acids in comparison to oligourea hydantoins were observed for the middle urea group N’H(Ala2u) and NH(Ala3u) (marked in green in Figure 3b, (u) means urea residue). This is because the urea moiety in oligourea acid is involved in helix stabilization both as a donor and acceptor of intramolecular hydrogen bonds (Figure 3a). Based on ROESY spectra, it was confirmed that all urea groups of the oligourea hydantoin and oligourea acid are in *trans,trans* conformations (see the Supporting Information, Figure S48), known to be necessary for helical folding of the backbone. Additionally, strong ROE connectivity between CH_3_(tBu) and both N’H(Ala2u) and NH(Ala3u) was observed, further supporting the formation of a 2.5 helix [22].

It is well known that the differences in the chemical shifts (Δδ) of CH_2_ protons of the main chain are indicative of 2.5 helix formation [1,2,3]. We compared Δδ values for C-terminal-modified oligoureas and discovered that for oligourea hydantoin **2a**, the diastereotopicity values were noticeably lower than for oligourea acid **1f** (Table 1). This is expected to be due to the difference in the number of possible intramolecular hydrogen bonds (two for hydantoin derivatives and three for acid derivatives) and it is in agreement with the literature reports on similar length compounds [2]. 

**Figure 4:**
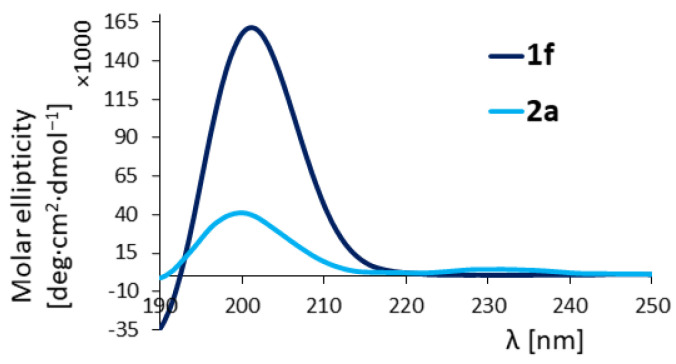
Circular dichroism spectra of compounds **1f** (oligourea acid) and **2a** (oligourea hydantoin) in TFE.

To further investigate the folding propensity of oligoureas **2a** and **1f** in solution, ECD spectra were measured (Figure 4, see also Supporting Information, Figure S94) at a concentration of 0.2 mM in 2,2,2-trifluoroethanol (TFE). Both types of oligourea derivatives exhibited the typical CD signature of oligoureas [2], with positive bands at λ = 200 and 201 nm for hydantoin and acid derivatives, respectively. Interestingly, the intensity of the band was significantly lower for **2a** than for **1f**, strongly suggesting that the secondary structure of oligourea hydantoins is less stabilized and therefore less stable in solution than the oligourea acids. 

**Figure 5:**
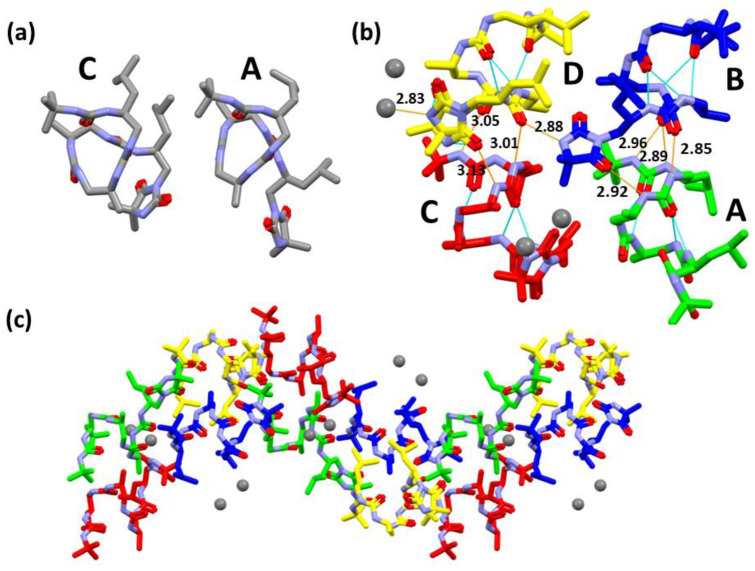
Crystal structure of compound **2a**: (**a**) two (A and C) of four independent molecules in the asymmetric unit, showing different positions of the hydantoin ring, (**b**) the length of intermolecular hydrogen bonds (orange), green—A, blue—B, red—C, yellow—D and (**c**) packing of oligourea hydantoin **2a**.

For compound **2a**, we were able to obtain a crystal structure, providing definite confirmation of the secondary structure of compound **2a** in the solid state. The crystal structure was solved in the P2_1_ space group. There are four independent molecules (A–D) and 2.5 water molecules present in the asymmetric unit (Figure 5, see the Supporting Information, Table S3 and Figures S96–S99). 

The oligourea fragment is seen to be stabilized by two intramolecular hydrogen bonds, forming a helical turn, while the individual molecules of the asymmetric unit (A–D) differ in the position of the hydantoin ring relative to the rest of the molecule, as shown by the N-C-C-N(hyd) torsion angles of 41° (C) and 43° (D), when the hydantoin ring is twisted towards the helix and −159.3° (A) and −163.6° (B), when the ring is flipped outside of the helix. For other residues, the N-C-C-N’ torsion angles are between 47° and 57°(see the Supporting Information, Table S4), typical values for canonical oligourea 2.5 helices [19]. Molecules A and B, as well as C and D, are hydrogen bonded by head to tail intermolecular interactions (Figure 5b). These intermolecular hydrogen bonds are formed between O (Ala3u) and NH (tBu)/NH (Leu1u) and also O (Leu4u) and N’H (Leu1u). The intermolecular O-N distances for molecules A and B are around 2.9 Å, whereas for molecules C and D, this distance is slightly longer at around 3 Å. Additionally, the hydantoin ring is involved in an intermolecular hydrogen bonding network between A and B as well as C and D molecules, and CO (4) (for numeration of the hydantoin ring, see Scheme 1) is bonded to NH (Ala2u). Furthermore, NH (1) of the hydantoin ring in A and B is linked to O (Leu4u) of the C and D molecules. The same hydrogen in C and D is bonded to the oxygen of water molecules. All O-N distances described above are around 3 Å. Thanks to the network of intermolecular interactions, the crystal lattice of molecules A and D as well as B and C form superhelices (Figure 5c). 

The results obtained from the crystal structure, together with the solution studies, reveal that the hydantoin ring is somewhat labile and may adopt a number of different orientations relative to the folded part of the molecule. 

## 3. Materials and Methods

### 3.1. General Consideration

Solvents and reagents are commercially available. ^1^H and ^13^C NMR spectra were recorded on a Bruker Avance 300 or 500 MHz for ^1^H and 75 MHz for ^13^C. Two-dimensional NMR spectra (COSY, TOCSY, HSQC and ROESY) were recorded on a Bruker Avance III HD 500 MHz. The chemical shifts were reported in ppm (δ) and coupling constants (*J*) were given in Hz. To indicate multiplicity, the abbreviations singlet (s), broad singlet (bs), doublet (d), triplet (t), quartet (q), doublet of doublets (dd) and multiplet (m) were used. Splitting patterns which were not easy to interpret were labelled as multiplets (m). The HRMS data were obtained on a QExactive mass spectrometer with electrospray ionization. Column chromatography was carried out on silica gel (70–230 mesh). Analytical RP-HPLC analyses were performed using a Jupiter 4u Proteo 90Å column (4.6 × 250 mm) at a flow rate of 1 mL × min^−1^. The mobile phase was composed of 0.1% (*v*/*v*) TFA/H_2_O (phase A) and 0.1% (*v*/*v*) TFA−CH_3_CN (phase B). The detection was performed at 200 nm and the method used was 10% of B for 5 min and a 10–97% gradient of B in 30 min. Activated (S)-succinimidyl-{2-{[(tert-butoxy)carbonyl]amino}-2-X-ethyl}carbamate monomers for the introduction of urea bonds were prepared from corresponding N-Boc-protected ethylene diamine derivatives using recently reported procedures [27,35]. Tetramer **3** was synthesized as previously described [15]. Known compounds synthesized by literature methods were confirmed by comparing reported characterization data.

### 3.2. Synthetic Details

#### 3.2.1. General Procedure for the Synthesis of Building Blocks **BB1**–**BB4**

All methyl ester carbamate building blocks (**BB1**, **BB2b**, **BB2c**, **BB2d**, **BB2e**, **BB3f**, **BB3g** and **BB4h**) were obtained from appropriate amino acids using the same two step procedure.

Step I: Thionylchloride (2 eq.) was slowly added to a cooled (−78 °C) suspension of amino acid (1 eq.) in CH_3_OH (20 mL/g) under an inert atmosphere. Then, the reaction mixture was warmed up to ambient temperature and stirred at this temperature overnight. Following this, the solvent was removed under reduced pressure and the solid residue was filtered and washed with diethyl ether. The product was taken to the next step without further purification.

Step II: The product (1 eq.,) from the previous step was dissolved in dry DCM (20 mL/g) at 0 °C, and DIPEA (3 eq.) was added. After stirring for 10 min, the solution was added dropwise into a flask containing solid N,N′-disuccinimidyl carbonate (DSC, 1.2 eq.). The reaction was stirred for 3 h under Ar at RT. Following this, the reaction mixture was washed with 1M KHSO_4_ (3×) and brine (1×). In some cases, the products remained in the aqueous layer, so this layer was extracted with DCM until all the desired compound was in the organic layer. Combined organic layers were dried over Na_2_SO_4_. After evaporation under reduced pressure, the crude product was obtained as a colorless/yellowish oil. It was purified by dissolving it in small amount of DCM, followed by precipitation with diethyl ether and/or petroleum ether. The pure product was obtained as a white solid (except **BB4h**, which was a yellowish oil used without further purification). Copies of ^1^H and ^13^C NMR spectra, as well as HRMS spectra of building blocks are given in the Supporting Information, Figures S4–S23 and Figures S61–S70.

**BB1**: reagents in step I: 2-aminoisobutyric acid (2 g, 19.4 mmol), thionyl chloride (2.8 mL, 38.8 mmol) and methanol (40 mL). Reagents in step II: DSC (5.97 g, 23.3 mmol), DIPEA (10,1 mL, 58.2 mmol) and DCM (60 mL).

White solid, yield (after 2 steps): 56% (2.8g); ^1^H NMR (300 MHz, CDCl_3_) δ 6.18 (s, 1H), 3.82 (s, 3H), 2.84 (s, 4H), 1.64 (s, 6H); ^13^C NMR (75 MHz, CDCl_3_) δ 173.74, 169.45, 149.21, 57.25, 52.71, 25.08, 23.98; HRMS ESI(+) calcd. for C_10_H_15_N_2_O_6_ 259.09246 [M + H]+, found 259.09251.

**BB2b**: reagents in step I: Gly (293 mg, 3.9 mmol), thionyl chloride (0.57 mL, 7.8 mmol) and methanol (6 mL). Reagents in step II: DSC (1.17 g, 4.68 mmol), DIPEA (2.04 mL, 11.7 mmol) and DCM (6 mL).

White solid, yield (after 2 steps): 51% (0.46g); ^1^H NMR (300 MHz, CDCl_3_) δ 6.11 (s, 1H), 4.07 (d, *J* = 5.3 Hz, 2H), 3.81 (s, 3H), 2.85 (s, 4H); ^13^C NMR (75 MHz, CDCl_3_) δ 169.66, 169.08, 151.39, 52.72, 43.10, 25.47; HRMS ESI(+) calcd. for C_8_H_11_N_2_O_6_ 231.06116 [M + H]+, found 231.06131.

**BB2c**: reagents in step I: L-Ala (1 g, 11.2 mmol), thionyl chloride (1.62 mL, 22.45 mmol) and methanol (20 mL). Reagents in step II: DSC (3.44 g, 13.44 mmol), DIPEA (7.0 mL, 40.32 mmol) and DCM (20 mL).

White solid, yield (after 2 steps): 51% (1.4 g); ^1^H NMR (300 MHz, CDCl_3_) δ 6.02 (d, *J* = 7.2 Hz, 1H), 4.40 (p, *J* = 7.2 Hz, 1H), 3.81 (s, 3H), 2.84 (s, 4H), 1.52 (d, *J* = 7.1 Hz, 3H); ^13^C NMR (75 MHz, CDCl_3_) δ 171.79, 169.63, 150.48, 52.35, 50.09, 25.08, 17.70; HRMS ESI(+) calcd. for C_9_H_13_N_2_O_6_ 245.07681 [M + H]+, found 245.07693.

**BB2d:** reagents in step I: L-Val (1 g, 8.54 mmol), thionyl chloride (1.24 mL, 17.08 mmol) and methanol (20 mL). Reagents in step II: DSC (2.63 g, 10.25 mmol), DIPEA (4.46 mL, 25.62 mmol) and DCM (20 mL).

White solid, yield (after 2 steps): 59% (1.38 g); ^1^H NMR (300 MHz, CDCl_3_) ^1^H NMR (300 MHz, CDCl_3_) δ 5.93 (d, *J* = 8.8 Hz, 1H), 4.29 (dd, *J* = 8.8, 4.7 Hz, 1H), 3.80 (s, 3H), 2.84 (s, 4H), 2.23 (m, 1H), 1.01 (d, *J* = 6.9 Hz, 1H), 0.96 (d, *J* = 6.9 Hz, 1H); ^13^C NMR (75 MHz, CDCl_3_) δ 171.17, 169.57, 151.34, 60.00, 52.54, 31.77, 25.47, 18.77, 17.45; HRMS ESI(+) calcd. for C_11_H_17_N_2_O_6_ 273.10811 [M + H]+, found 273.10806.

**BB2e**: reagents in step I: L-Leu (2 g, 15.0 mmol), thionyl chloride (2.18 mL, 30 mmol) and methanol (20 mL). Reagents in step II: DSC (3.9 g, 15.24 mmol), DIPEA (6.6 mL, 38.1 mmol) and DCM (20 mL).

White solid, yield (after 2 steps): 66% (2.39 g); ^1^H NMR (300 MHz, CDCl_3_) δ 5.79 (d, *J* = 8.5 Hz, 1H), 4.41 (td, *J* = 8.4, 5.1 Hz, 1H), 3.80 (s, 3H), 2.84 (s, 4H), 1.82–1.57 (m, 3H), 0.98 (dd, *J* = 6.2, 3.7 Hz, 6H); ^13^C NMR (75 MHz, CDCl_3_) δ 171.82, 169.58, 150.84, 52.98, 52.17, 41.15, 25.07, 24.22, 22.30, 21.38; HRMS ESI(+) calcd. for C_12_H_19_N_2_O_6_ 287.12376 [M + H]+, found 287.12383.

**BB3f**: reagents in step I: βhGly (2 g, 22.7 mmol), thionylchloride (3.3 mL, 45.3 mmol) and methanol (40 mL). Reagents in step II: DSC (6.98 mg, 27.24 mmol), DIPEA (11.86 mL, 68.1 mmol) and DCM (40 mL).

White solid, yield (after 2 steps): 62% (3.64 g); ^1^H NMR (300 MHz, CDCl_3_) δ 5.99 (s, 1H), 3.74 (s, 3H), 3.55 (q, *J* = 6.1 Hz, 2H), 2.84 (s, 4H), 2.63 (t, *J* = 6.0 Hz, 2H); ^13^C NMR (75 MHz, CDCl_3_) δ 172.46, 169.83, 151.41, 52.05, 37.42, 33.58, 25.47; HRMS ESI(+) calcd. for C_9_H_13_N_2_O_6_ 245.07681 [M + H]+, found 245.07679.

**BB3g**: reagents in step I: (S)-3-Aminobutanoic acid (300 mg, 2.9 mmol), thionyl chloride (0.42 mL, 5.8 mmol) and methanol (7 mL). Reagents in step II: DSC (891 mg, 3.48 mmol), DIPEA (1.5 mL, 8.7 mmol) and DCM (10 mL).

White solid, yield: 53% (0.4 g); ^1^H NMR (300 MHz, CDCl_3_) δ 5.93 (d, *J* = 8.8 Hz, 1H), 4.13 (m, 1H), 3.74 (s, 3H), 2.84 (s, 4H), 2.64 (m, 2H), 1.35 (d, *J* = 6.8 Hz, 3H); ^13^C NMR (75 MHz, CDCl_3_) δ 171.09, 169.75, 150.24, 51.49, 44.97, 39.50, 25.08, 19.59; HRMS ESI(+) calcd. for C_10_H_15_N_2_O_6_ 259.09246 [M + H]+, found 259.09259.

**BB4h** reagents in step I: γ-aminobutyric acid (1 g, 9.7 mmol), thionyl chloride (1.40 mL, 19.4 mmol) and methanol (20 mL). Reagents in step II: DSC (2.82 g, 11.64 mmol), DIPEA (5.0 mL, 29.1 mmol) and DCM (20 mL).

Yellowish oil, yield (after 2 steps): 64% (1.69 g); ^1^H NMR (300 MHz, CDCl_3_) δ 5.94 (s, 1H), 3.70 (s, 3H), 3.32 (q, *J* = 6.8 Hz, 2H), 2.83 (s, 4H), 2.42 (t, *J* = 7.2 Hz, 2H), 1.91 (p, *J* = 7.0 Hz, 2H); ^13^C NMR (75 MHz, CDCl_3_) δ 173.63, 170.05, 151.56, 51.85, 41.41, 31.11, 25.48, 24.46; HRMS ESI(+) calcd. for C_10_H_15_N_2_O_6_ 259.09246 [M + H]+, found 259.09249.

Both methyl ester carbamate building blocks, **BB4i** and **BB4j**, were obtained starting from Boc-protected γ-amino acids. Boc deprotection procedure: protected γ-AA was treated with TFA (6 mL/g) and stirred at 0 °C for 1 h under Ar. When the deprotection reaction was complete, TFA was removed and the residue was co-evaporated with cyclohexane (3×) and diethyl ether (1×) and then reacted with thionyl chloride. 

**BB4i**: reagents in step I: Boc-γ-Phe (250 mg, 0.85 mmol), TFA (1.5 mL), thionyl chloride (0.12 mL, 1.7 mmol) and methanol (5 mL). Reagents in step II: DSC (220 mg, 0.86 mmol), DIPEA (0.38 mL, 2.16 mmol) and DCM (5 mL).

White solid, yield (after 3 steps): 54% (0.16 g); ^1^H NMR (300 MHz, CDCl_3_) δ 7.42–7.18 (m, 5H), 5.31 (d, *J* = 7.0 Hz, 1H), 3.92 (m, 1H), 3.71 (s, 3H), 3.03–2.85 (m, 2H), 2.84 (s, 4H), 2.45 (t, *J* = 7.2 Hz, 2H), 2.04–1.72 (m, 2H); ^13^C NMR (75 MHz, CDCl_3_) δ 173.54, 169.54, 150.78, 136.50, 129.09, 128.27, 126.40, 53.31, 51.49, 40.48, 30.23, 28.10, 25.08; HRMS ESI(+) calcd. for C_17_H_21_N_2_O_6_ 349.13941 [M + H]+, found 349.13942.

**BB4j**: reagents in step I: Boc-γ-Leu (350 mg, 1.35 mmol), TFA (2 mL), thionylchloride (0.19 mL, 2.7 mmol) and methanol (7 mL). Reagents in step II: DSC (295 mg, 1.15 mmol), DIPEA (0.5 mL, 2.88 mmol) and DCM (7 mL).

Yellowish oil, yield (after 3 steps): 70% (295 mg); ^1^H NMR (300 MHz, CDCl_3_) δ 5.12 (d, *J* = 9.3 Hz, 1H), 3.81–3.73 (m, 1H), 3.72 (s, 3H), 2.83 (s, 4H), 2.45 (t, *J* = 7.4 Hz, 2H), 2.01–1.89 (m, 1H), 1.81–1.68 (m, 2H), 1.40–1.22 (m, 2H), 0.95 (dd, *J* = 6.6, 1.7 Hz, 6H); ^13^C NMR (75 MHz, CDCl_3_) δ 173.56, 169.79, 150.99, 51.37, 50.44, 43.94, 30.04, 29.93, 25.07, 24.32, 22.44, 21.71; HRMS ESI(+) calcd. for C_14_H_23_N_2_O_6_ 315.15506 [M + H]+, found 315.15493.

#### 3.2.2. Synthesis of Amine **4**

To a solution of compound **3** (100 mg, 0.17 mmol) in CH_3_OH (15 mL), palladium on charcoal (10% *w*/*w*, 10 mg) in 2 mL of CH_3_OH was added. The reaction mixture was stirred under a 1 bar H_2_ atmosphere (balloon) for 5h. Then, it was filtered through Celite, concentrated under vacuo and co-evaporated with diethyl ether (2×). Product **4** was used without further purification. RP-HPLC: *t*_R_ = 24.48 min. 

#### 3.2.3. Synthesis of Oligourea Esters **5**

Amine **4** (1 eq.) was dissolved in CH_3_CN (40 mL/g) and DMF (10 mL/g) under Ar, and DIPEA (3 eq.) was added at basic pH. The mixture was left for 15 min, followed by a dropwise addition of appropriate building block **BB1–4** (1.2 eq.) in CH_3_CN solution (7 mL/g). The reaction was left overnight at ambient temperature. When the reaction was complete, solvents were removed under vacuum and co-evaporated with toluene (3×) to remove DMF. The residue was redissolved in EtOAc, washed with 1M KHSO_4_ (4×), saturated NaHCO_3_ (1×), brine (1×) and water (1×) and then evaporated. The crude product was purified by silica gel chromatography (CH_2_Cl_2_:CH_3_OH). The pure product was obtained as a white solid. Copies of ^1^H NMR spectra, as well as HRMS spectra of oligourea esters are given in the Supporting Information, Figures S24–S33 and Figures S71–S80.

Oligourea ester **5a**: Substrates: amine **4** (80 mg, 0.14 mmol), **BB1** (44 mg, 0.17 mmol) and DIPEA (73 μL, 0.42 mmol). Purification: silica gel chromatography (CH_2_Cl_2_:MeOH (*v*/*v*) 95:5); yield: 97% (95mg); ^1^H NMR (300 MHz, 10% DMSO-d6 in CD_3_CN) δ: 6.60 (s, 1H), 6.32 (m, 2H), 6.20 (d, *J* = 8.9 Hz, 1H), 6.04 (d, *J* = 10.5 Hz, 1H), 5.78 (dd, *J* = 8.3, 4.4 Hz, 1H), 5.71 (d, *J* = 9.7 Hz, 1H), 5.59 (s, 1H), 5.54 (d, *J* = 10.1 Hz, 1H), 5.31 (d, *J* = 9.5 Hz, 1H), 4.12–3.99 (m, 1H), 3.96–3.83 (m, 2H), 3.81–3.71 (m, 1H), 3.71–3.64 (m, 1H), 3.61 (s, 3H), 3.57–3.48 (m, 2H), 3.46–3.37 (m, 1H), 2.42–2.14 (m, 4H), 1.64 (m, 2H), 1.41 (s, 3H), 1.37 (s, 3H), 1.30 (s, 9H), 1.18 (q, *J* = 7.0 Hz, 4H), 1.02–0.83 (m, 18H); RP-HPLC: *t*_R_ = 28.35 min; HRMS ESI(+) calcd. for C_32_H_65_N_10_O_7_ 701.50322 [M + H]+, found 701.50324.

Oligourea ester **5b**: Substrates: amine **4** (70 mg, 0.13 mmol), **BB2_b_** (35 mg, 0.15 mmol) and DIPEA (60 μL, 0.38 mmol). Purification: silica gel chromatography (CH_2_Cl_2_:MeOH (*v*/*v*) 95:5);yield: 59% (50 mg); ^1^H NMR (300 MHz, 10% DMSO-d6 in CD_3_CN) δ: 6.51 (d, *J* = 8.7 Hz, 1H), 6.44 (m, 1H), 6.32 (m, 2H), 6.00 (d, *J* = 10.5 Hz, 1H), 5.84–5.74 (m, 1H), 5.74 (d, *J* = 9.7 Hz, 1H), 5.59 (s, 1H), 5.53 (d, *J* = 10.2 Hz, 1H), 5.32 (d, *J* = 9.5 Hz, 1H), 4.13–3.72 (m, 6H), 3.66 (s, 3H), 3.65–3.35 (m, 4H), 2.47–2.11 (m, 4H), 1.65 (m, 2H), 1.30 (s, 9H), 1.24–1.12 (m, 4H), 1.04–0.68 (m, 18H); RP-HPLC: *t*_R_ = 27.47 min; HRMS ESI(+) calcd. for C_30_H_61_N_10_O 673.47192 [M + H]+, found 673.47191.

Oligourea ester **5c**: Substrates: amine **4** (200 mg, 0.36 mmol), **BB2_c_** (110 mg, 0.43 mmol) and DIPEA (188 μL, 1.08 mmol). Purification: silica gel chromatography (CH_2_Cl_2_:MeOH (*v*/*v*) 95:5), yield: 61% (150 mg); ^1^H NMR (300 MHz, 10% DMSO-d6 in CD3CN) δ 6.43 (d, *J* = 7.3 Hz, 1H), 6.41–6.22 (m, 3H), 6.00 (d, *J* = 10.5 Hz, 1H), 5.77 (m, 2H), 5.58 (s, 1H), 5.53 (d, *J* = 10.1 Hz, 1H), 5.31 (d, *J* = 9.5 Hz, 1H), 4.29 (p, *J* = 7.2 Hz, 1H), 4.05 (m, 1H), 3.96–3.71 (m, 3H), 3.67 (s, 3H), 3.55 (m, 3H), 3.41 (m, 1H), 2.41–2.05 (m, 4H), 1.64 (m, 2H), 1.35–1.27 (m, 12H), 1.23–1.12 (m, 4H), 0.99–0.94 (m, 6H), 0.94–0.87 (m, 12H); RP-HPLC: *t*_R_ = 27.55 min; HRMS ESI(+) calcd. for C_31_H_63_N_10_O_7_ 687.48757 [M + H]+, found 687.48776.

Oligourea ester **5d**: Substrates: amine **4** (96 mg, 0.17 mmol), **BB2_d_** (57 mg, 0.21 mmol) and DIPEA (90 μL, 0.51 mmol). Purification: silica gel chromatography (CH_2_Cl_2_:MeOH (*v*/*v*) 96:4), yield: 54% (65 mg); ^1^H NMR (300 MHz, 10% DMSO-d_6_ in CD_3_CN) δ: 6.52 (d, *J* = 9.1 Hz, 1H), 6.40–6.24 (m, 3H), 6.02 (d, *J* = 10.5 Hz, 1H), 5.81 (dd, *J* = 8.2, 4.4 Hz, 1H), 5.73 (d, *J* = 9.8 Hz, 1H), 5.61 (s, 1H), 5.58 (d, *J* = 10.1 Hz, 1H), 5.34 (d, *J* = 9.5 Hz, 1H), 4.19 (dd, *J* = 8.5, 5.2 Hz, 1H), 4.06 (m, 1H), 3.96–3.71 (m, 3H), 3.68 (s, 3H), 3.60 (m, 3H), 3.49–3.35 (m, 1H), 2.46–2.05 (m, 4H), 1.78–1.51 (m, 2H), 1.30 (s, 9H), 1.18 (q, *J* = 6.8 Hz, 4H), 1.01–0.82 (m, 24H); RP-HPLC: *t*_R_ = 29.34 min; HRMS ESI(+) calcd. for C_33_H_67_N_10_O_7_ 715.51887 [M + H]+, found 715.51910.

Oligourea ester **5e**: Substrates: amine **4** (65 mg, 0.12 mmol), **BB2_e_** (41 mg, 0.144 mmol) and DIPEA (63 μL, 0.36 mmol). Purification: silica gel chromatography (CH_2_Cl_2_:MeOH (*v*/*v*) 96:4), yield: 65% (57 mg); ^1^H NMR (300 MHz, 10% DMSO-d_6_ in CD_3_CN) δ: 6.45 (d, *J* = 8.9 Hz, 1H), 6.36–6.21 (m, 3H), 6.01 (d, *J* = 10.5 Hz, 1H), 5.89–5.79 (m, 1H), 5.75 (d, *J* = 9.9 Hz, 1H), 5.60 (s, 1H), 5.54 (d, *J* = 10.1 Hz, 1H), 5.33 (d, *J* = 9.5 Hz, 1H), 4.30 (td, *J* = 8.3, 6.2 Hz, 1H), 4.12–3.99 (m, 1H), 3.99–3.70 (m, 3H), 3.67 (s, 3H), 3.64–3.49 (m, 3H), 3.48–3.34 (m, 1H), 2.45–2.15 (m, 4H), 1.77–1.59 (m, 3H), 1.55–1.46 (m, 2H), 1.30 (s, 9H), 1.23–1.09 (m, 4H), 1.01–0.82 (m, 24H); ^13^C NMR as HSQC (125 MHz, 10% DMSO-d_6_ in CD_3_CN) δ: 52.71, 52.17, 48.31, 48.00, 47.90, 47.74, 46.89, 46.87, 45.67, 45.28, 44.07, 42.67, 42.46, 30.24, 25.89, 25.50, 23.59, 23.39, 23.28, 22.52, 22.45, 21.97, 18.98, 18.40; RP-HPLC: *t*_R_ = 30.49 min; HRMS ESI(+) calcd. for C_34_H_69_N_10_O_7_ 729.53452 [M + H]+, found 729.53485.

Oligourea ester **5f**: Substrates: amine **4** (70 mg, 0.13 mmol), **BB3_f_** (39 mg, 0.16 mmol) and DIPEA (67 μL, 0.39 mmol). Purification: silica gel chromatography (CH_2_Cl_2_:MeOH (*v*/*v*) 95:5), yield: 66% (59 mg); ^1^H NMR (300 MHz, 10% DMSO-d_6_ in CD_3_CN) δ 6.37–6.13 (m, 4H), 5.97 (d, *J* = 10.5 Hz, 1H), 5.77 (dd, *J* = 8.3, 4.4 Hz, 1H), 5.69 (d, *J* = 9.7 Hz, 1H), 5.59 (s, 1H), 5.51 (d, *J* = 10.1 Hz, 1H), 5.31 (d, *J* = 9.6 Hz, 1H), 4.05–3.70 (m, 4H), 3.62 (s, 3H), 3.59–3.43 (m, 4H), 3.34 (q, *J* = 6.3 Hz, 2H), 2.46–2.30 (m, 4H), 2.26–2.11 (m, 2H), 1.65 (m, 2H), 1.30 (s, 9H), 1.25–1.11 (m, 4H), 1.09–0.75 (m, 18H); RP-HPLC: *t*_R_ = 27.92 min; HRMS ESI(+) calcd. for C_31_H_63_N_10_O_7_ 687.48757 [M + H]+, found 687.48774.

Oligourea ester **5g**: Substrates: amine **4** (110 mg, 0.20 mmol), **BB3_g_** (62 mg, 0.24 mmol) and DIPEA (105 μL, 0.60 mmol). Purification: silica gel chromatography (CH_2_Cl_2_:MeOH (*v*/*v*) 95:5), yield: 70% (100 mg); ^1^H NMR (300 MHz, 10% DMSO-d_6_ in CD_3_CN) δ: 6.33 (dd, *J* = 10.0, 3.1 Hz, 1H), 6.27 (dd, *J* = 9.9, 3.3 Hz, 1H), 6.19 (d, *J* = 8.7 Hz, 1H), 6.06 (d, *J* = 8.3 Hz, 1H), 5.99 (d, *J* = 10.4 Hz, 1H), 5.78 (dd, *J* = 8.3, 4.4 Hz, 1H), 5.71 (d, *J* = 9.7 Hz, 1H), 5.60 (s, 1H), 5.53 (d, *J* = 10.2 Hz, 1H), 5.32 (d, *J* = 9.5 Hz, 1H), 4.13–3.97 (m, 2H), 3.96–3.81 (m, 2H), 3.81–3.70 (m, 1H), 3.63 (s, 3H), 3.61–3.47 (m, 3H), 3.47–3.35 (m, 1H), 2.65–2.61 (m, 1H), 2.47–2.28 (m, 3H), 2.28–2.14 (m, 2H), 1.73–1.56 (m, 2H), 1.30 (s, 9H), 1.22–1.15 (m, 4H), 1.13 (d, *J* = 6.6 Hz, 3H), 1.00–0.86 (m, 18H); RP-HPLC: *t*_R_ = 27.84 min; HRMS ESI(+) calcd. for C_32_H_65_N_10_O_7_ 701.50322 [M + H]+, found 701.50334.

Oligourea ester **5h**: Substrates: amine **4** (85 mg, 0.15 mmol), **BB4_h_** (46 mg, 0.18 mmol) and DIPEA (78 μL, 0.45 mmol). Purification: silica gel chromatography (CH_2_Cl_2_:MeOH (*v*/*v*) 95:5), yield: 86% (90 mg); ^1^H NMR (300 MHz, 10% DMSO-d_6_ in CD_3_CN) δ: 6.30 (dd, *J* = 14.7, 10.2 Hz, 2H), 6.18–6.07 (m, 2H), 6.00 (d, *J* = 10.4 Hz, 1H), 5.78 (dd, *J* = 8.2, 4.4 Hz, 1H), 5.68 (d, *J* = 9.7 Hz, 1H), 5.60 (s, 1H), 5.53 (d, *J* = 10.1 Hz, 1H), 5.32 (d, *J* = 9.6 Hz, 1H), 4.06–3.69 (m, 4H), 3.62 (s, 3H), 3.60–3.32 (m, 4H), 3.11 (p, *J* = 6.4 Hz, 2H), 2.47–2.29 (m, 4H), 2.21 (m, 2H), 1.68 (m, 4H), 1.30 (s, 9H), 1.27–1.10 (m, 4H), 1.03–0.80 (m, 18H); RP-HPLC: *t*_R_ = 27.63 min; HRMS ESI(+) calcd. for C_32_H_65_N_10_O_7_ 701.50322 [M + H]+, found 701.50304.

Oligourea ester **5i**: Substrates: amine **4** (60 mg, 0.11 mmol), **BB4_i_** (35 mg, 0.13 mmol) and DIPEA (52 μL, 0.33 mmol). Purification: silica gel chromatography (CH_2_Cl_2_:MeOH (*v*/*v*) 95:5), yield: 78% (68 mg); ^1^H NMR (300 MHz, 10% DMSO-d_6_ in CD_3_CN) δ 7.43–7.10 (m, 5H), 6.36–6.22 (m, 2H), 6.18 (d, *J* = 9.4 Hz, 1H), 5.97 (dd, *J* = 12.5, 9.6 Hz, 2H), 5.77 (dd, *J* = 8.3, 4.4 Hz, 1H), 5.61 (d, *J* = 10.8 Hz, 1H), 5.60 (s, 1H), 5.52 (d, *J* = 10.4 Hz, 1H), 5.31 (d, *J* = 9.6 Hz, 1H), 4.05–3.77 (m, 4H), 3.80–3.66 (m, 1H), 3.60 (s, 3H), 3.61–3.34 (m, 4H), 2.72 (dd, *J* = 6.9, 5.1 Hz, 2H), 2.60–2.50 (m, 1H), 2.42–2.25 (m, 3H), 2.24–2.11 (m, 2H), 1.77–1.60 (m, 2H), 1.59–1.44 (m, 2H), 1.31 (s, 9H), 1.23–1.09 (m, 4H), 0.97–0.85 (m, 18H); RP-HPLC: *t*_R_ = 30.59 min; HRMS ESI(+) calcd. for C_39_H_71_N_10_O_7_ 791.55017 [M + H]+, found 791.55042.

Oligourea ester **5j**: Substrates: amine **4** (70 mg, 0.125 mmol), **BB4_j_** (47 mg, 0.15 mmol) and DIPEA (65 μL, 0.38 mmol). Purification: silica gel chromatography (CH_2_Cl_2_:MeOH (*v*/*v*) 96:4), yield: 57% (54 mg); ^1^H NMR (300 MHz, 10% DMSO-d_6_ in CD_3_CN) δ 6.38–6.21 (m, 2H), 6.16 (d, *J* = 9.2 Hz, 1H), 5.99 (d, *J* = 10.4 Hz, 1H), 5.78 (dd, *J* = 8.3, 4.4 Hz, 1H), 5.69 (d, *J* = 9.3 Hz, 1H), 5.65 (d, *J* = 9.8 Hz, 1H), 5.58 (s, 1H), 5.53 (d, *J* = 10.2 Hz, 1H), 5.30 (d, *J* = 9.5 Hz, 1H), 4.04–3.68 (m, 5H), 3.63 (s, 3H), 3.62–3.47 (m, 3H), 3.47–3.37 (m, 1H), 2.49–2.26 (m, 4H), 2.28–2.12 (m, 2H), 1.74–1.61 (m, 3H), 1.58–1.39 (m, 2H), 1.30 (s, 9H), 1.27–1.10 (m, 6H), 1.07–0.79 (m, 24H); RP-HPLC: *t*_R_ = 30.72 min; HRMS ESI(+) calcd. for C_36_H_73_N_10_O_7_ 757.56582 [M + H]+, found 757.56642.

#### 3.2.4. Hydrolysis of Oligourea Ester **5**

The corresponding ester **5** (1 eq.) was dissolved in MeOH (7 mM). LiOH × H_2_O (10 eq) was dissolved in water (the ratio of MeOH to H_2_O was 4:1) and added to the ester solution. The reaction mixture was stirred for 2 h at RT. The progress of the reaction was monitored by HPLC (for **5g**, the reaction mixture was stirred overnight). The work-up conditions depended on the obtained product/products. To isolate oligourea acid **1**, the solvents were completely and slowly removed under vacuum (water bath at 45 °C). The residue was redissolved in CH_2_Cl_2_ and distilled water. The water layer was extracted with CH_2_Cl_2_ (2×) with the addition of brine because of the formation of an emulsion. The organic layers were removed. HCl (1M) was added to water layer until an acidic pH and it was extracted with CH_2_Cl_2_ until all the product was completely extracted into the organic layer. The solvent was evaporated, and the crude product was dissolved in CH_3_CN, quickly passed through a syringe filter (PA 0.2μm) and lyophilized from CH_3_CN with addition of water. The product of type **1** was obtained as a white solid. To isolate oligourea hydantoin/dihydrouracil **2**, the reaction mixture was diluted with CH_2_Cl_2_ and distilled water. The layers were separated, and the water layer was extracted with CH_2_Cl_2_ with the addition of brine (emulsion formation) until all the cyclic product was completely extracted into the organic layer. The solvent was evaporated, and the crude product was dissolved in CH_3_CN, quickly passed through a syringe filter (PA 0.2μm) and lyophilized from CH_3_CN with the addition of water. The product of type **2** was obtained as a white solid. Copies of ^1^H NMR spectra, as well as HRMS spectra of oligourea acids and oligourea hydantoins/dihydrouracils are given in the Supporting Information, Figures S34–S46 and Figures S81–S93. 

Oligourea acid **1b**: Substrates: **5b** (20 mg, 0.03 mmol) and LiOH × H_2_O (12.6 mg, 0.30 mmol). Yield: 61% (12 mg); ^1^H NMR (300 MHz, 10% DMSO-d_6_ in CD_3_CN) δ: 6.54 (d, *J* = 8.9 Hz, 1H), 6.44 (t, *J* = 5.8 Hz, 1H), 6.40–6.30 (m, 2H), 6.03 (d, *J* = 10.5 Hz, 1H), 5.87–5.76 (m, 2H), 5.64 (s, 1H), 5.56 (d, *J* = 10.1 Hz, 1H), 5.44–5.33 (m, 1H), 4.10–3.98 (m, 1H), 3.98–3.83 (m, 3H), 3.81 (dd, *J* = 5.8, 3.4 Hz, 2H), 3.66–3.48 (m, 3H), 3.48–3.36 (m, 1H), 2.39–2.27 (m, 1H), 2.27–2.16 (m, 2H), 2.16–2.02 (m, 1H), 1.72–1.54 (m, 2H), 1.30 (s, 9H), 1.23–1.12 (m, 4H), 1.00–0.86 (m, 18H); ^13^C NMR as HSQC (125 MHz, 10% DMSO-d_6_ in CD_3_CN) δ: 48.07, 48.06, 47.88, 47.84, 46.90, 46.82, 46.08, 45.43, 43.58, 42.78, 42.71, 29.82, 25.89, 25.70, 23.51, 23.41, 22.48, 18.91, 18.51, 14.42; RP-HPLC: *t*_R_ = 25.52 min; HRMS ESI(+) calcd. for C_29_H_59_N_10_O_7_ [M + H]^+^ 659.4568, found 659.4566.

Oligourea acid **1c**: Substrates: **5c** (9 mg, 0.013 mmol) and LiOH × H_2_O (5.5 mg, 0.13 mmol). Yield: 92% (8 mg), NMR peaks were doubled because of the epimerization of the stereogenic center of the amino acid residue at the C-terminus and the formation of a mixture of diastereomeric products. ^1^H NMR (300 MHz, 10% DMSO-d_6_ in CD_3_CN) δ: 12.08 (bs, 1H), 6.52 (d, *J* = 6.8 Hz, 1H), 6.49–6.37 (m, 2H), 6.36–6.28 (m, 1H), 6.09–5.96 (m, 1H), 5.84–5.77 (m, 1H), 5.73 (d, *J* = 9.7 Hz, 1H), 5.63–5.58 (m, 1H), 5.57–5.50 (m, 1H), 5.36–5.30 (m, 1H), 4.24–4.15 (m, 1H), 4.08–3.98 (m, 1H), 3.96–3.82 (m, 2H), 3.79–3.69 (m, 1H), 3.57–3.48 (m, 3H), 3.48–3.36 (m, 1H), 2.45–2.30 (m, 2H), 2.29–2.15 (m, 2H), 1.72–1.48 (m, 2H), 1.33–1.27 (m, 12H), 1.23–1.13 (m, 4H), 0.98–0.94 (m, 6H), 0.93–0.87 (m, 12H); ^13^C NMR as HSQC (125 MHz, 10% DMSO-d_6_ in CD_3_CN) δ 49.61, 47.91, 47.90, 47.89, 47.86, 46.94, 46.83, 46.07, 45.43, 43.49, 42.68, 29.83, 25.81, 25.59, 23.52, 23.41, 22.48, 18.72, 18.64, 14.38; RP-HPLC: *t*_R_ = 26.23 min; HRMS ESI(+) calcd. for C_30_H_61_N_10_O_7_ [M + H]+ 673.4725, found 673.4722.

Oligourea acid **1f**: Substrates: **5f** (40 mg, 0.06 mmol), LiOH × H_2_O (25.2 mg, 0.6 mmol). Yield: 64% (25 mg); ^1^H NMR (300 MHz, 10% DMSO-d_6_ in CD_3_CN) δ: 11.80 (bs, 1H), 6.37–6.24 (m, 3H), 6.22–6.17 (m, 1H), 5.98 (d, *J* = 10.5 Hz, 1H), 5.78 (dd, *J* = 8.2, 4.3 Hz, 1H), 5.72 (d, *J* = 9.6 Hz, 1H), 5.60 (s, 1H), 5.53 (d, *J* = 10.1 Hz, 1H), 5.33 (d, *J* = 9.6 Hz, 1H), 4.10–3.69 (m, 4H), 3.64–3.38 (m, 4H), 3.37–3.27 (m, 2H), 2.41 (t, *J* = 6.3 Hz, 2H), 2.37–2.06 (m, 4H), 1.72–1.57 (m, 2H), 1.30 (s, 9H), 1.23–1.11 (m, 4H), 1.00–0.80 (m, 18H); ^13^C NMR as HSQC (125 MHz, 10% DMSO-d_6_ in CD_3_CN) δ: 48.29, 47.97, 47.96, 47.78, 45.30, 46.88, 46.87, 45.76, 43.90, 42.71, 36.45, 36.39, 29.86, 25.82, 25.55, 23.55, 23.44, 22.51, 22.49, 19.00, 18.51; RP-HPLC: *t*_R_ = 25.27 min; HRMS ESI(+) calcd. for C_30_H_61_N_10_O_7_ [M + H]+ 673.4725, found 673.4723.

Oligourea acid **1g**: Substrates: **5g** (45 mg, 0.064 mmol) and LiOH × H_2_O (26.8 mg, 0.64 mmol). Yield: 73% (32 mg); ^1^H NMR (300 MHz, 10% DMSO-d_6_ in CD_3_CN) δ: 11.87 (bs, 1H), 6.42–6.27 (m, 2H), 6.26–6.19 (m, 1H), 6.08 (d, *J* = 8.3 Hz, 1H), 6.02 (d, *J* = 10.5 Hz, 1H), 5.84–5.78 (m, 1H), 5.75 (d, *J* = 9.7 Hz, 1H), 5.65 (s, 1H), 5.56 (d, *J* = 10.2 Hz, 1H), 5.41–5.34 (m, 1H), 4.10–3.96 (m, 2H), 3.95–3.82 (m, 2H), 3.81–3.69 (m, 1H), 3.62–3.49 (m, 3H), 3.42 (ddd, *J* = 13.8, 8.4, 3.4 Hz, 1H), 2.47–2.13 (m, 6H), 1.73–1.55 (m, 2H), 1.30 (s, 9H), 1.23–1.15 (m, 4H), 1.14 (d, *J* = 6.7 Hz, 3H), 0.99–0.85 (m, 18H); ^13^C NMR as HSQC (125 MHz, 10% DMSO-d_6_ in CD_3_CN) δ: 48.14, 48.08, 47.75, 47.74, 46.84, 46.81, 45.98, 45.39, 43.60, 43.43, 42.94, 42.71, 29.85, 25.88, 25.54, 23.56, 23.40, 22.53, 22.50, 21.45, 18.96, 18.52; RP-HPLC: *t*_R_ = 26.23 min; HRMS ESI(+) calcd. for C_31_H_63_N_10_O_7_ [M + H]+ 687.4881, found 687.4875.

Oligourea acid **1h**: Substrates: **5h** (70 mg, 0.10 mmol) and LiOH × H_2_O (42 mg, 1.0 mmol). Yield: 84% (58 mg); ^1^H NMR (300 MHz, 10% DMSO-d_6_ in CD_3_CN) δ: 6.47–6.22 (m, 3H), 6.11–6.05 (m, 1H), 6.02 (d, *J* = 10.4 Hz, 1H), 5.81 (dd, *J* = 8.2, 4.4 Hz, 1H), 5.75 (d, *J* = 9.8 Hz, 1H), 5.60 (s, 1H), 5.57 (d, *J* = 10.3 Hz, 1H), 5.33 (d, *J* = 9.5 Hz, 1H), 4.08–3.96 (m, 1H), 3.97–3.69 (m, 3H), 3.67–3.47 (m, 3H), 3.47–3.35 (m, 1H), 3.32–3.15 (m, 1H), 3.12–2.99 (m, 1H), 2.46–2.35 (m, 2H), 2.31 (t, *J* = 7.4 Hz, 2H), 2.27–2.14 (m, 2H), 1.80–1.57 (m, 4H), 1.30 (s, 9H), 1.26–1.11 (m, 4H), 1.02–0.80 (m, 18H); ^13^C NMR as HSQC (125 MHz, 10% DMSO-d_6_ in CD_3_CN) δ: 48.55, 48.02, 47.83, 47.78, 46.84, 46.76, 45.98, 45.49, 43.70, 42.66, 39.52, 32.37, 29.85, 27.33, 25.89, 25.58, 23.54, 23.41, 22.79, 22.47, 18.95, 18.48; RP-HPLC: *t*_R_ = 25.63 min; HRMS ESI(+) calcd. for C_31_H_63_N_10_O_7_ [M + H]+ 687.4881, found 687.4878.

Oligourea acid **1i**: Substrates: **5i** (50 mg, 0.063 mmol) and LiOH × H_2_O (26.4 mg, 0.63 mmol). Yield: 69% (34 mg); ^1^H NMR (300 MHz, 10% DMSO-d_6_ in CD_3_CN) δ: 11.96 (bs, 1H), 7.35–7.13 (m, 5H), 6.56–6.46 (m, 1H), 6.38–6.29 (m, 2H), 6.04 (d, *J* = 10.5 Hz, 1H), 5.88–5.81 (m, 2H), 5.79 (d, *J* = 9.9 Hz, 1H), 5.63 (s, 1H), 5.60 (d, *J* = 10.3 Hz, 1H), 5.36 (d, *J* = 9.5 Hz, 1H), 4.12–3.67 (m, 5H), 3.65–3.47 (m, 3H), 3.47–3.35 (m, 1H), 2.78–2.65 (m, 2H), 2.46–2.31 (m, 3H), 2.32–2.15 (m, 3H), 1.93–1.79 (m, 1H), 1.75–1.56 (m, 2H), 1.54–1.39 (m, 1H), 1.31 (s, 9H), 1.23–1.09 (m, 4H), 1.01–0.80 (m, 18H); ^13^C NMR as HSQC (125 MHz, 10% DMSO-d_6_ in CD_3_CN) δ 130.29, 129.04, 126.90, 51.31, 50.89, 49.01, 47.99, 47.95, 47.79, 46.84, 46.67, 46.13, 45.67, 43.43, 42.98, 42.66, 32.47, 32.05, 29.86, 25.87, 25.61, 23.52, 23.42, 22.91, 22.46, 18.84, 18.49. RP-HPLC: *t*_R_ = 29.48 min; HRMS ESI(+) calcd. for C_38_H_69_N_10_O_7_ [M + H]+ 777.5351, found 777.5349.

Oligourea acid **1j**: Substrates: **5j** (45 mg, 0.059 mmol) and LiOH × H_2_O (24.8 mg, 0.59 mmol). Yield: 75% (33 mg); ^1^H NMR (300 MHz, 10% DMSO-d_6_ in CD_3_CN) δ: 12.00 (s, 1H), 6.57–6.48 (m, 1H), 6.40–6.28 (m, 2H), 6.03 (d, *J* = 10.4 Hz, 1H), 5.88–5.77 (m, 2H), 5.66–5.54 (m, 3H), 5.31 (d, *J* = 9.5 Hz, 1H), 4.11–3.96 (m, 1H), 3.96–3.83 (m, 2H), 3.82–3.71 (m, 2H), 3.70–3.47 (m, 3H), 3.47–3.34 (m, 1H), 2.42–2.23 (m, 4H), 1.91–1.75 (m, 2H), 1.73–1.58 (m, 3H), 1.47–1.36 (m, 2H), 1.30 (s, 9H), 1.25–1.08 (m, 6H), 1.03–0.67 (m, 24H); ^13^C NMR as HSQC (125 MHz, 10% DMSO-d_6_ in CD_3_CN) δ: 48.99, 47.96, 47.93, 47.80, 47.54, 46.87, 46.76, 46.37, 46.23, 45.68, 43.43, 42.66, 33.45, 32.08, 29.83, 25.82, 25.74, 23.51, 22.47, 22.44, 18.44, 18.85; RP-HPLC: *t*_R_ = 29.49 min; HRMS ESI(+) calcd. for C_35_H_71_N_10_O_7_ [M + H]+ 743.5507, found 743.5504.

Oligourea hydantoin **2a**: Substrates: **5a** (40 mg, 0.057 mmol) and LiOH × H_2_O (23.9 mg, 0.57 mmol). Yield: 87% (35 mg); ^1^H NMR (300 MHz, 10% DMSO-d_6_ in CD_3_CN) δ: 7.34 (s, 1H), 6.01–5.94 (m, 1H), 5.89 (dd, *J* = 9.6, 3.3 Hz, 1H), 5.83 (d, *J* = 8.9 Hz, 1H), 5.72 (dd, *J* = 7.8, 4.6 Hz, 1H), 5.66 (d, *J* = 9.2 Hz, 1H), 5.59 (s, 1H), 5.52 (d, *J* = 9.4 Hz, 1H), 5.35 (d, *J* = 9.0 Hz, 1H), 4.15–3.97 (m, 1H), 3.92–3.69 (m, 3H), 3.61–3.37 (m, 2H), 3.34 (dd, *J* = 6.9, 1.9 Hz, 2H), 3.32–3.20 (m, 1H), 2.69–2.63 (m, 1H), 2.59–2.51 (m, 1H), 2.41 (q, *J* = 10.9 Hz, 1H), 1.77–1.54 (m, 2H), 1.34 (d, *J* = 1.5 Hz, 6H), 1.28 (s, 9H), 1.25–1.10 (m, 4H), 1.02 (d, *J* = 6.9 Hz, 3H), 0.98 (d, *J* = 6.7 Hz, 3H), 0.95–0.82 (m, 12H); ^13^C NMR as HSQC (125 MHz, 10% DMSO-d_6_ in CD_3_CN) δ: 47.89, 47.35, 46.89, 46.48, 46.44, 46.36, 46.14, 43.90, 42.99, 42.90, 29.86, 25.78, 25.47, 25.16, 24.97, 23.64, 23.26, 22.51, 22.18, 19.67, 18.99; RP-HPLC: *t*_R_ = 25.28 min; HRMS ESI(+) calcd. for C_31_H_61_N_10_O_6_ [M + H]+ 669.4776, found 669.4769.

Oligourea hydantoin **2b**: Substrates: **5b** (15 mg, 0.022 mmol) and LiOH × H_2_O (9.2 mg, 0.22 mmol). Yield: 55% (8 mg); ^1^H NMR (300 MHz, 10% DMSO-d_6_ in CD_3_CN) δ: 7.13 (s, 1H), 6.01–5.93 (m, 1H), 5.88–5.83 (m, 1H), 5.81 (d, *J* = 8.7 Hz, 1H), 5.73–5.66 (m, 1H), 5.63 (d, *J* = 9.2 Hz, 1H), 5.54 (s, 1H), 5.48 (d, *J* = 10.3 Hz, 1H), 5.27 (d, *J* = 9.3 Hz, 1H), 4.11–4.00 (m, 1H), 3.94–3.86 (m, 1H), 3.83 (dd, *J* = 3.5, 1.2 Hz, 2H), 3.80–3.67 (m, 1H), 3.61–3.43 (m, 3H), 3.37 (d, *J* = 6.8 Hz, 2H), 3.35–3.25 (m, 1H), 2.53 (d, *J* = 4.8 Hz, 1H), 2.45–2.27 (m, 1H), 2.16–2.00 (m, 1H), 1.77–1.51 (m, 2H), 1.28 (s, 9H), 1.23 (t, *J* = 7.4 Hz, 2H), 1.19–1.12 (m, 2H), 1.02 (d, *J* = 6.9 Hz, 3H), 0.99 (d, *J* = 6.7 Hz, 3H), 0.93–0.83 (m, 12H); ^13^C NMR as HSQC (125 MHz, 10% DMSO-d_6_ in CD_3_CN) δ: 47.84, 47.24, 47.01, 46.85, 46.53, 46.44, 46.29, 46.06, 43.79, 42.92, 42.83, 29.81, 25.75, 25.49, 23.60, 22.28, 22.13, 19.59, 19.00, 14.39; RP-HPLC: *t*_R_ = 24.88 min; HRMS ESI(+) calcd. for C_29_H_57_N_10_O_6_ [M + H]+ 641.4463, found 641.4456.

Oligourea hydantoin **2c**: Substrates: **5c** (30 mg, 0.044 mmol) and LiOH × H_2_O (18.5 mg, 0.44 mmol). Yield: 65% (19 mg); NMR peaks were doubled because of the epimerization of the stereogenic center of the amino acid residue at the C-terminus and the formation of a mixture of diastereomeric products, ^1^H NMR (300 MHz, 10% DMSO-d_6_ in CD_3_CN) δ: 7.27 (s, 1H), 6.03–5.92 (m, 1H), 5.91–5.78 (m, 2H), 5.73–5.67 (m, 1H), 5.65 (d, *J* = 10.8 Hz, 1H), 5.55 (s, 1H), 5.52–5.45 (m, 1H), 5.30 (d, *J* = 9.4 Hz, 1H), 4.13–3.93 (m, 1H), 3.94–3.81 (m, 3H), 3.80–3.70 (m, 1H), 3.65–3.41 (m, 2H), 3.41–3.30 (m, 2H), 3.32–3.26 (m, 1H), 2.76–2.64 (m, 1H), 2.45–2.03 (m, 2H), 1.71–1.52 (m, 2H), 1.28 (s, 9H), 1.26–1.13 (m, 4H), δ 1.12 (d, *J* = 6.2 Hz, 3H), 1.02 (d, *J* = 6.9 Hz, 3H), 0.98 (d, *J* = 6.7 Hz, 3H), 0.95–0.82 (m, 12H); ^13^C NMR as HSQC (125 MHz, 10% DMSO-d_6_ in CD_3_CN) δ: 53.36, 47.87, 47.31, 46.84, 46.47, 46.31, 46.08, 43.84, 42.91, 42.89, 29.83, 25.76, 25.48, 23.62, 22.35, 22.29, 19.65, 19.02, 17.69; RP-HPLC: *t*_R_ = 25.28 min; HRMS ESI(+) calcd. for C_30_H_59_N_10_O_6_ [M + H]+ 655.4619, found 655.4614

Oligourea hydantoin **2d**: Substrates: **5d** (30 mg, 0.042 mmol) and LiOH × H_2_O (17.6 mg, 0.42 mmol). Yield: 87% (25 mg); NMR peaks were doubled because of the epimerization of the stereogenic center of the amino acid residue at the C-terminus and the formation of a mixture of diastereomeric products, ^1^H NMR (300 MHz, 10% DMSO-d_6_ in CD_3_CN) δ: 7.32 (d, *J* = 7.3 Hz, 1H), 6.03–5.94 (m, 1H), 5.90–5.77 (m, 2H), 5.71–5.61 (m, 2H), 5.56–5.49 (m, 1H), 5.50–5.43 (m, 1H), 5.25 (d, *J* = 9.3 Hz, 1H), 4.06 (m, 1H), 3.94–3.83 (m, 3H), 3.81–3.66 (m, 1H), 3.59–3.46 (m, 2H), 3.36 (d, *J* = 7.1 Hz, 2H), 3.34–3.30 (m, 1H), 2.57–2.53 (m, 1H), 2.41–2.30 (m, 1H), 2.16–2.07 (m, 1H), 1.71–1.50 (m, 3H), 1.29 (s, 9H), 1.24–1.10 (m, 4H), 1.04–0.96 (m, 6H), 0.93–0.82 (m, 18H); ^13^C NMR as HSQC (125 MHz, 10% DMSO-d_6_ in CD_3_CN) δ 62.90, 47.81, 47.32, 46.87, 46.47, 46.42, 46.35, 46.11, 43.62, 42.97, 42.90, 30.85, 29.84, 25.80, 25.42, 23.70, 22.87, 22.54, 22.43, 19.61, 19.07, 16.65, 14.45; RP-HPLC: *t*_R_ = 26.19 min; HRMS ESI(+) calcd. for C_32_H_63_N_10_O_6_ [M + H]+ 683.4932, found 683.4923

Oligourea hydantoin **2e**: Substrates: **5e** (40 mg, 0.055 mmol) and LiOH × H_2_O (23.0 mg, 0.55 mmol). Yield: 89% (34 mg) NMR peaks were doubled because of the epimerization of the stereogenic center of the amino acid residue at the C-terminus and the formation of a mixture of diastereomeric products, ^1^H NMR (300 MHz, 10% DMSO-d_6_ in CD_3_CN) δ 7.45 (d, *J* = 11.1 Hz, 1H), 6.05–5.94 (m, 1H), 5.93–5.78 (m, 2H), 5.70 (dd, *J* = 7.7, 4.5 Hz, 1H), 5.63 (dd, *J* = 9.3, 4.5 Hz, 1H), 5.55 (d, *J* = 2.2 Hz, 1H), 5.54–5.43 (m, 1H), 5.30 (d, *J* = 9.3 Hz, 1H), 4.14–3.98 (m, 1H), 3.97–3.81 (m, 2H), 3.82–3.70 (m, 1H), 3.59–3.43 (m, 3H), 3.40–3.16 (m, 2H), 3.32–3.22 (m, 1H), 2.56–2.52 (m, 2H), 2.45–2.30 (m, 1H), 1.90–1.76 (m, 1H), 1.77–1.47 (m, 2H), 1.28 (s, 9H), 1.27–1.10 (m, 6H), 1.09–0.77 (m, 24H); ^13^C NMR as HSQC (125 MHz, CD_3_CN) δ 56.35, 47.93, 47.33, 46.95, 46.60, 46.50, 46.40, 46.28, 43.90, 42.88, 42.78, 42.25, 29.85, 25.81, 25.58, 25.53, 25.52, 23.60, 23.58, 22.57, 22.11, 21.74, 21.60, 19.61, 18.95; RP-HPLC: *t*_R_ = 27.09 min; HRMS ESI(+) calcd. for C_33_H_65_N_10_O_6_ [M + H]+ 697.5088, found 697.5085

Oligourea dihydrouracil **2g**: Substrates: **5g** (35 mg, 0.050 mmol) and LiOH × H_2_O (21.0 mg, 0.50 mmol). Yield: 42% (4 mg); ^1^H NMR (300 MHz, 10% DMSO-d_6_ in CD_3_CN) δ 6.75 (s, 1H), 6.06–5.98 (m, 1H), 5.90–5.78 (m, 2H), 5.71–5.62 (m, 1H), 5.56–5.48 (m, 2H), 5.43 (d, *J* = 9.5 Hz, 1H), 5.24 (d, *J* = 9.4 Hz, 1H), 4.15–4.00 (m, 1H), 3.99–3.82 (m, 2H), 3.82–3.64 (m, 2H), 3.61 (d, *J* = 5.6 Hz, 2H), 3.59–3.40 (m, 2H), 3.40–3.29 (m, 1H), 2.78–2.66 (m, 1H), 2.46–2.39 (m, 1H), 2.39–2.17 (m, 2H), 2.15–2.03 (m, 1H), 1.76–1.55 (m, 2H), 1.29 (s, 9H), 1.18 (d, *J* = 6.4 Hz, 3H), 1.20–1.11 (m, 4H), 1.02 (d, *J* = 6.9 Hz, 3H), 0.98 (d, *J* = 6.7 Hz, 3H), 0.95–0.77 (m, 12H). ^13^C NMR as HSQC (125 MHz, 10% DMSO-d_6_ in CD_3_CN) δ: 47.80, 47.46, 46.91, 46.67, 46.61, 46.21, 46.17, 44.37, 43.46, 43.07, 42.90, 39.89, 29.83, 25.78, 25.61, 23.98, 23.62, 22.49, 22.35, 20.65, 19.81, 19.03. RP-HPLC: *t*_R_ = 25.90 min; HRMS ESI(+) calcd. for C_31_H_61_N_10_O_6_ [M + H]+ 669.4775, found 669.4772

### 3.3. Stability Studies of Oligourea-Hydantoins under Alkaline Conditions

Stock solutions of compounds **2b** or **2e** were prepared at a concentration of 9.4 mM in MeOH and a stock solution of LiOH was prepared at a concentration of 94 mM in H_2_O (from LiOH×H_2_O). Next, 0.1 mL of the solution of LiOH in H_2_O was added to 0.1 mL of **2b** or **2e**. The samples were incubated (with mixing) at 45 °C or RT (set as 23 °C) in the thermomixer (Eppendorf). At specific time intervals (30 min, 1 h, 2 h, 4 h or 24 h), aliquots were collected and the samples for RP-HPLC analyses were prepared as follows: 25 μL of the sample was added to 150μL of MeOH. It was checked by HPLC that the reaction at such a dilution was slow enough not to proceed before injection. 

### 3.4. Circular Dichroism

Circular dichroism (CD) experiments were performed on a Jasco J-1500 spectrometer. Data are expressed in terms of the total molar ellipticity (deg·cm^2^·dmol^−1^). CD spectra of oligomers (0.2 mM) were acquired in 2,2,2-trifluoroethanol between 190 and 250 nm using a rectangular quartz cell with a path length of 1 mm. 

### 3.5. Crystallographic Data

See the supporting information for details (Table S3). CCDC-2237296 contains the supplementary crystallographic data for this paper. These data can be obtained free of charge from The Cambridge Crystallographic Data Centre via www.ccdc.cam.ac.uk/data_request/cif (accessed on 20 January 2023).

## 4. Conclusions

Herein, we have investigated the reaction of oligoureas with methyl esters at the C-terminus under basic conditions. Under such conditions, two possible transformations can occur: the hydrolysis of methyl ester with the formation of an oligourea acid or cyclization of the final residue and the formation of 5- or 6-membered rings at the C-terminus of the oligomer. We discovered that the result of the reaction is strongly dependent on the structure of the terminal residue. Oligoureas modified with α- and β-amino acid esters form two types of products, whereas γ-amino acid esters undergo only a hydrolysis reaction. Moreover, for Gly and Ala derivatives, the yield of isolated products depends on the work-up conditions, influenced strongly by the chemical stability of the hydantoin ring. 

Conformational studies of short acid and hydantoin oligoureas in solution as well as in the solid state revealed that both types of compound fold into 2.5 helices. The stability of the secondary structure of the acid derivatives was observed to be high, as confirmed by NMR and ECD experiments. The hydantoin ring seems to be noticeably labile and is not involved in the intramolecular hydrogen bonding network. Even in the crystal structure, it occupies two positions, either folded “inside” the helix or flipped outside of the helix. 

The C-terminal-modified oligoureas described in this work expand the family of synthetic oligomers able to fold into stable structures. Moreover, these compounds may find application in molecular recognition and in the formation of supramolecular polymers or more complicated architectures [36,37], as well as in the field of biochemistry, as many hydantoin derivatives show biological activity [28,29]. The hydantoin foldamer hybrids reported here seem to be promising candidates for further studies, as they contain a helical portion which, when properly designed, may be able to mimic biologically active peptides, with the modifiable hydantoin moiety providing a further avenue to enable the design of molecules with biological activities.

## Figures and Tables

**Table 1:** ^1^H NMR diastereotopicity (Δd) values for main chain methylene protons for compounds **2a** and **1f** (the Δd values are given in ppm).

Compound	Leu1u	Ala2u	Ala3u	Leu4u
**2a**	0.83	1.12	0.85	0
**1f**	1.11	1.33	1.33	1.15

## Data Availability

All data generated or analyzed during this study are included in this published article and its Supplementary Information.

## Supplementary Materials

The following supporting information can be downloaded at: https://www.mdpi.com/article/10.3390/ijms24076806/s1. References [38,39,40,41,42,43,44,45,46] are cited in the supplementary materials.
