# Supplementary material for: C-Terminal-Modified Oligourea Foldamers as a Result of Terminal Methyl Ester Reactions under Alkaline Conditions
Source: Int J Mol Sci. 2023 Apr 6;24(7):6806. doi: 10.3390/ijms24076806 (PMC10094884; doi:10.3390/ijms24076806)
Supplement: Supplementary file 1 [file ijms-24-06806-s001.zip › ijms-2329752-supplementary.pdf]

C-Terminal-Modified Oligourea Foldamers as a Result of Terminal Methyl Ester Reactions under Alkaline Conditions

Supporting Information

Katarzyna Kedzia,[1] Lukasz Dobrzycki,[2] Marcin Wilczek,[1] Karolina Pulka-Ziach[1] *

[1] Faculty of Chemistry, University of Warsaw, Pasteura 1, 02-093 Warsaw, Poland

[2] Laboratory of Advanced Crystal Engineering, Faculty of Chemistry, University of Warsaw, Żwirki i Wigury 101, 02-089 Warsaw, Poland

* karola@chem.uw.edu.pl

Table of contents

1. **Building blocks and final compounds** 2
2. **NMR spectra of building blocks BB1-BB4** 3
3. **NMR spectra of oligourea-esters of type 5** 13
4. **Nmr spectra of oligourea-acids 1.** 18
5. **NMR spectra of oligourea-hydantoin/dihydrouracil 2.** 21
6. **Signal assignments of oligourea-acids 1 and oligourea-hydantoin/dihydrouracil 2 based on 2D NMR spectra.** 24
7. **Additional 1D and 2D NMR spectra** 29
8. **RP-HPLC chromatograms of reaction mixtures of oligourea-esters 5 under alkaline conditions** 30
9. **HRMS of building blocks, oligourea esters and final compounds** 33
10. **CD spectra of selected oligourea-esters 5, oligourea-acids 1 and oligourea-hydantoin/dihydrouracil 2** 44
11. **Additional HPLC chromatograms** 45
12. **Crystallography** 45
13. **References** 49

1. Building blocks and final compounds

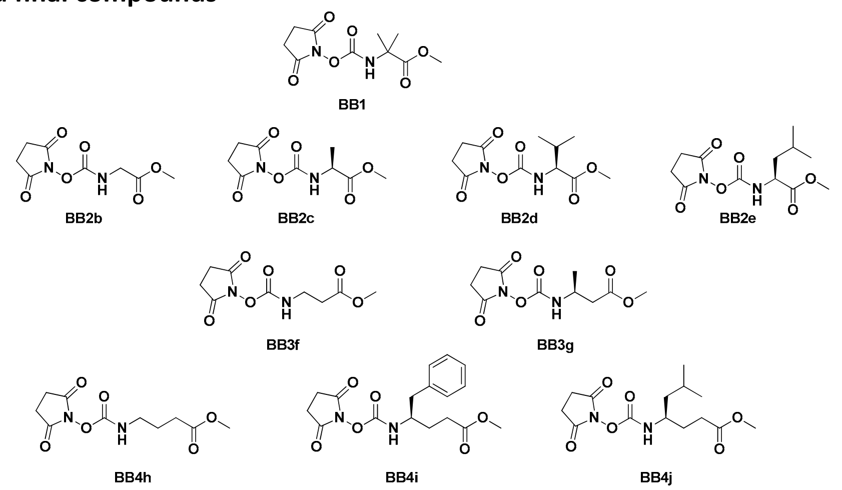

Figure S 1. Succinimidyl carbamate derivatives of methyl esters of α-, β-, γ-amino acids

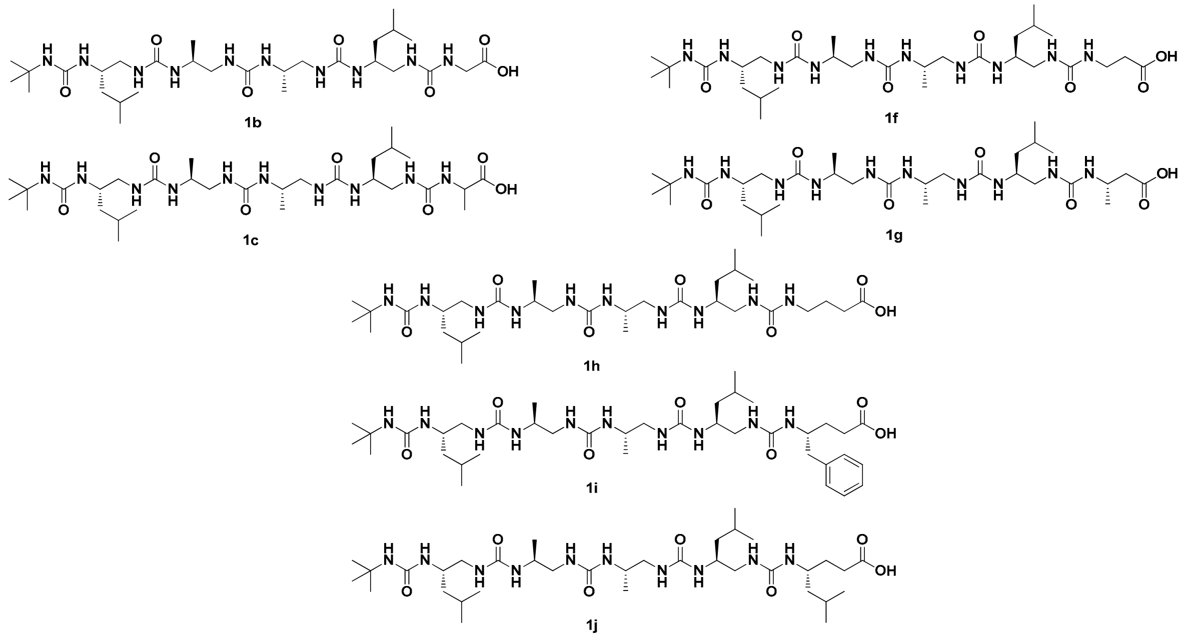

Figure S 2. Structures of oligourea-acids **1**

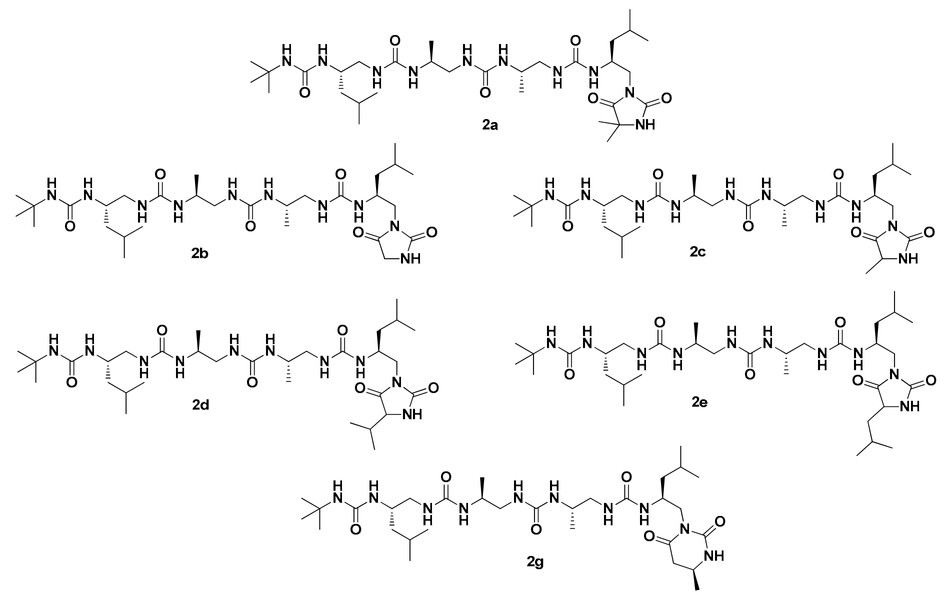

Figure S 3. Structures of oligourea-hydantoins and oligourea-dihydrouracil **2**

2. NMR spectra of building blocks BB1-BB4

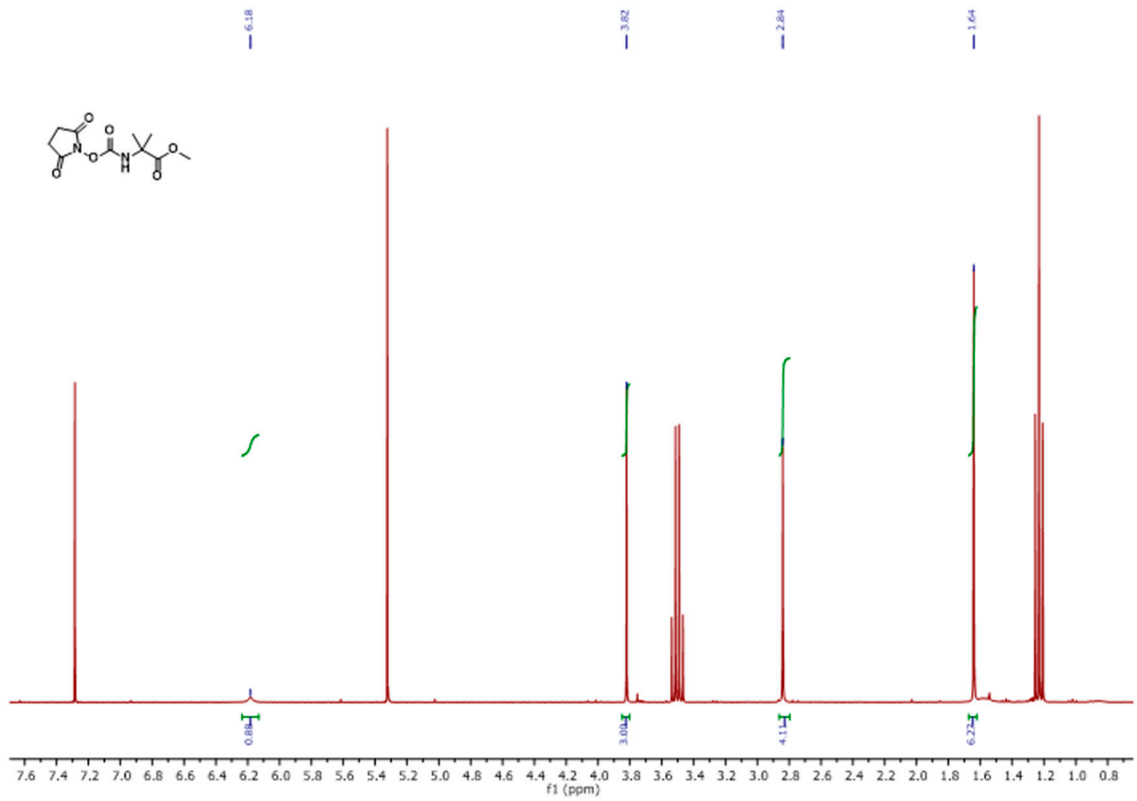

Figure S 4. ^{1}H NMR spectrum of **BB1** in $CDCl_3$ (300 MHz)

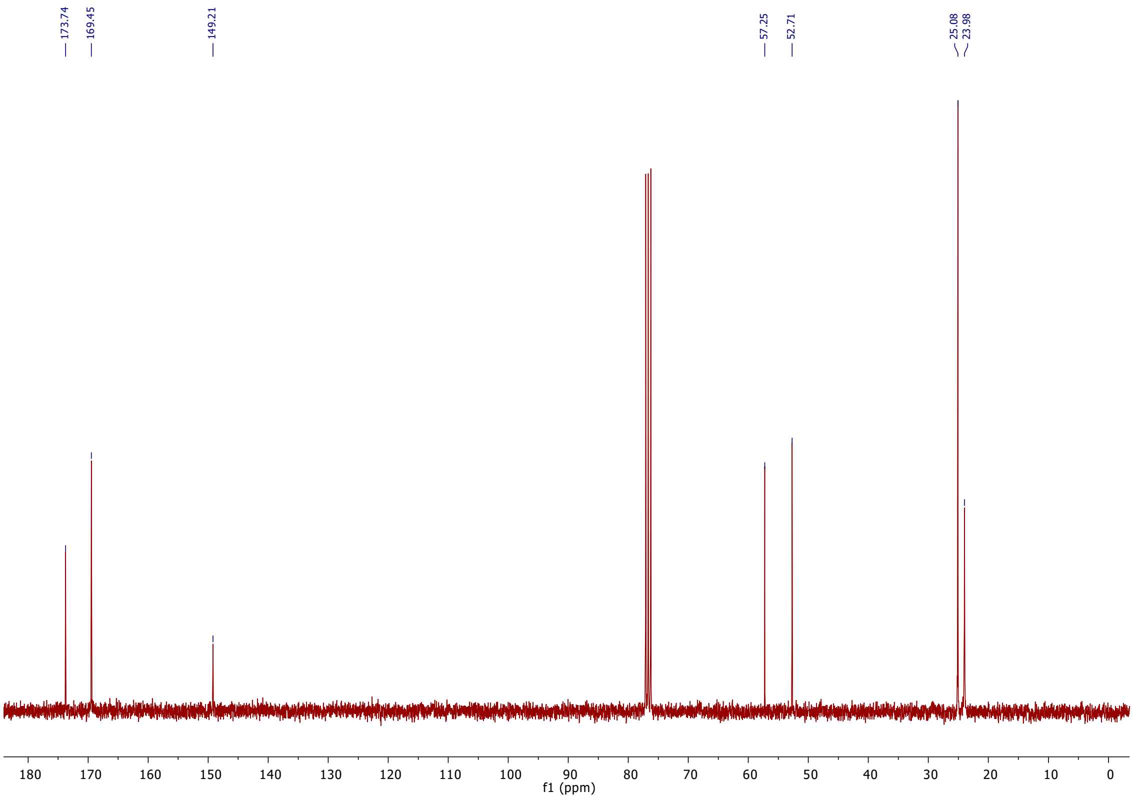

Figure S 5. ^{13}C NMR spectrum of **BB1** in $CDCl_3$ (300 MHz)

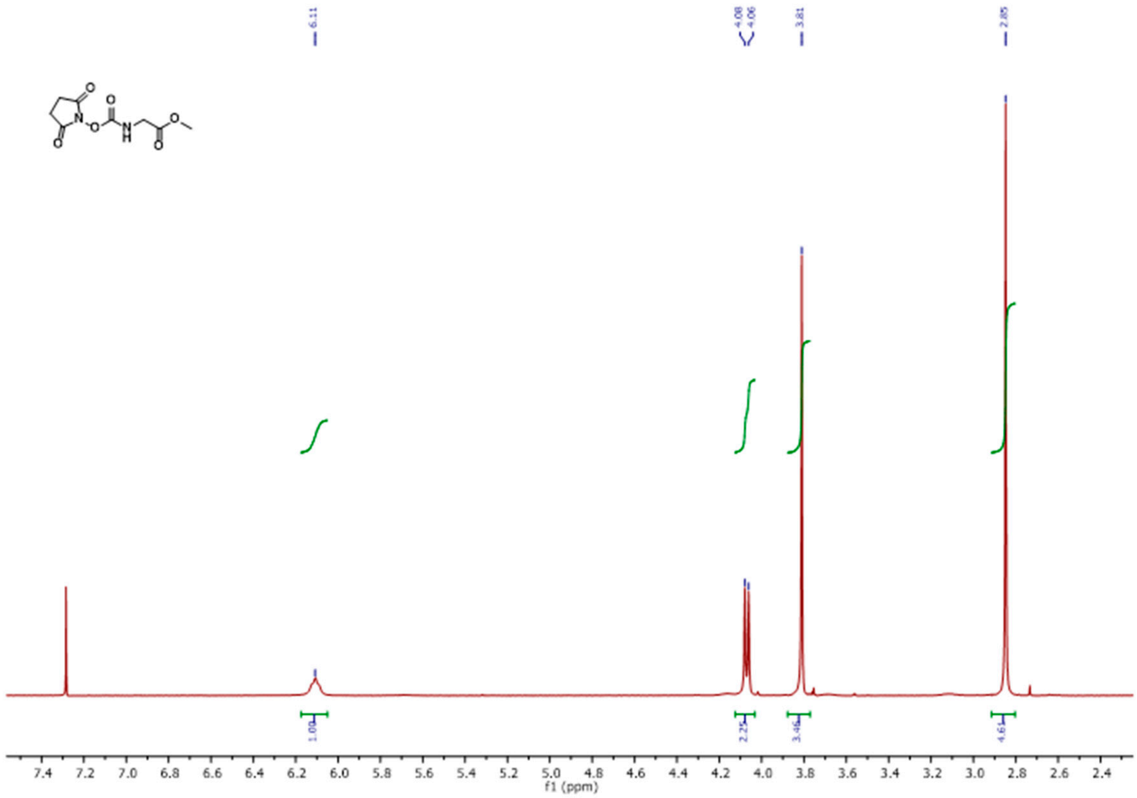

Figure S 6. ^{1}H NMR spectrum of **BB2b** in $CDCl_3$ (300 MHz)

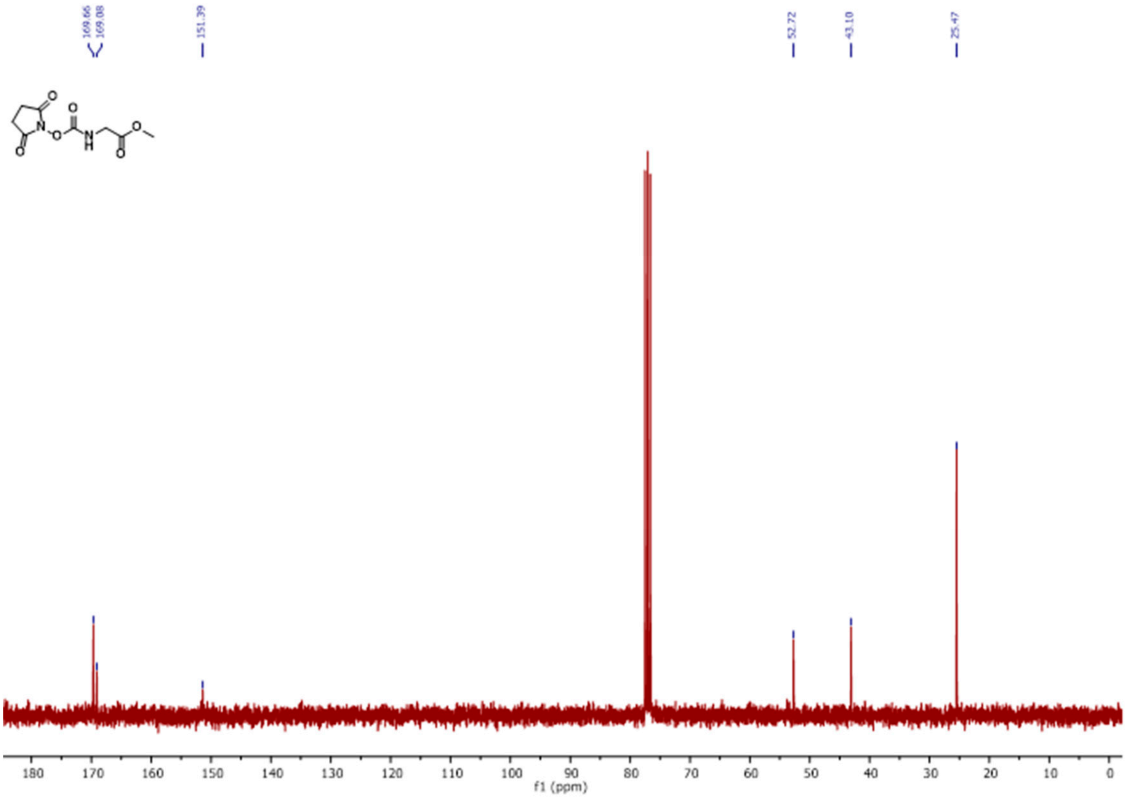

Figure S 7. ^{13}C NMR spectrum of **BB2b** in $CDCl_3$ (300 MHz)

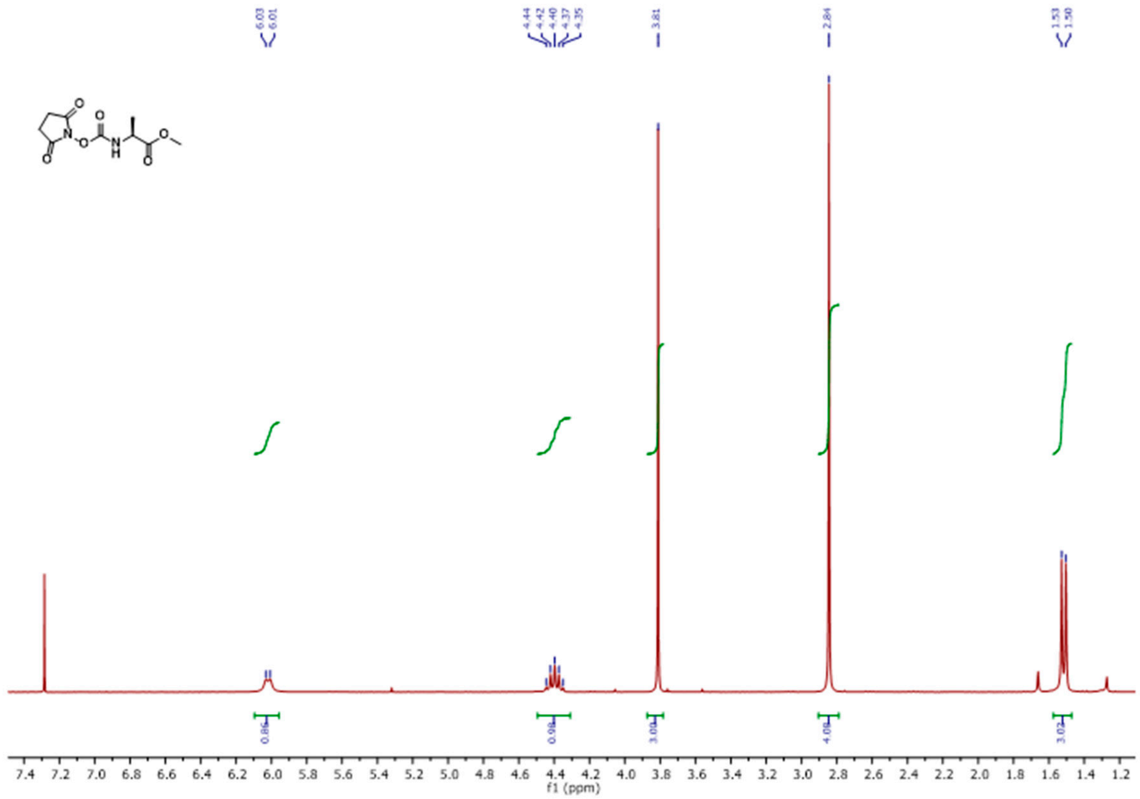

Figure S 8. ^{1}H NMR spectrum of **BB2c** in $CDCl_3$ (300 MHz)

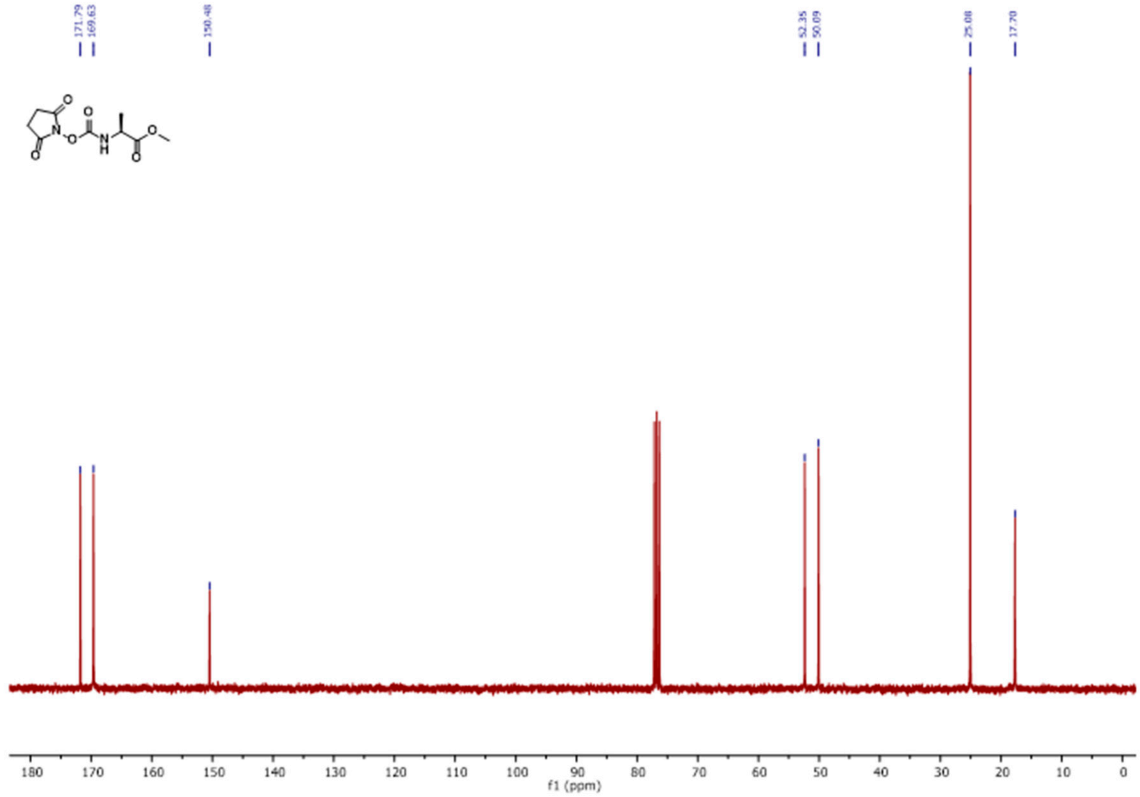

Figure S 9. ^{13}C NMR spectrum of **BB2c** in $CDCl_3$ (300 MHz)

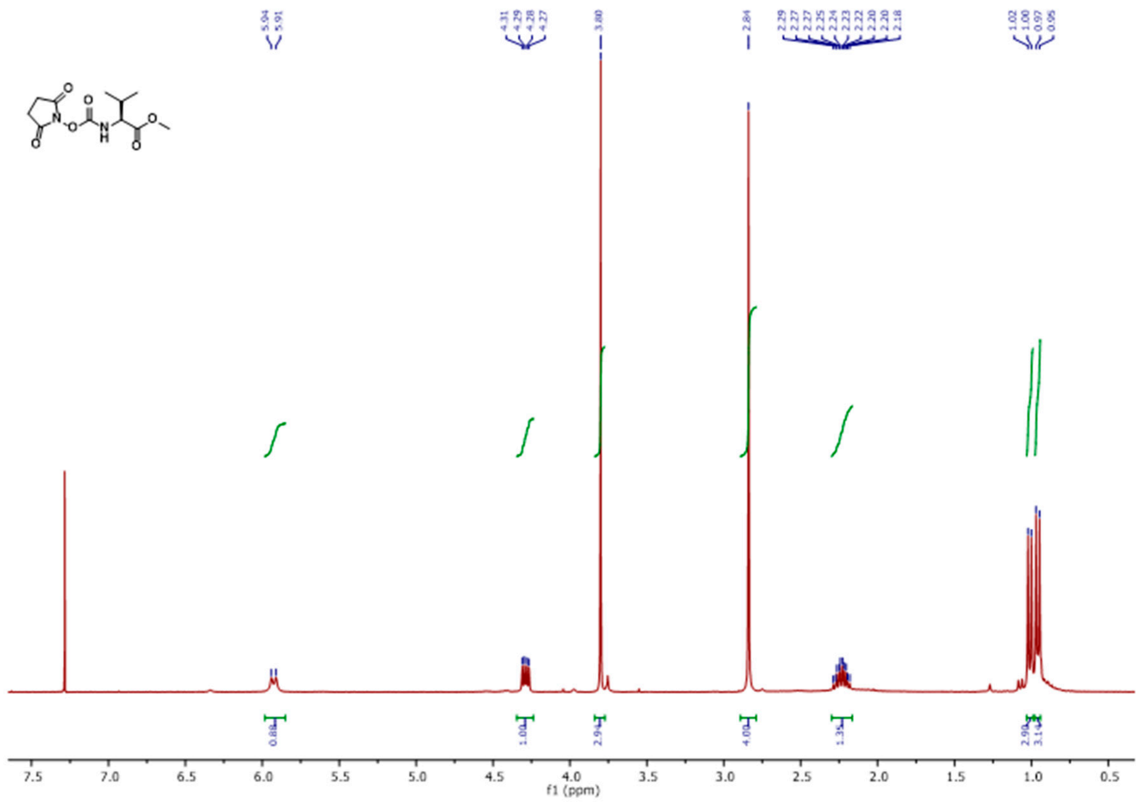

Figure S 10. ^{1}H NMR spectrum of **BB2d** in $CDCl_3$ (300 MHz)

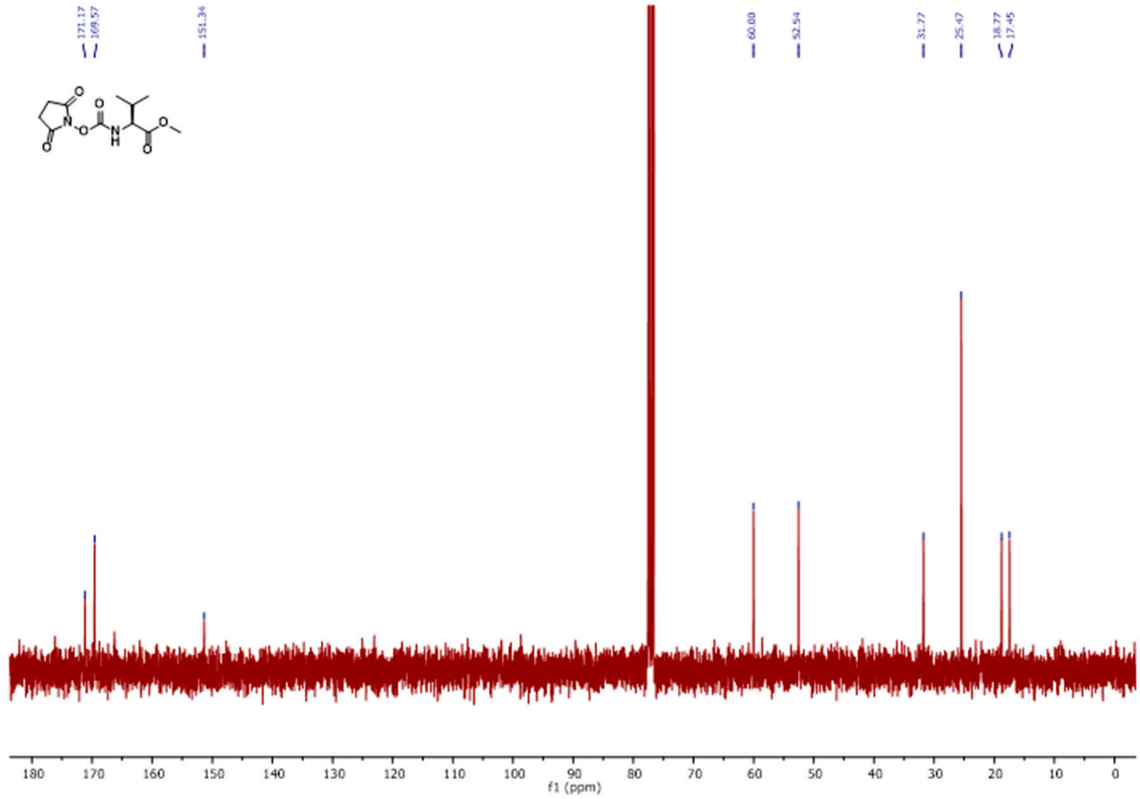

Figure S 11. ^{13}C NMR spectrum of **BB2d** in $CDCl_3$ (300 MHz)

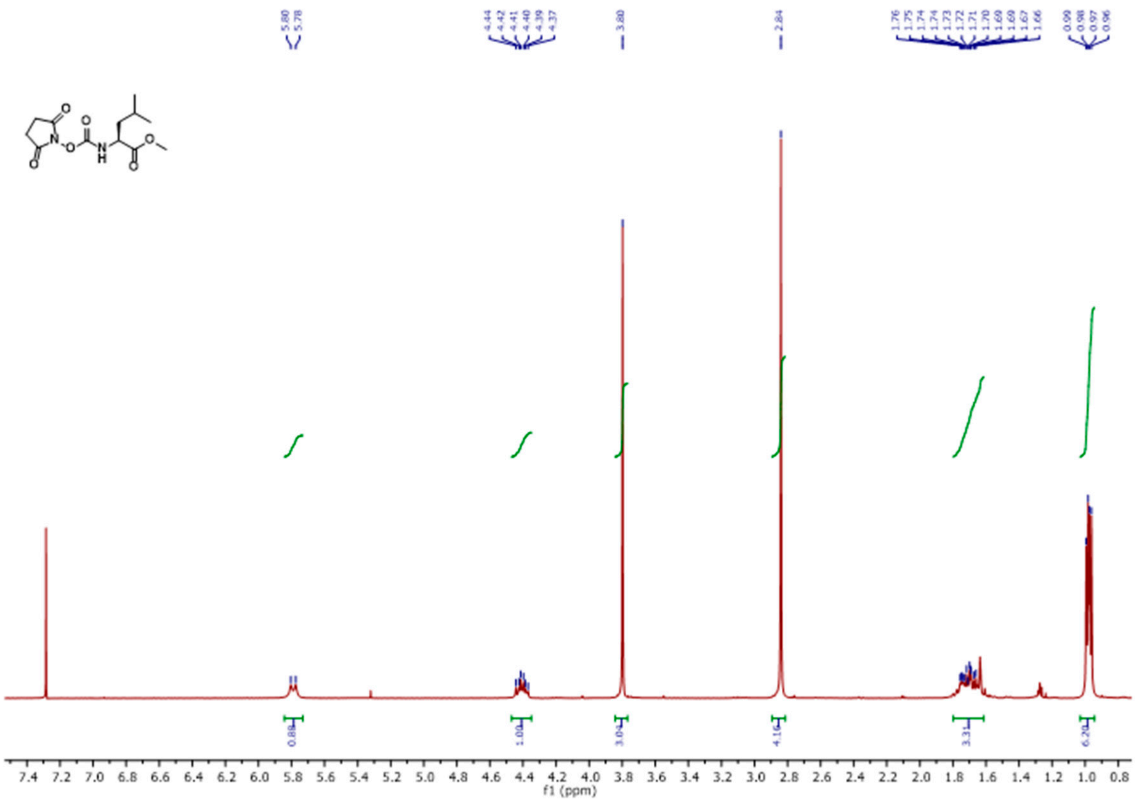

Figure S 12. ^{1}H NMR spectrum of **BB2e** in $CDCl_3$ (300 MHz)

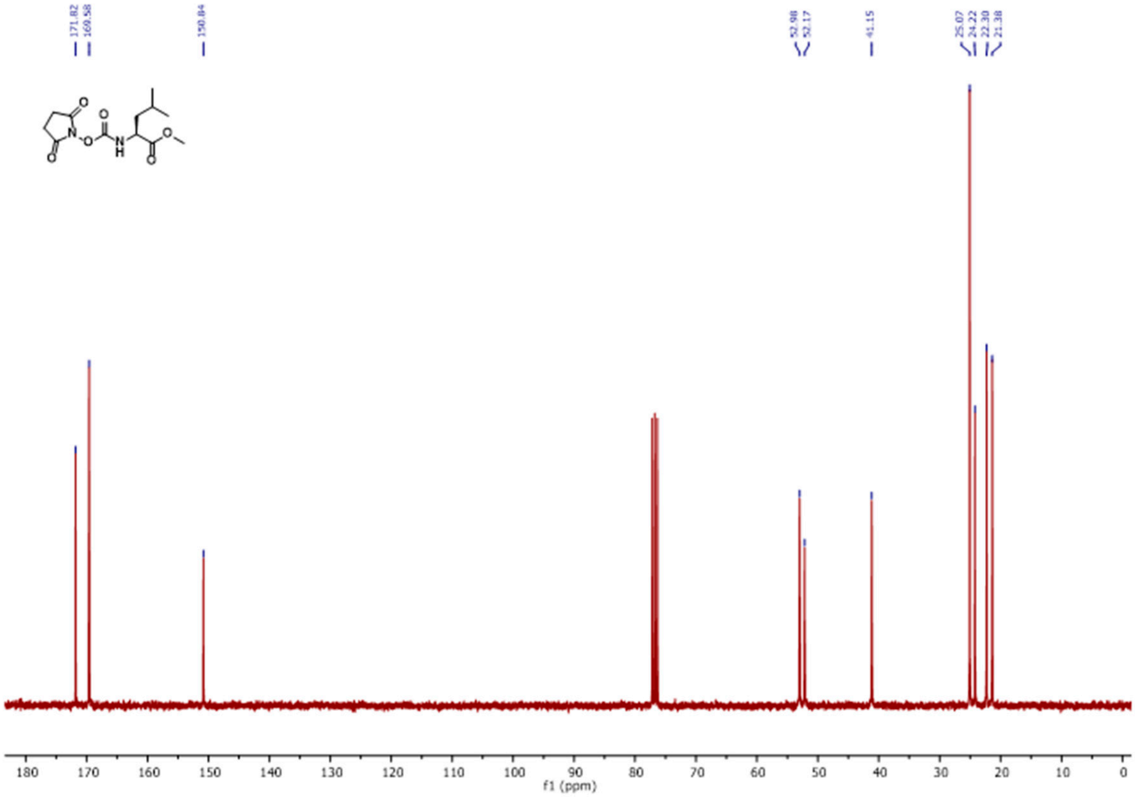

Figure S 13. ^{13}C NMR spectrum of **BB2e** in $CDCl_3$ (300 MHz)

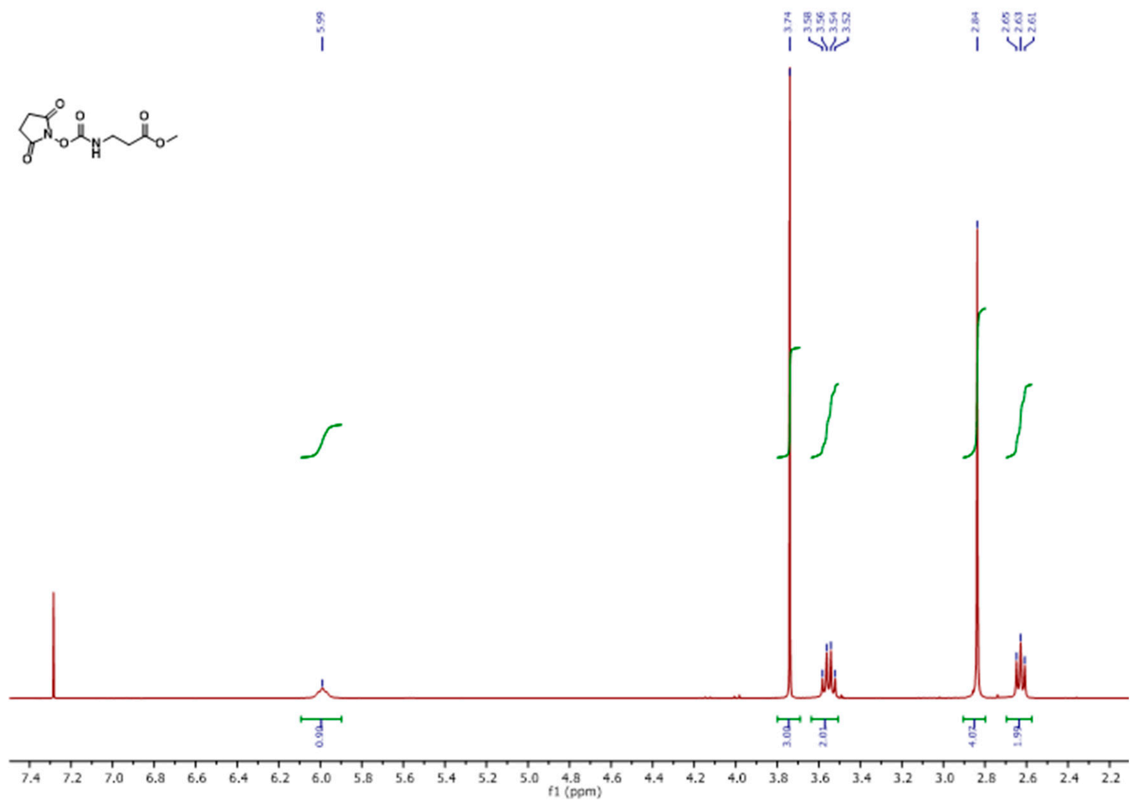

Figure S 14. ^{1}H NMR spectrum of **BB3f** in $CDCl_3$ (300 MHz)

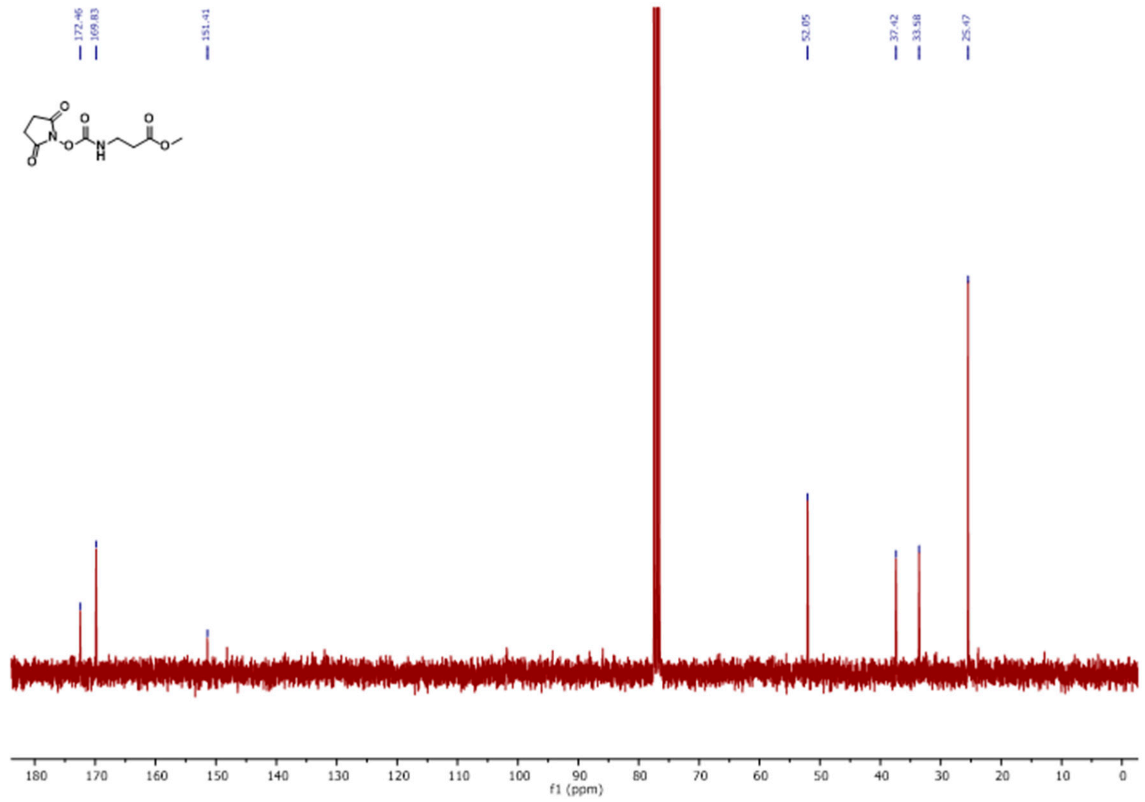

Figure S 15. ^{13}C NMR spectrum of **BB3f** in $CDCl_3$ (300 MHz)

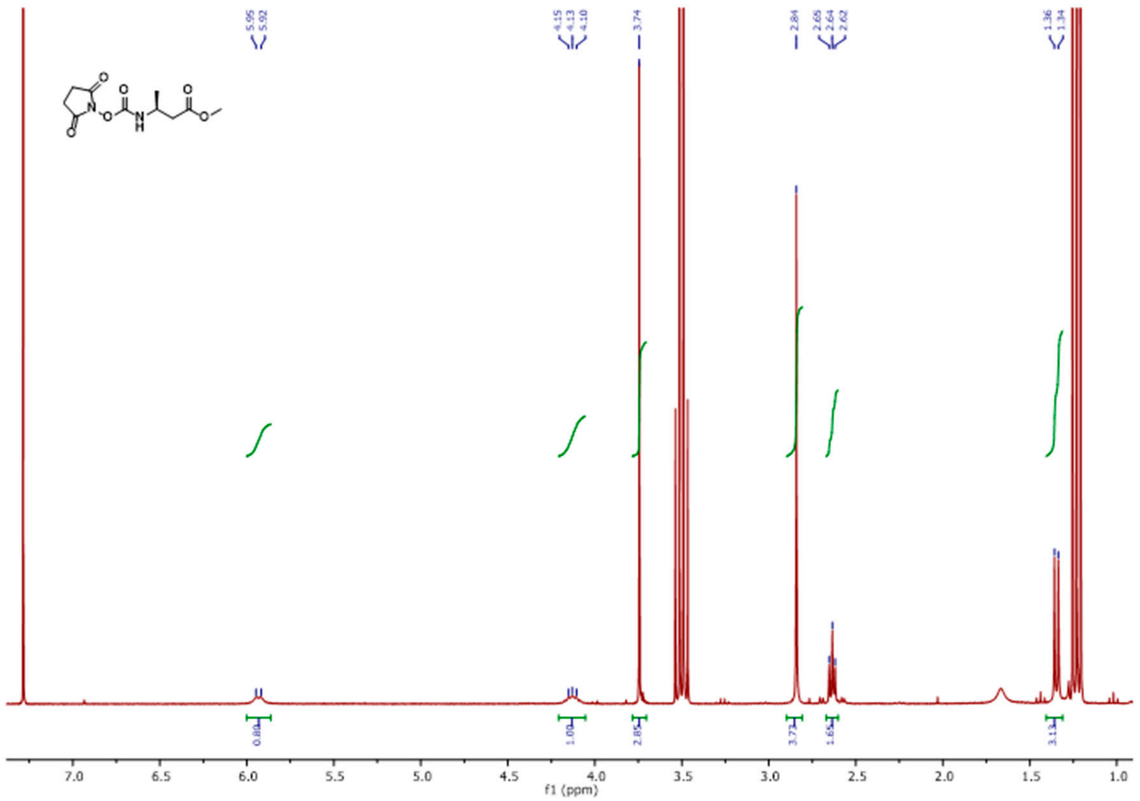

Figure S 16. ^{1}H NMR spectrum of **BB3g** in $CDCl_3$ (300 MHz)

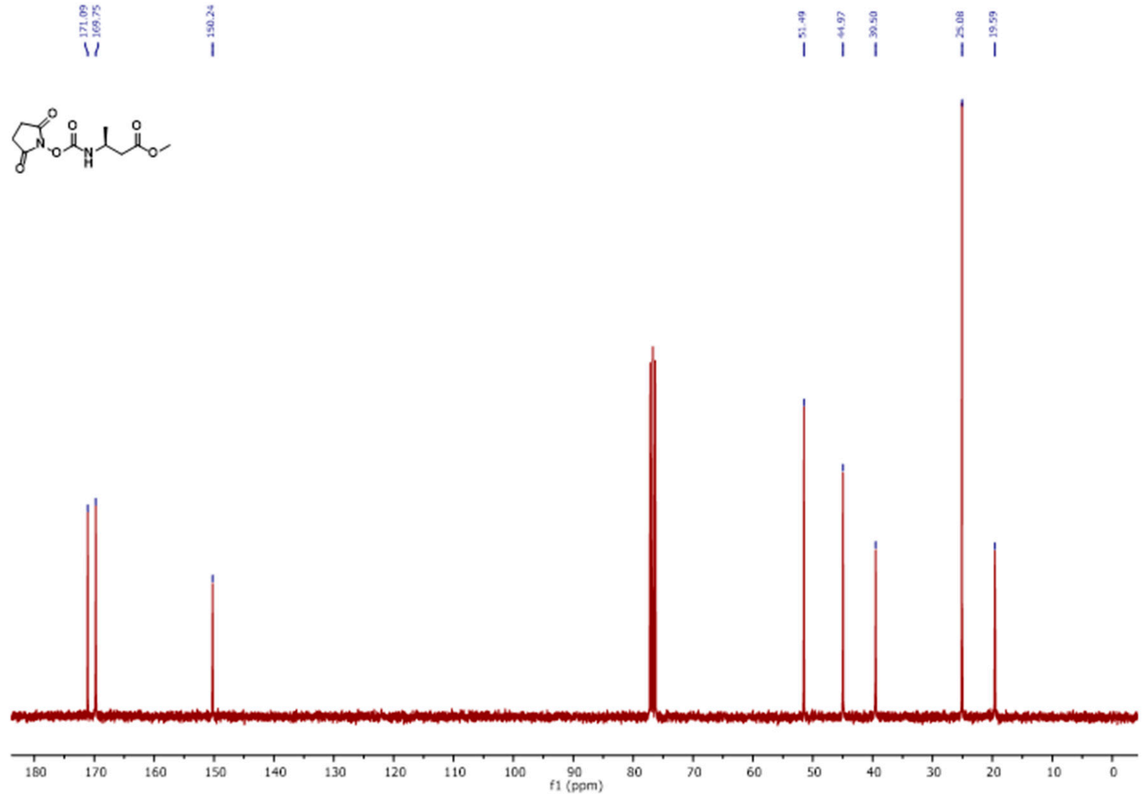

Figure S 17. ^{13}C NMR spectrum of **BB3g** in $CDCl_3$ (300 MHz)

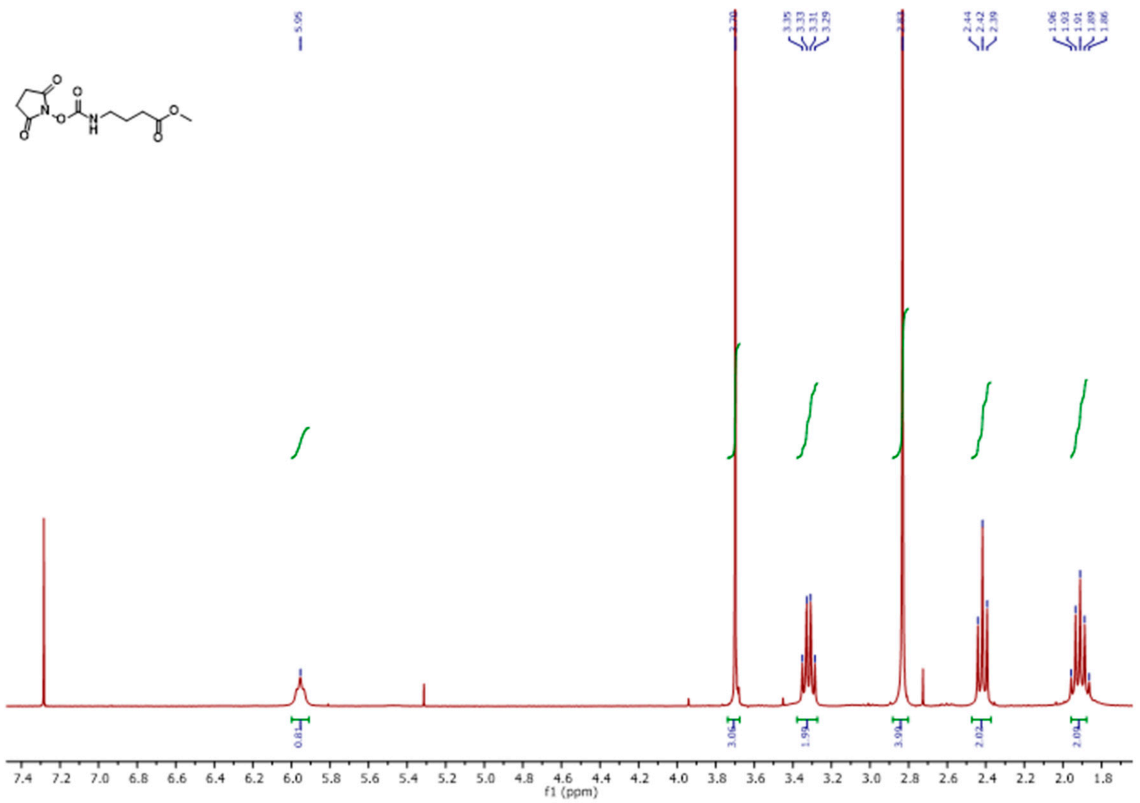

Figure S 18. ^{1}H NMR spectrum of **BB4h** in $CDCl_3$ (300 MHz)

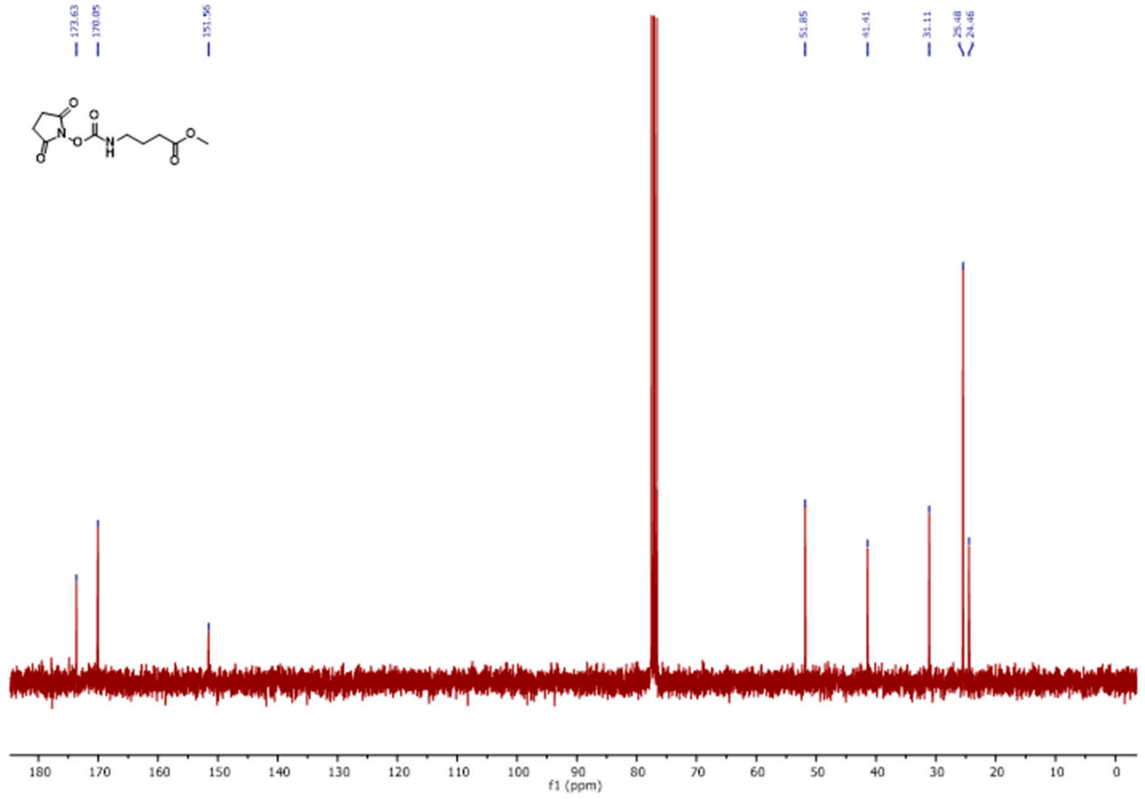

Figure S 19. ^{13}C NMR spectrum of **BB4h** in $CDCl_3$ (300 MHz)

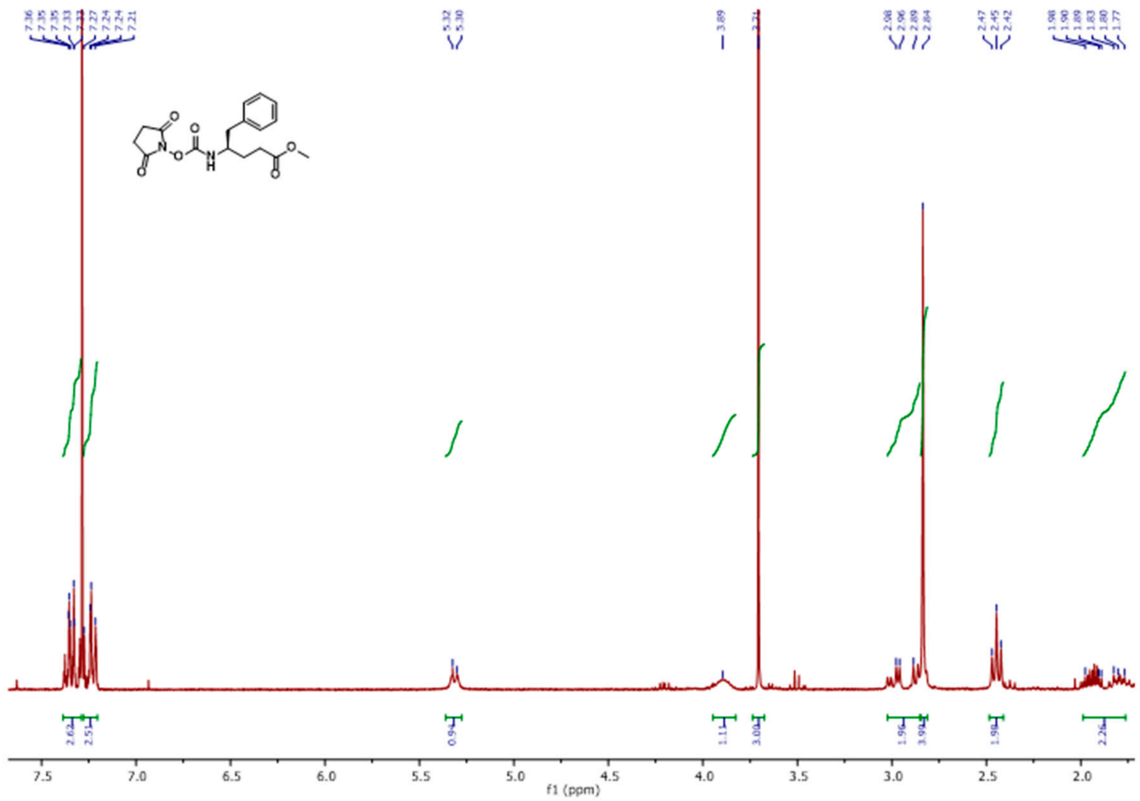

Figure S 20. ^{1}H NMR spectrum of **BB4i** in $CDCl_3$ (300 MHz)

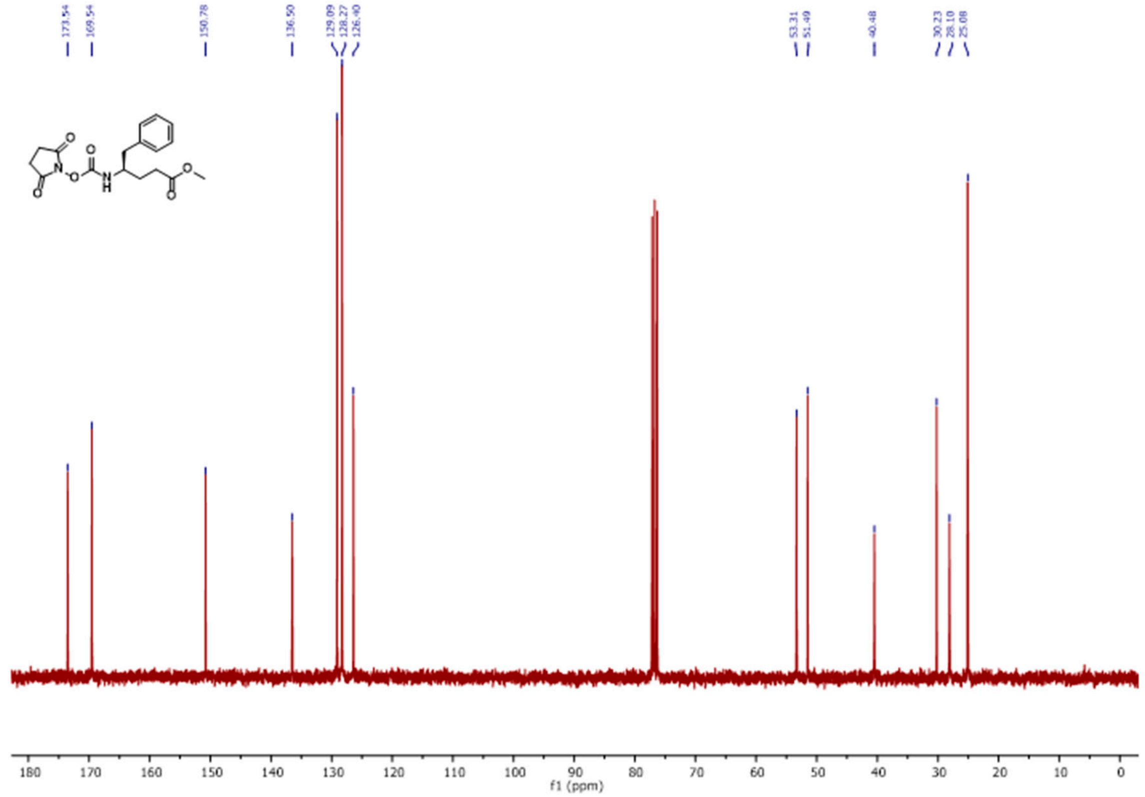

Figure S 21. ^{13}C NMR spectrum of **BB4i** in $CDCl_3$ (300 MHz)

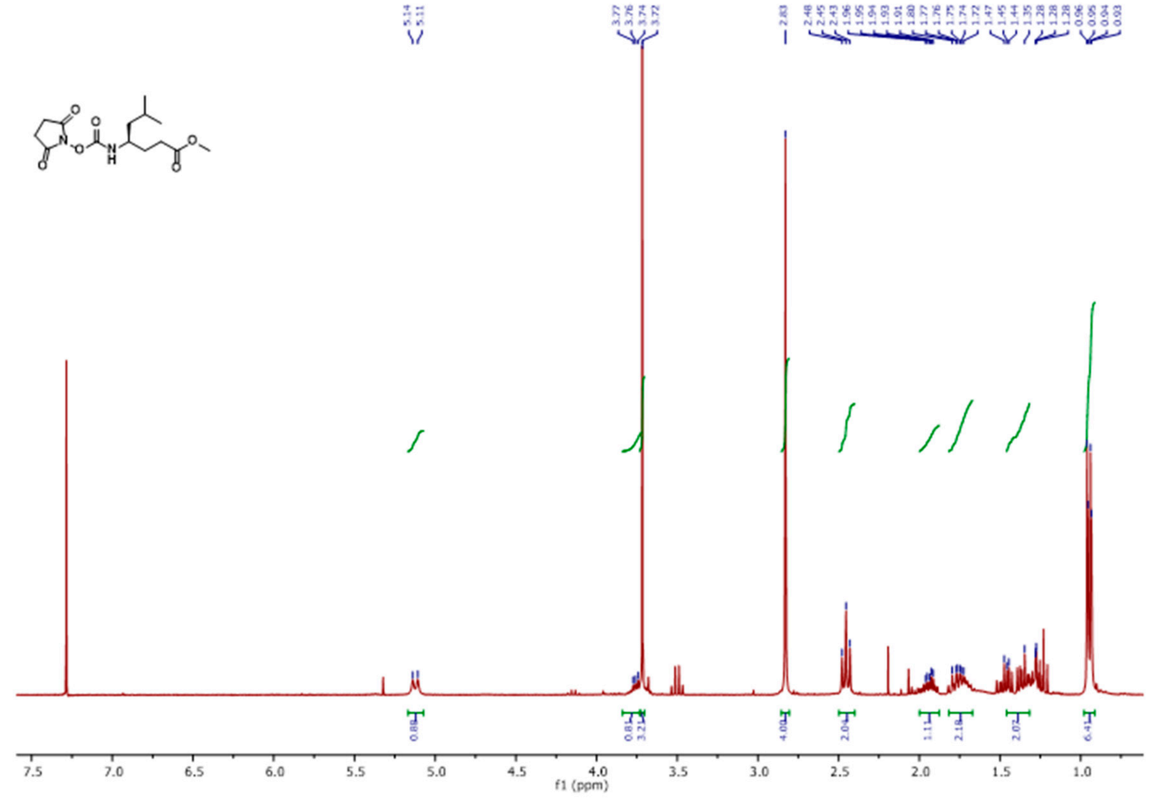

Figure S 22. ^{1}H NMR spectrum of **BB4j** in $CDCl_3$ (300 MHz)

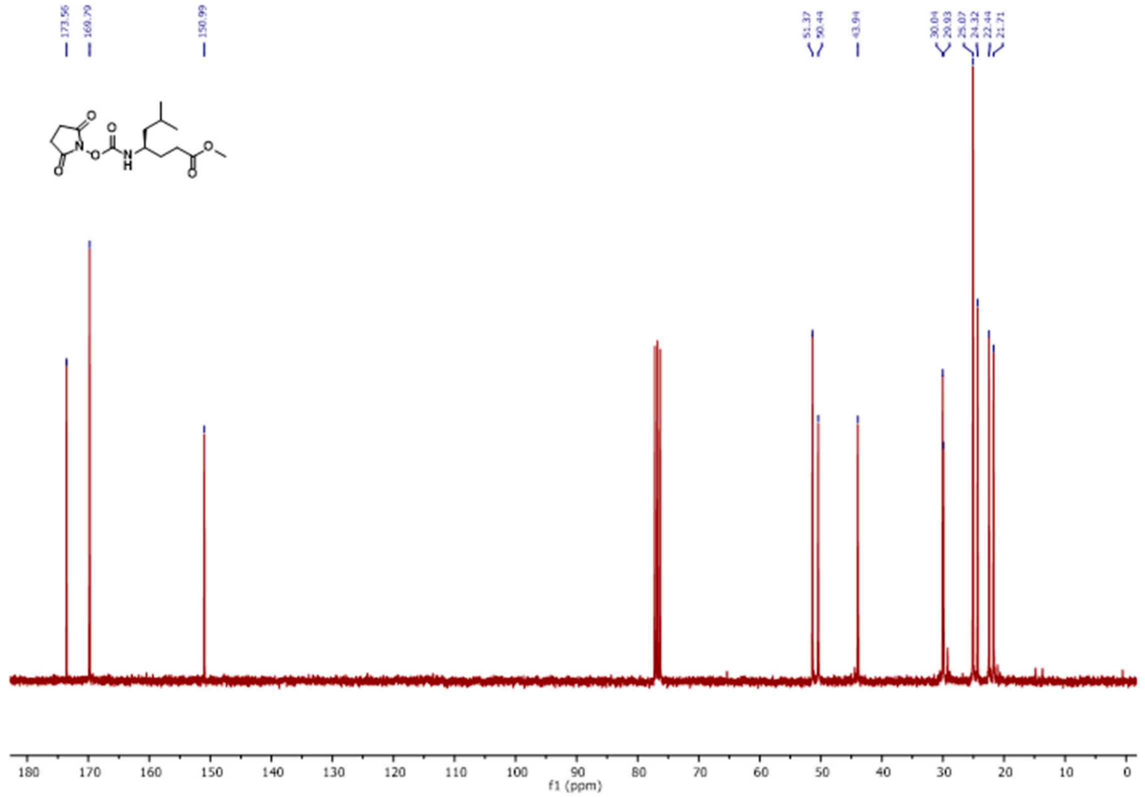

Figure S 23. ^{13}C NMR spectrum of **BB4j** in $CDCl_3$ (300 MHz)

3. NMR spectra of oligourea-esters of type 5

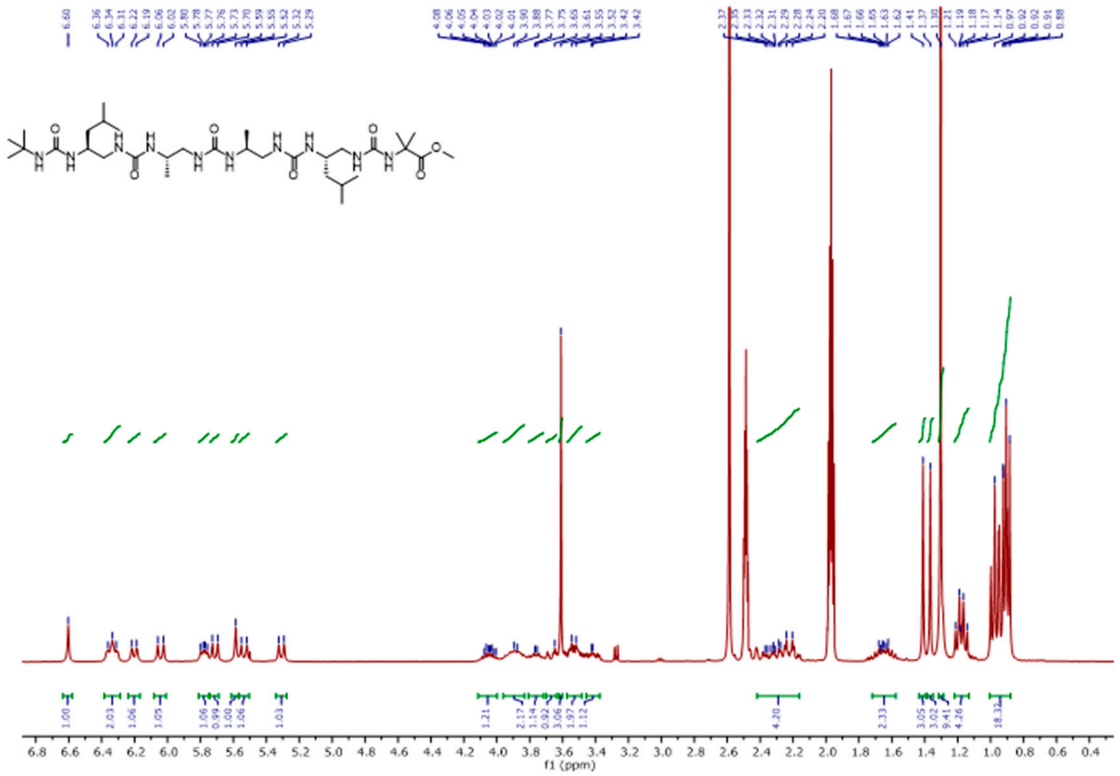

Figure S 24. ^{1}H NMR spectrum of **5a** in CD_3CN + 10% DMSO-d6 (300 MHz)

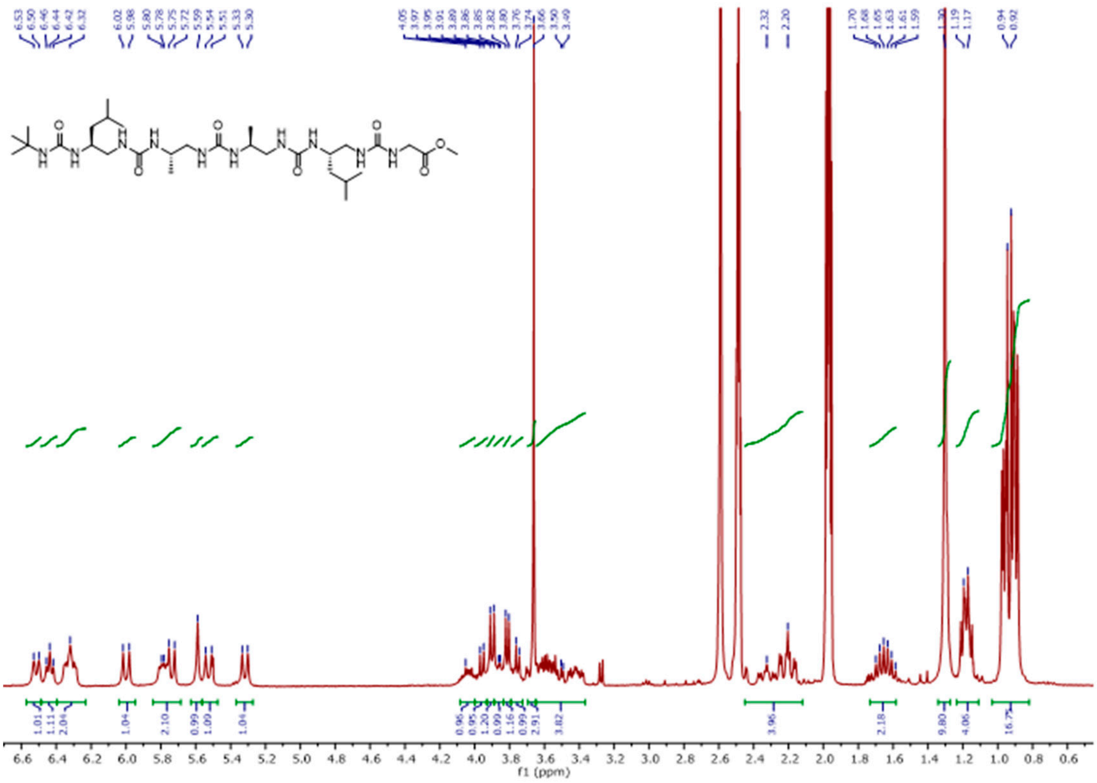

Figure S 25. ^{1}H NMR spectrum of **5b** in CD_3CN + 10% DMOS-d6 (300 MHz)

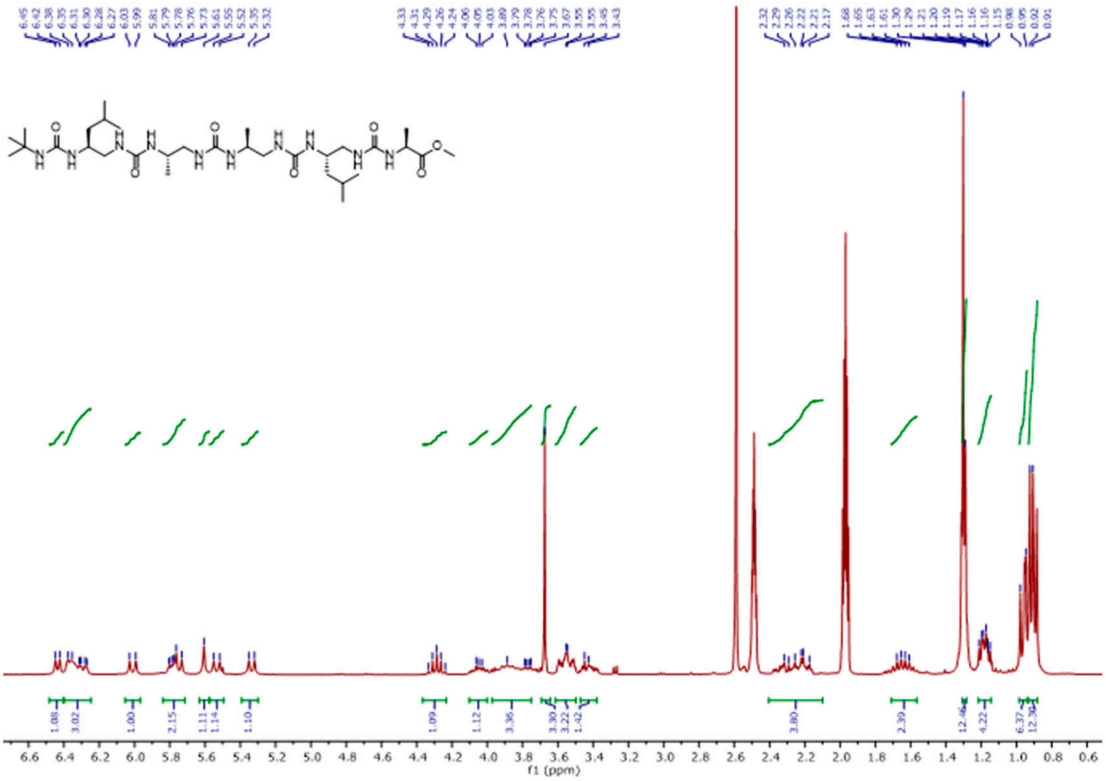

Figure S 26. ^{1}H NMR spectrum of **5c** in CD_3CN + 10% DMSO-d6 (300 MHz)

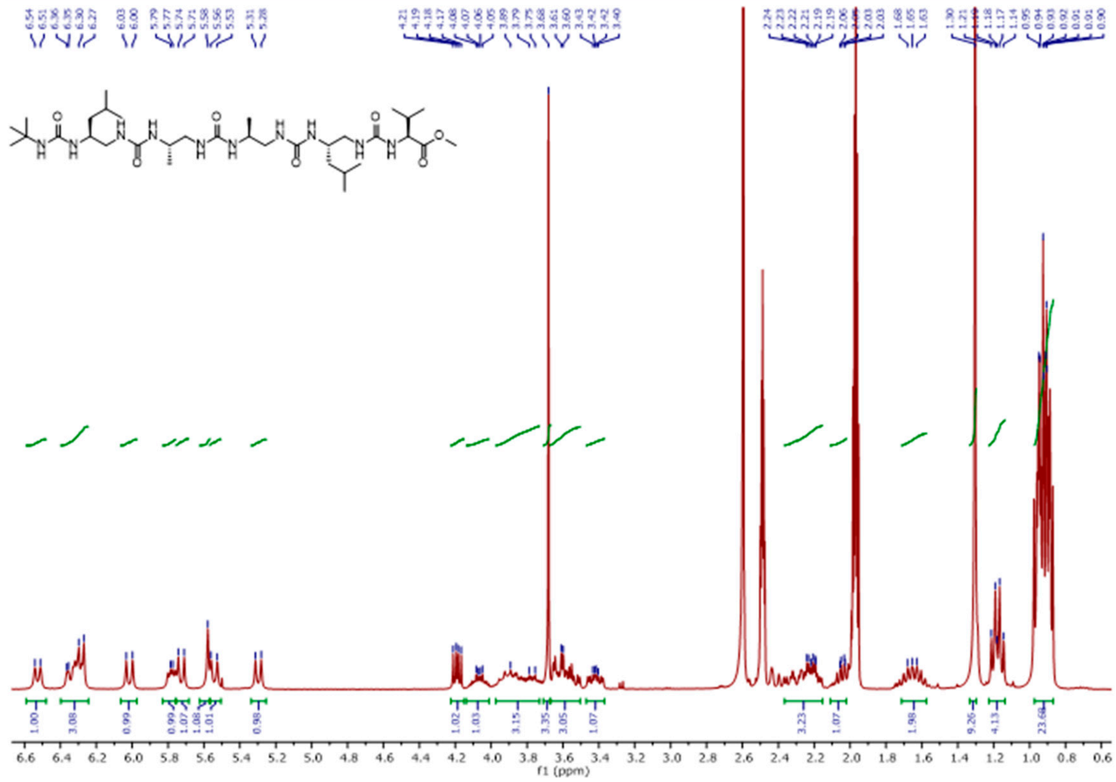

Figure S 27. ^{1}H NMR spectrum of **5d** in CD_3CN + DMSO-d6 (300 MHz)

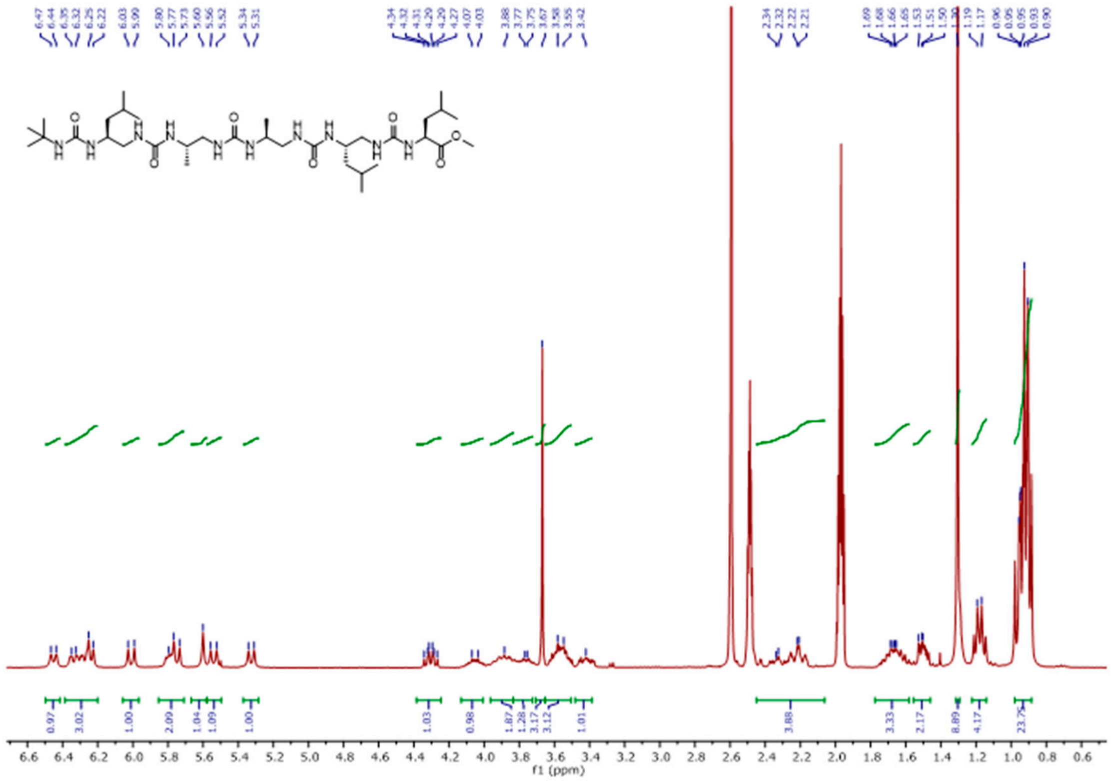

Figure S 28. ^{1}H NMR spectrum of **5e** in CD_3CN + 10% DMSO-d6 (300 MHz)

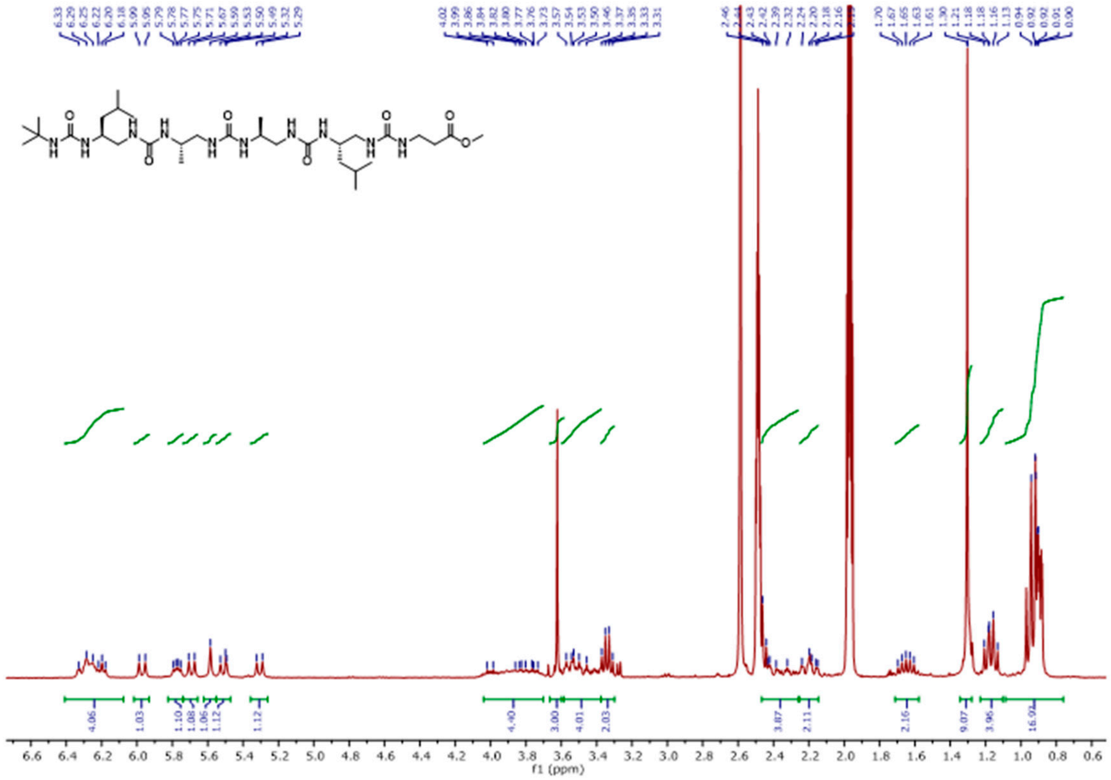

Figure S 29. ^{1}H NMR spectrum of **5f** in CD_3CN + 10% DMSO-d6 (300 MHz)

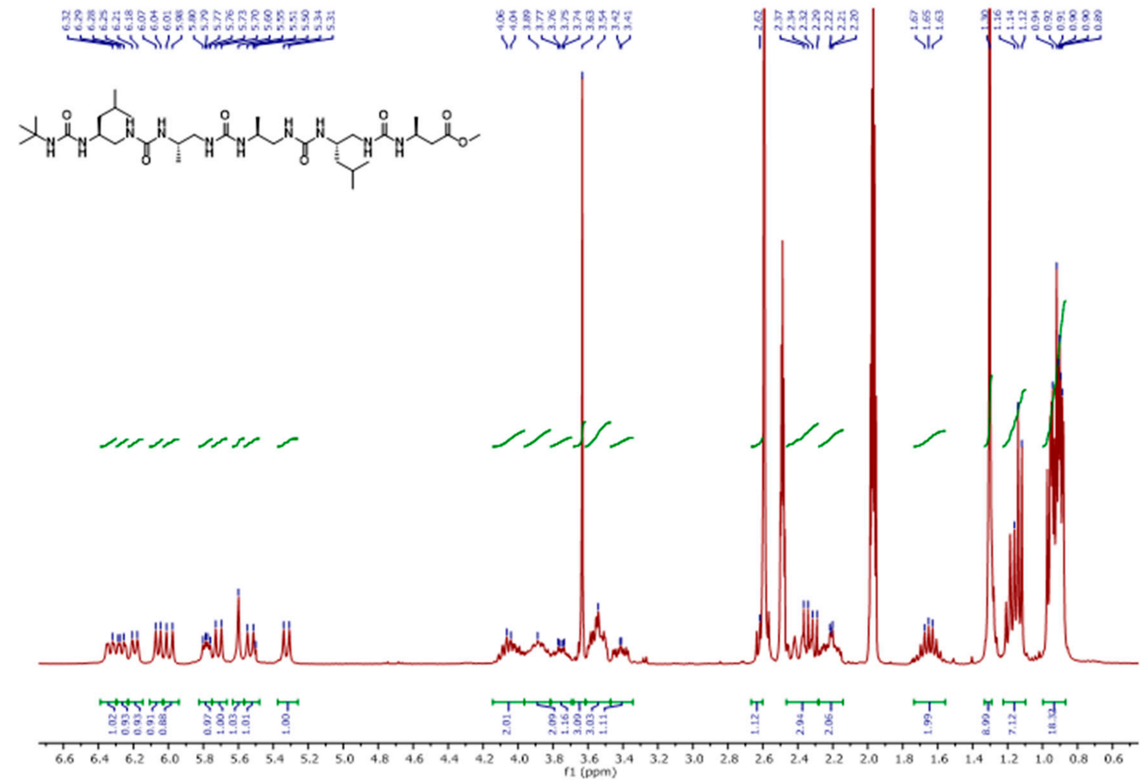

Figure S 30. ^{1}H NMR spectrum of **5g** in CD_3CN + 10% DMSO-d6 (300 MHz)

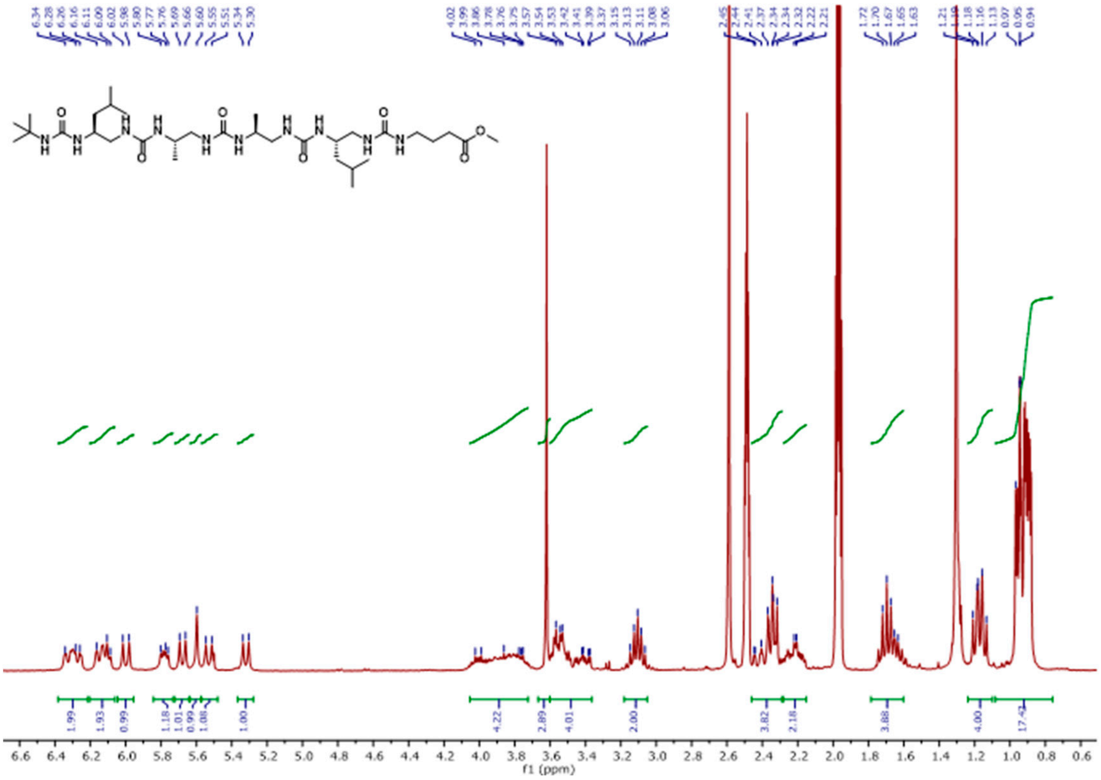

Figure S 31. ^{1}H NMR spectrum of **5h** in CD_3CN + 10% DMSO-d6 (300 MHz)

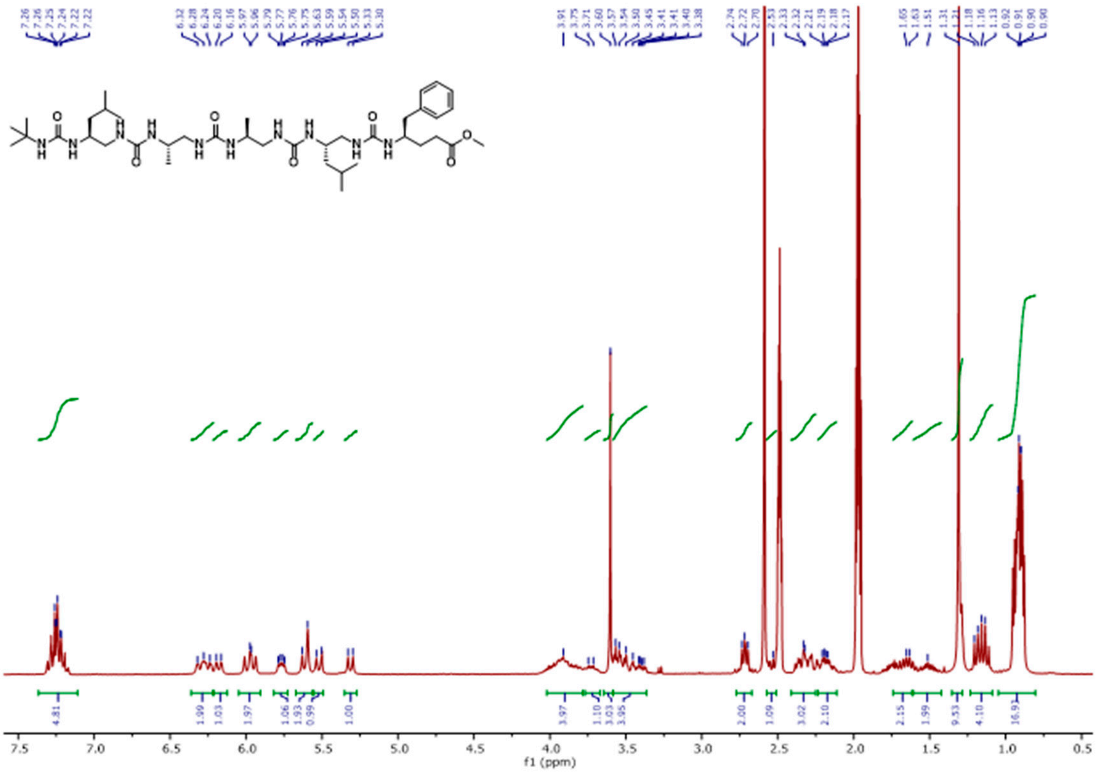

Figure S 32. ^{1}H NMR spectrum of **5i** in CD_3CN + 10% DMSO-d6 (300 MHz)

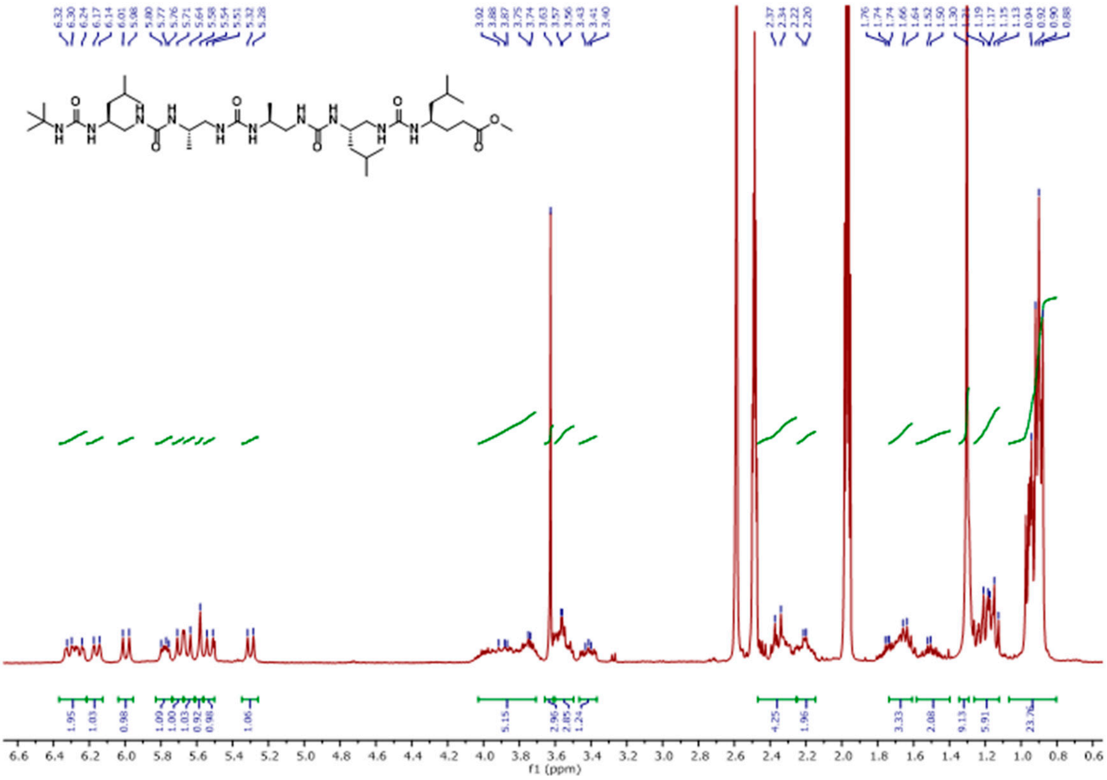

Figure S 33. ^{1}H NMR spectrum of **5j** in CD_3CN + 10% DMSO-d6 (300 MHz)

4. NMR spectra of oligourea-acids 1.

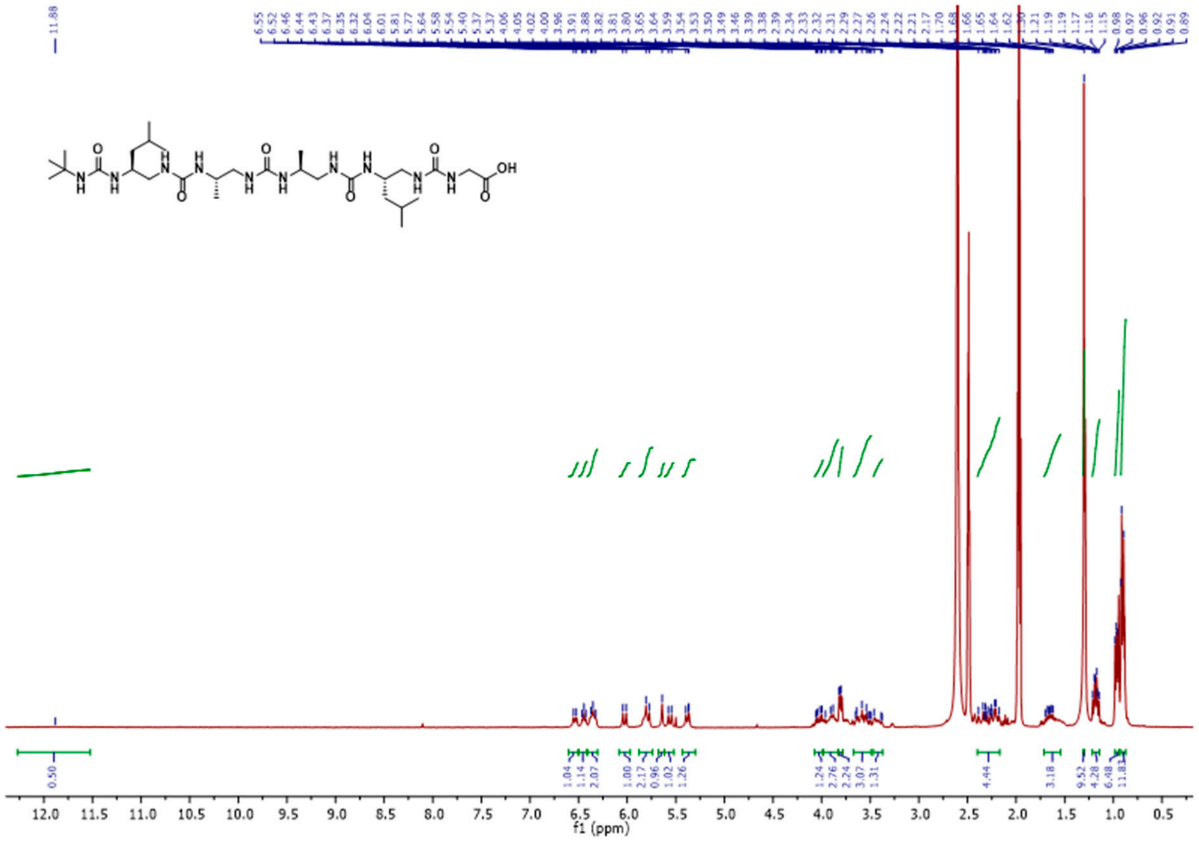

Figure S 34. ^{1}H NMR spectrum of **1b** in CD_3CN + 10% DMSO-d6 (300 MHz)

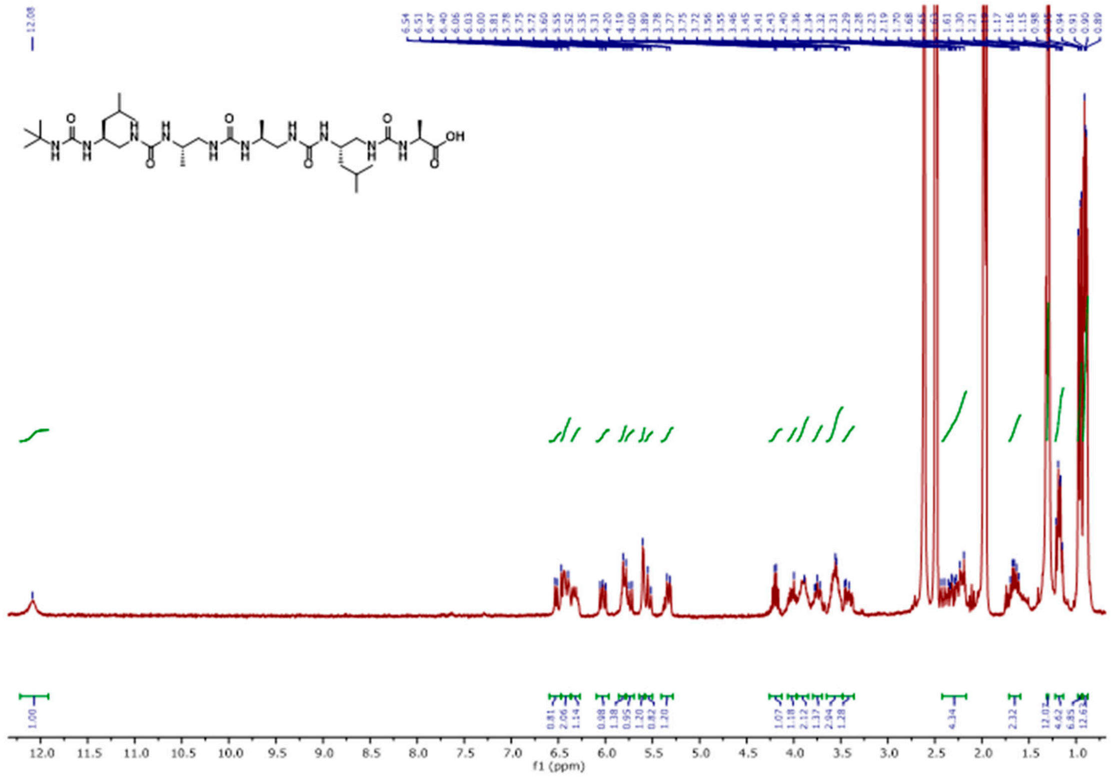

Figure S 35. ^{1}H NMR spectrum of **1c** in CD_3CN + 10% DMSO-d6 (300 MHz)

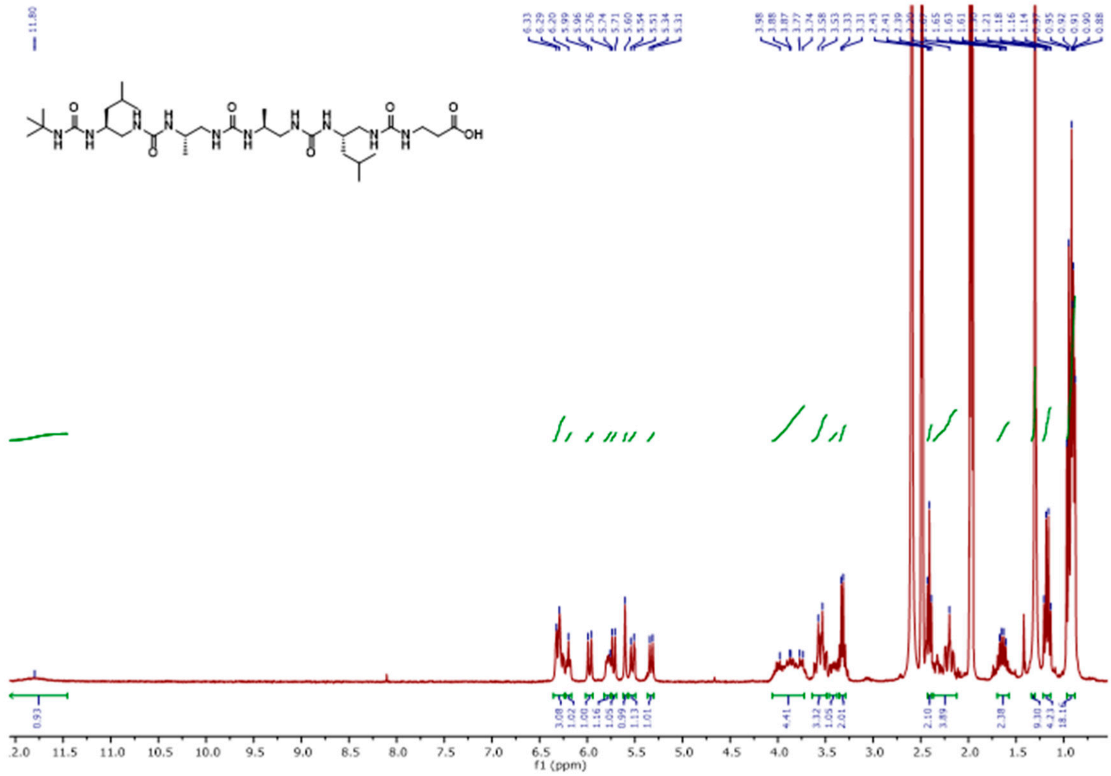

Figure S 36. ^1H NMR spectrum of **1f** in CD_3CN + 10% DMSO-d6 (300 MHz)

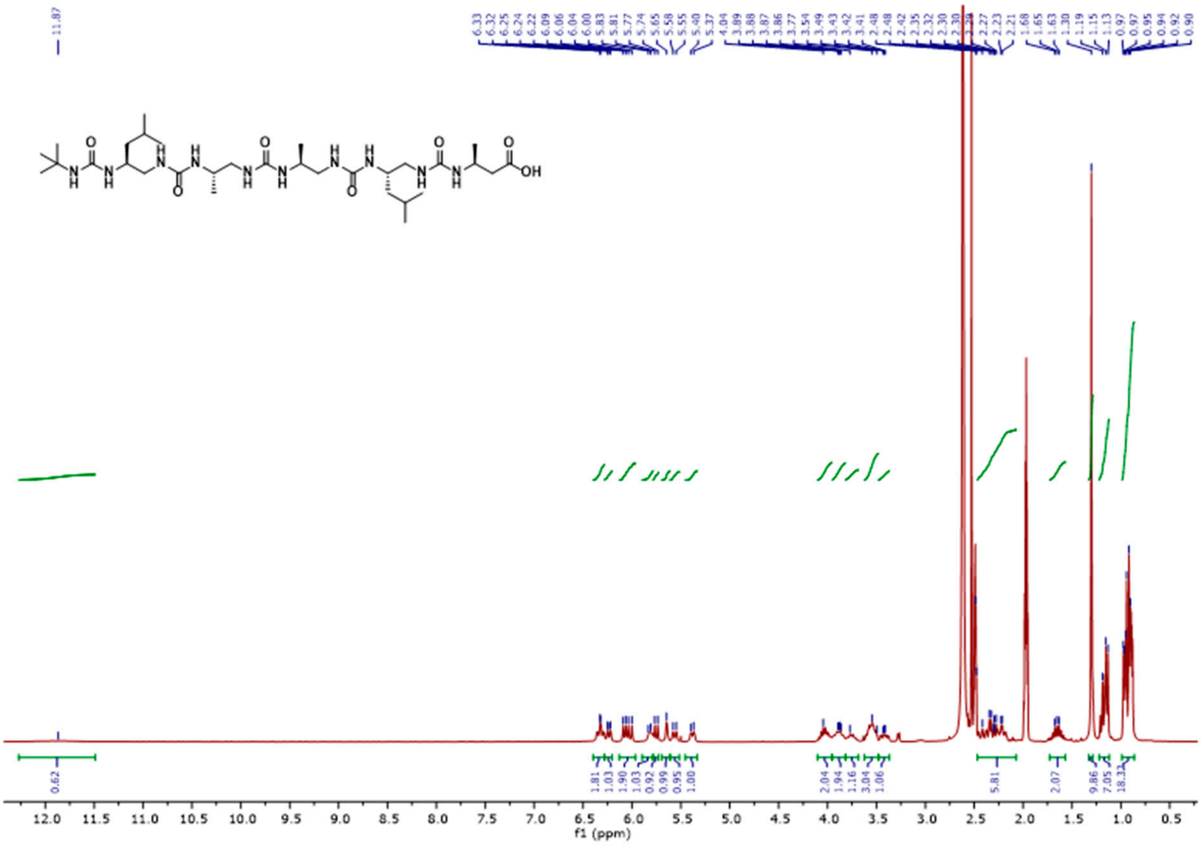

Figure S 37. ^1H NMR spectrum of **1g** in CD_3CN + 10% DMSO-d6 (300 MHz)

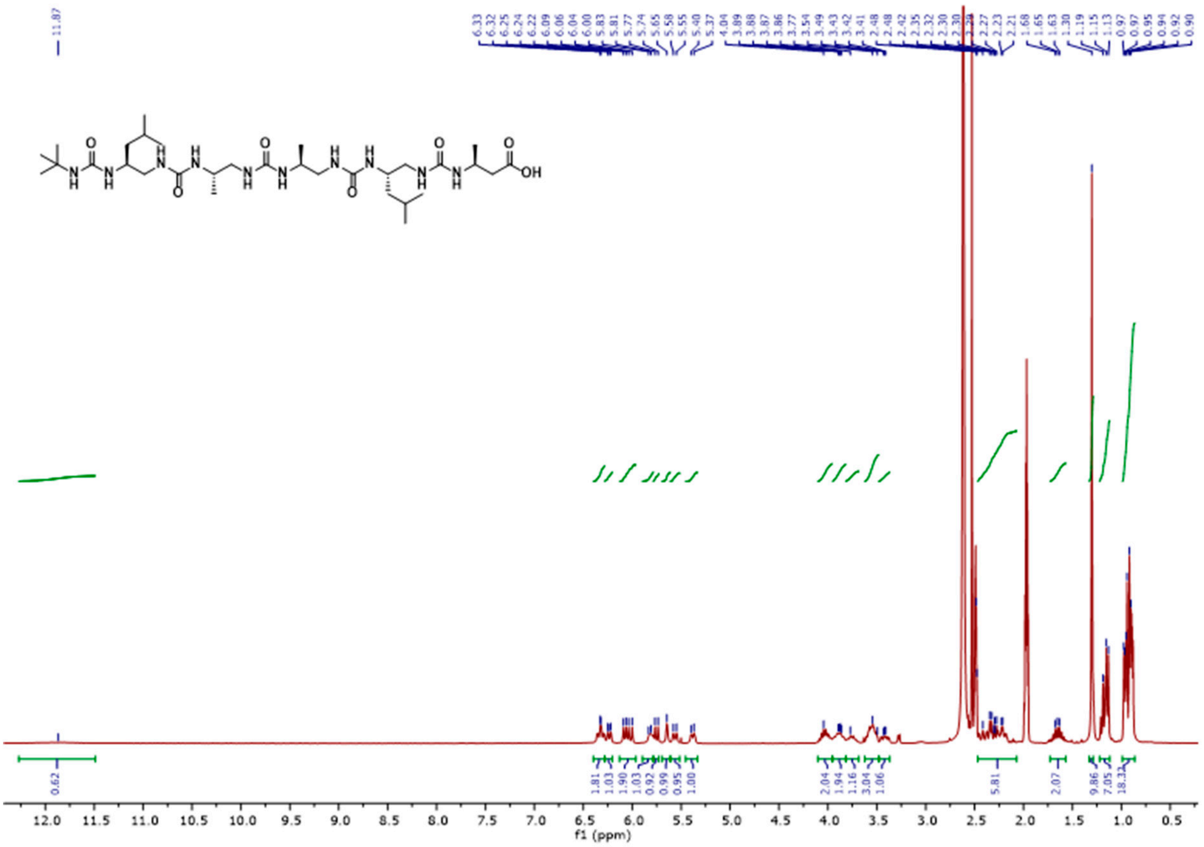

Figure S 38. ^{1}H NMR spectrum of **1h** in CD_3CN + 10% DMSO-d6 (300 MHz)

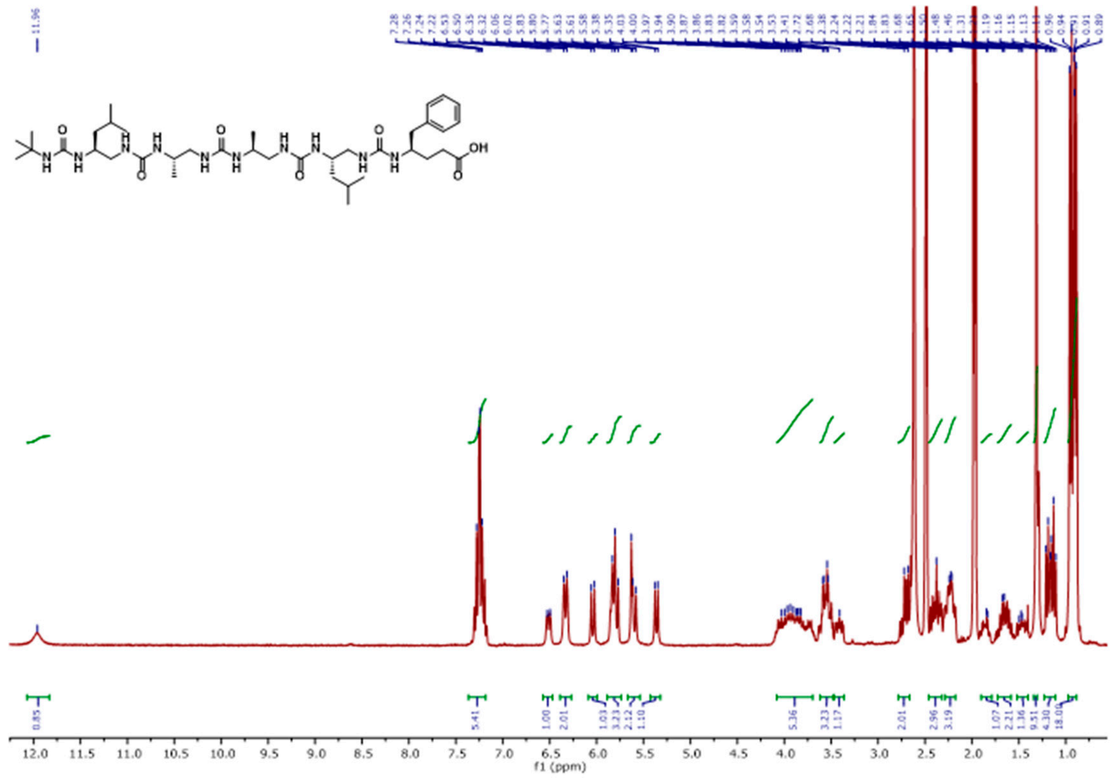

Figure S 39. ^{1}H NMR spectrum of **1i** in CD_3CN + 10% DMSO-d6 (300 MHz)

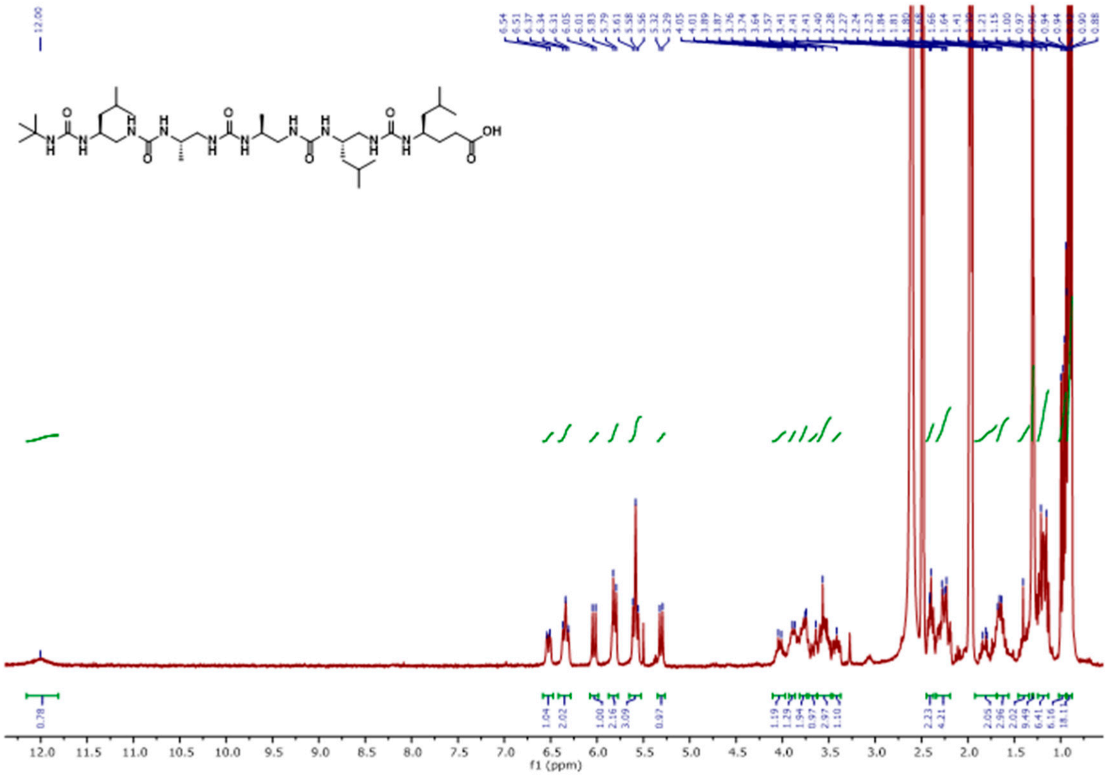

Figure S 40. ^{1}H NMR spectrum of **1j** in CD_3CN + 10% DMSO-d6 (300 MHz)

5. NMR spectra of oligourea-hydantoin/dihydrouracil 2.

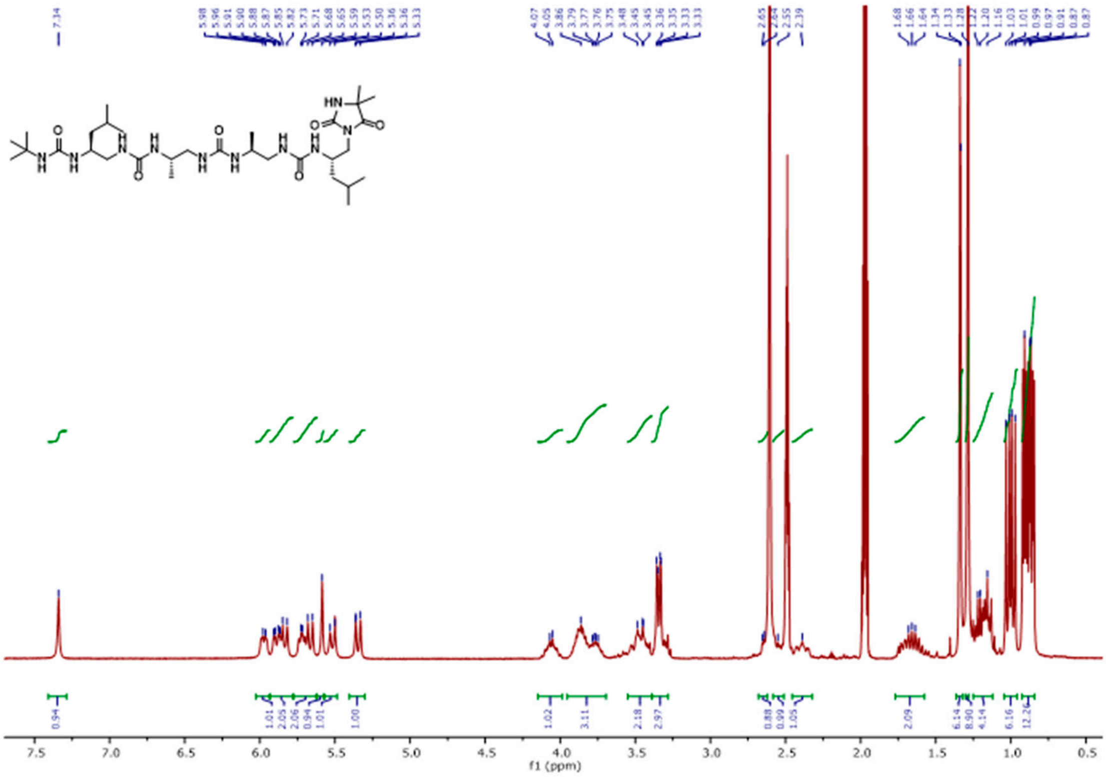

Figure S 41. ^{1}H NMR spectrum of **2a** in CD_3CN + 10% DMSO-d6 (300 MHz)

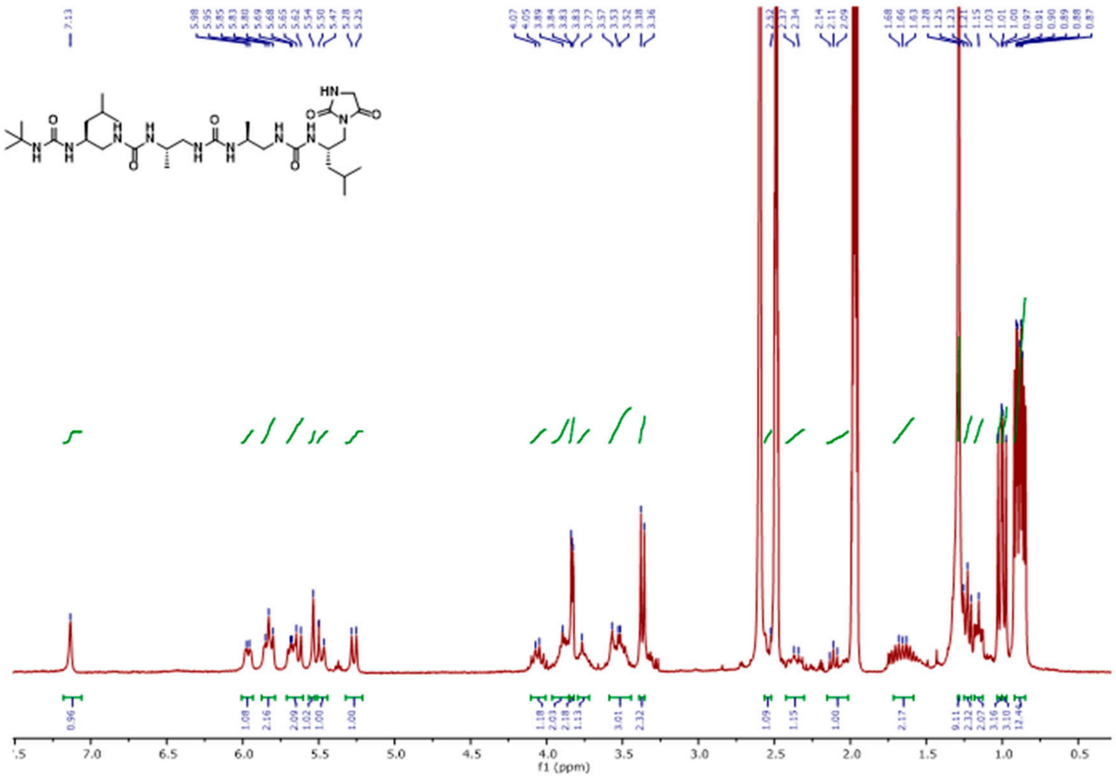

Figure S 42. ^{1}H NMR spectrum of **2b** in CD_3CN + 10% DMSO-d6 (300 MHz)

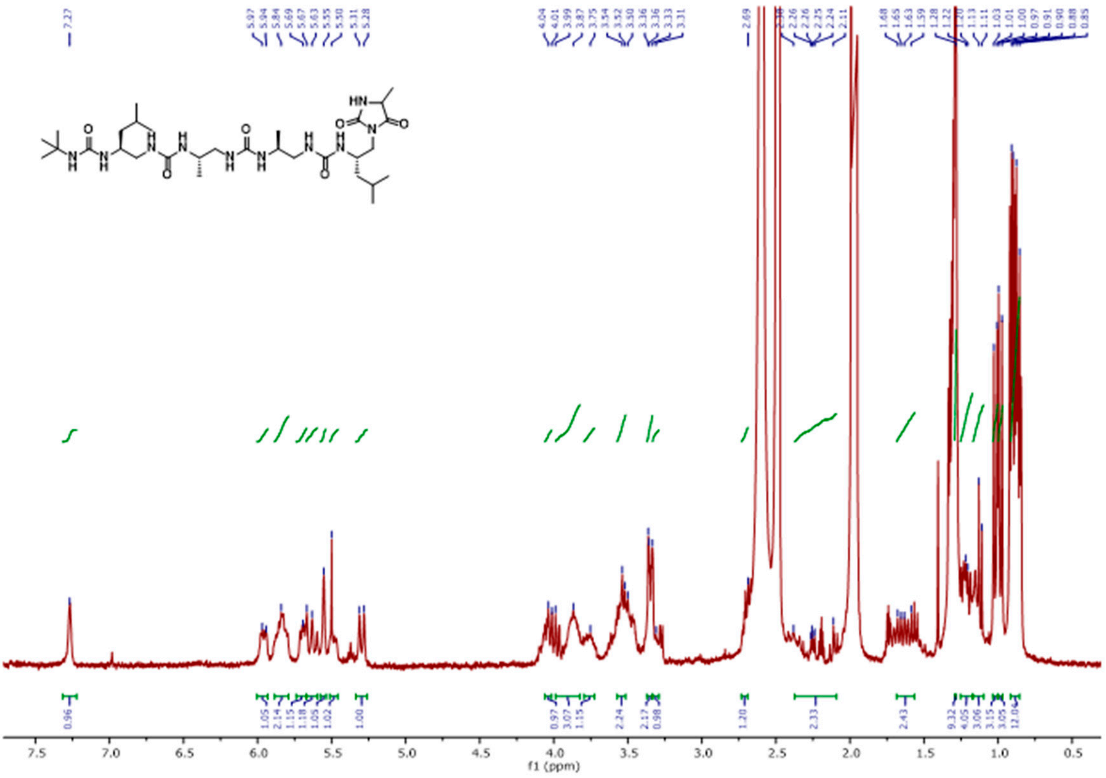

Figure S 43. ^{1}H NMR spectrum of **2c** in CD_3CN + 10% DMSO-d6 (300 MHz)

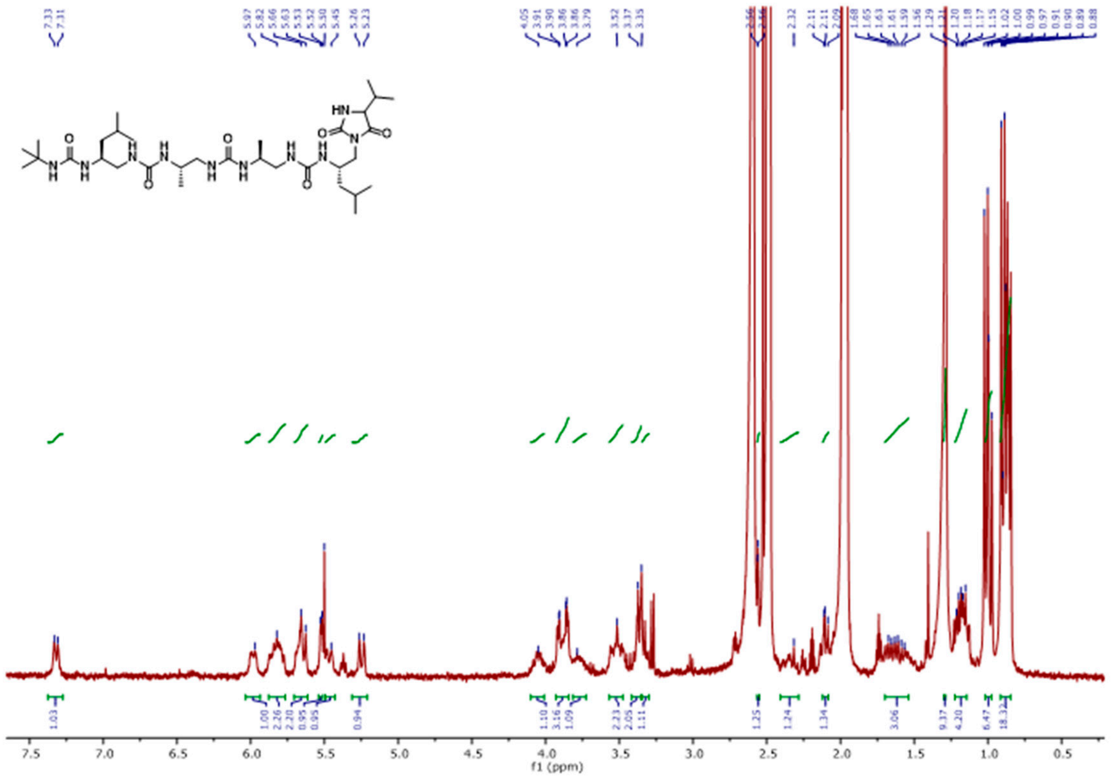

Figure S 44. ^{1}H NMR spectrum of **2d** in CD_3CN + 10% DMSO-d6 (300 MHz)

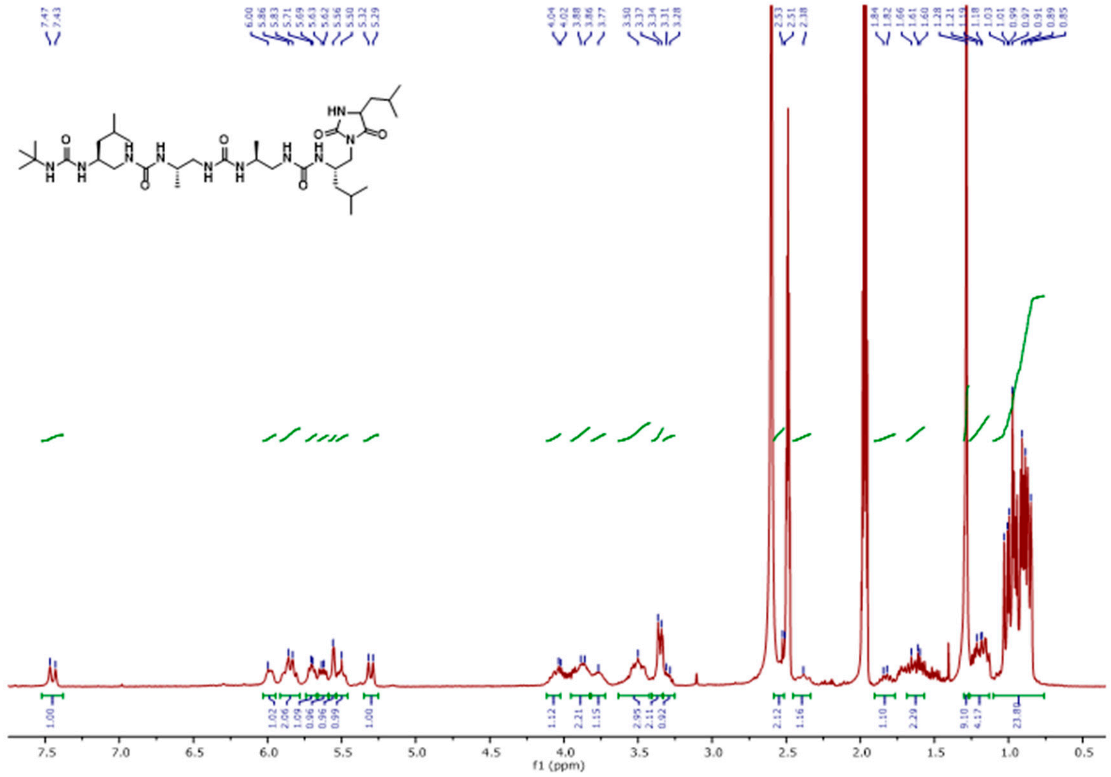

Figure S 45. ^{1}H NMR spectrum of **2e** in CD_3CN + 10% DMSO-d6 (300 MHz)

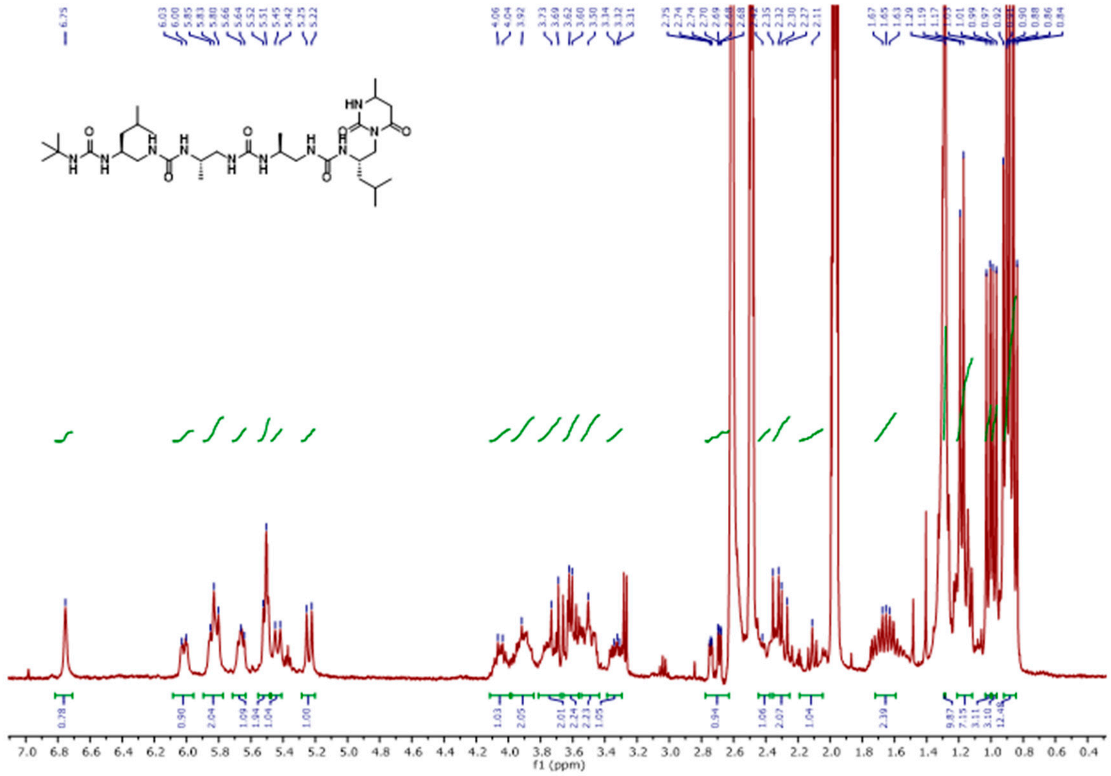

Figure S 46. ^{1}H NMR spectrum of **2g** in CD_3CN + 10% DMSO-d6 (300 MHz)

6. Signal assignments of oligourea-acids 1 and oligourea-hydantoin/dihydrouracil 2 based on 2D NMR spectra.

Experiments were recorded on a Bruker AVANCE 500 (500 MHz) spectrometer at 298K. Spin systems were assigned using COSY and TOCSY spectra. Sequential assignment was performed on the basis of N'H(*i*+1) / NH(*i*) ROESY correlations.

Table S 1. Signal assignments of oligoureas with hydantoin (**2a-e**) or dihydrouracil (**2g**) residues at C-terminus ased on 2D NMR spectra (doubling of signals resulting from epimerization was marked only for urea/hydantoin protons)

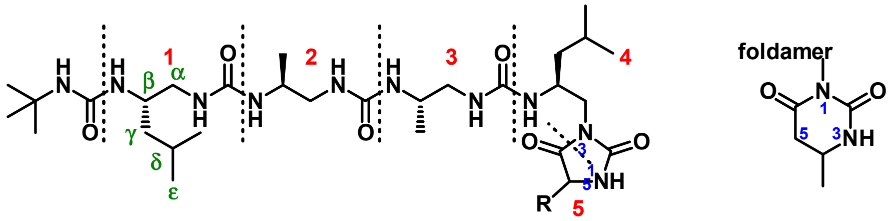

Res	Cmpd	HN	HN'	$^{\alpha}CH^{1}$	$^{\alpha}CH^{2}$	$^{\beta}CH$	$^{\gamma}CH$	$^{\delta}CH$	$^{\varepsilon}CH$	CH_3 (tBu)
tBuNH	2a*	-	5.52 (s)	-	-	-	-	-	-	1.26
	2b	-	5.51 (s)	-	-	-	-	-	-	1.26
	2c	-	5.53 (s)	-	-	-	-	-	-	1.26
	2d	-	5.53<br>5.54<br>*(diastereomers)*	-	-	-	-	-	-	1.26

	**2e**[$]	-	5.46<br>5.47<br>*(diastereomers*)	-	-	-	-	-	-	1.26
	**2g**	-	5.48<br>(overlapped with NHLeu$_4$U)	-	-	-	-	-	-	1.26
**Leu1u**	**2a**	5.27<br>(d, *J* = 9.4 Hz)	5.66<br>(dd, *J* = 7.3, 4.7 Hz)	3.30	2.47	3.84	1.13	1.62	0.86	-
	**2b**	5.24<br>(d, *J* = 9.4 Hz)	5.65<br>(dd, *J* = 7.6, 4.5 Hz)	3.32	2.44	3.83	1.12	1.61	0.86	-
	**2c**	5.28<br>(d, *J* = 9.5 Hz)	5.67<br>(dd, *J* = 8.5, 5.0 Hz)	3.31	2.46	3.84	1.13	1.69	0.84/<br>0.87	-
	**2d**	5.27<br>(d, *J* = 9.5 Hz)	5.68<br>(broad signal)	3.31	2.45	3.85	1.12/<br>1.26	1.60	0.85	-
	**2e**	5.21<br>(d, *J* = 9.4 Hz)<br>5.20<br>(d, *J* = 9.3 Hz)<br>(*diastereomers)*	5.64<br>(m, overlapped with NHLeu$_4$u)	3.31	2.49	3.85	1.15	1.60	0.85	-
	**2g**	5.21<br>(d, *J* = 9.3 Hz)	5.63<br>(dd, *J* = 7.5, 4.8 Hz)	3.30	2.42	3.87	1.11	1.61	0.85	-
**Ala2u**	**2a**	5.46<br>(d, *J* = 8.6 Hz)	5.84<br>(*pseudo*-d, *J* = 7.0 Hz)	3.47	2.35	3.84	0.99	-	-	-
	**2b**	5.45<br>(d, *J* = 8.7 Hz)	5.82<br>(overlapped with NH Ala$_3$U)	3.49	2.33	3.86	0.99	-	-	-
	**2c**	5.47<br>(broad signal, overlapped with DCM)	5.84<br>(overlapped with NH Ala$_3$U)	3.49	2.36	3.86	0.99	-	-	-
	**2d**	5.46<br>(broad signal, overlapped with DCM)	5.85<br>(overlapped with NH Ala$_3$U)	3.48	2.32	3.86	0.99	-	-	-
	**2e**	5.41<br>(broad signal)	5.88<br>(overlapped with NH Ala$_3$U)	3.49	2.36	3.89	1.00	-	-	-
	**2g**	5.40<br>(d, *J* = 9.5 Hz)	5.81<br>(overlapped with NH Ala$_3$U)	3.48	2.31	3.91	0.99	-	-	-
**Ala3u**	**2a**	5.79<br>(d, *J* = 8.5 Hz)	5.93<br>(*pseudo*-d, *J* = 5.5 Hz)	3.43	2.58	3.74	0.95	-	-	-
	**2b**	5.79<br>(overlapped with N'H Ala$_2$U)	5.94<br>(pseudo-d, J = 6.2 Hz)	3.50	2.54	3.73	0.95	-	-	-
	**2c**	5.80<br>(overlapped with N'H Ala$_2$U)	5.94<br>(*pseudo*-d, *J* = 6.5 Hz)	3.46	2.56	3.74	0.97	-	-	-
	**2d**	5.79<br>(overlapped with N'H Ala$_2$U)	5.96<br>(*pseudo*-d, *J* = 5.9 Hz)	3.48	2.53	3.74	0.96	-	-	-
	**2e**	5.83<br>(overlapped with N'H Ala$_2$U)	5.97<br>(broad signal)	3.49	2.54	3.77	0.97	-	-	-

	**2g**	5.79<br>(overlapped with N’H Ala$_2$U)	5.99<br>(*pseudo*-d, *J* = 6.4 Hz)	3.49	2.54	3.73	0.95	-	-	-
**Leu4u**	**2a**	5.62<br>(d, *J* = 9.3 Hz)	-	3.32	-	4.04	1.17	1.68	0.86	-
	**2b**	5.61<br>(d, J = 9.3 Hz)	-	3.34	-	4.03	1.20	1.66	0.86	-
	**2c**	5.59<br>(d, *J* = 9.5 Hz)<br>5.63<br>(d, *J* = 9.3 Hz)<br>*(diastereomers)*	-	3.32	-	4.03	1.19	1.66	0.83/<br>0.88	-
	**2d**	5.63<br>(d, *J* = 8.9 Hz)	-	3.33	-	4.00	1.18	1.66	0.86	-
	**2e**	5.61<br>(m, overlapped with N’HLeu$_1$u)	-	3.34	-	4.03	1.21	1.68	0.85	-
	**2g**	5.48<br>(overlapped with NHtBu)	-	3.66	3.57	4.02	1.15	1.68	0.84	-

hydantoin
dihydrouracil ring

**Res**		**NH1**	**CH5**	**Substituents at C5**
**Aib$_{hyd}$**	**2a**	7.29 (s)	-	1.31/1.30 (CH3)
**Gly$_{hyd}$**	**2b**	7.11 (s)	3.80	-
**Ala$_{hyd}$**	**2c**	7.25 (s)	3.94<br>4.00<br>*(diastereomers)*	1.30/1.29 (CH_3, *diastereomers*)
**Val$_{hyd}$**	**2d**	7.31<br>7.29<br>*(doubled, diastereomers)*	3.83<br>3.88<br>*(diastereomers)*	2.08 (CH), 0.84 (CH_3)
**Leu$_{hyd}$**	**2e**	7.22<br>7.26<br>*(diastereomers)*	3.94<br>4.00<br>*(diastereomers)*	1.56/1.46 (CH_2), 1.80 (CH), 0.94 (CH_3)
**Ala$_{dihydrouracil}$**	**2g[@]**	6.73 (s)	3.58 (C4H)<br>2.27/2.68 (C5H)	1.16 (CH_3)

[*] 10% DMSO-d6 in CD_3CN
[$] CD_3CN
[@] different numbering of dihydrouracil (from *N*-foldamer) than the hydantoin ring

Table S 2. Signal assignments of oligoureas (**1b,c,f-j**) with amino acid residue at C-terminus based on 2D NMR spectra

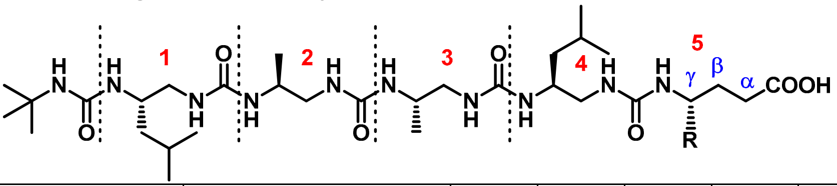

Res	Cmpd	HN	HN'	αCH[1]	αCH[2]	βCH	γCH	δCH	εCH	CH_3 (tBu)
**tBuNH**	**1b***	-	5.62 (s)	-	-	-	-	-	-	1.27
	**1c&**	-	5.58 (s)<br>5.57 (s)	-	-	-	-	-	-	1.27
	**1f**	-	5.58 (s)	-	-	-	-	-	-	1.27
	**1g**	-	5.64 (s)	-	-	-	-	-	-	1.27
	**1h**	-	5.57 (s)	-	-	-	-	-	-	1.27
	**1i**	-	5.60 (s)	-	-	-	-	-	-	1.28
	**1j**	-	5.55 (s)	-	-	-	-	-	-	1.27
**Leu1u**	**1b**	5.37<br>(d, *J* = 10.0 Hz)	5.80<br>(dd, *J* = 7.6, 4.0 Hz)	3.40	2.31	3.89	1.16	1.65	0.89	-
	**1c**	5.29<br>(d, *J* = 9.5 Hz)<br>5.30<br>(d, *J* = 9.4 Hz)	5.76<br>5.78<br>(overlapped with NHLeu$_4$U)	3.40	2.31	3.90	1.15	1.64	0.87<br>0.91	-
	**1f**	5.30<br>(d, *J* = 9.6 Hz)	5.75<br>(dd, *J* = 8.2, 4.3 Hz)	3.40	2.29	3.89	1.16	1.64	0.87	-
	**1g**	5.38<br>(d, *J* = 9.6 Hz)	5.80<br>(dd, *J* = 8.1, 4.4 Hz)	3.39	2.32	3.88	1.17	1.64	0.88	-
	**1h**	5.30<br>(d, *J* = 9.6 Hz)	5.79<br>(dd, *J* = 8.1, 4.4 Hz)	3.39	2.31	3.88	1.17	1.64	0.88	-
	**1i**	5.33<br>(d, *J* = 9.5 Hz)	5.80<br>(overlapped with NH γ-Phe)	3.38	2.31	3.88	1.16	1.66	0.85	-
	**1j**	5.28<br>(d, *J* = 9.5 Hz)	5.79<br>(overlapped with NHLeu$_4$U)	3.40	2.29	3.88	1.15	1.64	0.89	-
**Ala2u**	**1b**	5.54<br>(d, *J* = 10.2 Hz)	6.31<br>(overlapped with N'H Ala$_3$U)	3.61	2.18	3.75	0.95	-	-	-
	**1c**	5.50<br>(d, *J* = 9.8 Hz)<br>5.54<br>(d, *J* = 10.5 Hz)	6.30/6.33<br>(overlapped with N'H Ala$_3$U)	3.67<br>3.54	2.16<br>2.20	3.73	0.94	-	-	-
	**1f**	5.50<br>(d, *J* = 10.0 Hz)	6.28<br>(overlapped with N'H Leu$_4$U)	3.51	2.18	3.72	0.92	-	-	-
	**1g**	5.56<br>(d, *J* = 10.1 Hz)	6.31<br>(dd, *J* = 10.0, 2.6 Hz)	3.55	2.20	3.73	0.93	-	-	-

	**1h**	5.54<br>(d, *J* = 10.1 Hz)	6.30<br>(dd, *J* = 10.0, 2.7 Hz)	3.51	2.20	3.75	0.93	-	-	-
	**1i**	5.56<br>(d, *J* = 10.1 Hz)	6.30<br>(overlapped with N’HLeu$_4$U)	3.52	2.20	3.71	0.95	-	-	-
	**1j**	5.54<br>(overlapped with NHtBu and NH γ-Leu)	6.29<br>(overlapped with N’HLeu$_4$U)	3.52	2.21	3.74	0.95	-	-	-
**Ala3u**	**1b**	6.00<br>(d, *J* = 10.5 Hz)	6.33<br>(overlapped with N’HAla$_2$U)	3.51	2.20	4.00	0.93	-	-	-
	**1c**	5.99<br>(d, *J* = 10.7 Hz)<br>6.01<br>(d, *J* = 10.8 Hz)	6.28<br>6.38<br>(overlapped with N’H Ala$_2$U and N’HLeu$_4$U)	3.52	2.18	3.99	0.95	-	-	-
	**1f**	5.95<br>(d, *J* = 10.5 Hz)	6.24<br>(dd, *J* = 10.0, 3.0 Hz)	3.51	2.18	3.97	0.92	-	-	-
	**1g**	5.99<br>(d, *J* = 10.5 Hz)	6.27<br>(dd, *J* = 9.8, 2.9 Hz)	3.52	2.20	3.98	0.93	-	-	-
	**1h**	5.99<br>(d, *J* = 10.5 Hz)	6.34<br>(dd, *J* = 9.7, 2.9 Hz)	3.51	2.20	4.00	0.93	-	-	-
	**1i**	6.00<br>(d, *J* = 10.5 Hz)	6.49<br>(dd, *J* = 9.1, 2.2 Hz)	3.51	2.22	4.01	0.92	-	-	-
	**1j**	6.00	6.50	3.51	2.22	4.00	0.92	-	-	-
**Leu4u**	**1b**	5.76<br>(d, *J* = 9.7 Hz)	6.52<br>(*pseudo-d, J* = 8.1 Hz)	3.56	2.39	3.86	1.14	1.62	0.89	-
	**1c**	5.71<br>(d, *J* = 9.8 Hz)<br>5.77<br>(overlapped with N’HLeu$_1$U)	6.39<br>6.42<br>(overlapped with N’HAla$_3$U and NH α-Ala)	3.51<br>3.55	2.38<br>2.41	3.86<br>3.87	1.13	1.61	0.86	-
	**1f**	5.70<br>(d, *J* = 9.7 Hz)	6.28<br>(overlapped with N’H Ala$_2$U)	3.55	2.40	3.82	1.13	1.62	0.88	-
	**1g**	5.72<br>(d, *J* = 9.8 Hz)	6.20<br>(*pseudo*-d, *J* = 8.1 Hz)	3.52	2.39	3.84	1.14	1.61	0.88	-
	**1h**	5.72<br>(d, *J* = 9.8 Hz)	6.25<br>(*pseudo*-d, *J* = 8.2 Hz)	3.57	2.36	3.83	1.14	1.61	0.87	-
	**1i**	5.76<br>(d, *J* = 10.3 Hz)	6.30<br>(overlapped with N’H Ala$_2$U)	3.57	2.19	3.80	1.11	1.60	0.88	-
	**1j**	5.78<br>(overlapped with N’HLeu$_1$U)	6.32<br>(overlapped with N’HAla$_2$U)	3.61	2.25	3.84	1.14	1.64	0.96	-

amino acid residue

**Res**	**Cmpd**	**NH**	$^{\alpha}$CH	$^{\beta}$CH	$^{\gamma}$CH	Side chain	OH
**Gly**	**1b**	6.41<br>(t, *J* = 5.6 Hz)	3.79	-	-	-	11.47<br>(broad signal)

**α-Ala**	**1c**	6.43<br>(d, *J* = 7.2 Hz, 1H)<br>6.50<br>(d, *J* = 6.8 Hz, 1H)	4.17	-	-	1.29 (CH_3)	12.06
**β-hGly**	**1f**	6.17<br>(t, *J* = 6.0 Hz)	2.38	3.29	-	-	11.81
**β³hAla**	**1g**	6.04<br>(d, *J* = 8.2 Hz, 1H)	2.28/2.51<br>(*diastereotopic*)	4.00	-	1.18<br>(CH_3)	11.85
**GABA**	**1h**	6.05<br>(t, *J* = 5.8 Hz)	2.28	1.62/1.70<br>(*diastereotopic*)	3.02/3.20<br>(*diastereotopic*)	-	in the backgr ound
**γ-Phe**	**1i**	5.79<br>(overlapped with N'HLeu$_1$U)	2.34/2.40<br>(*diastereotopic*)	1.44/1.83<br>(*diastereotopic*)	3.95	2.61/2.71 (CH_2, *diastereotopic*)<br>7.15-7.26 (Ph)	11.93
**γ-Leu**	**1j**	5.57<br>(overlapped with NHtBu and NHAla$_2$U)	2.37	1.34/1.80<br>(*diastereotopic*)	3.74	1.18 (CH_2)<br>1.63 (CH)<br>0.88 (CH_3)	11.99

*10% DMSO-d6 in CD_3CN

& doubling of signals resulting from epimerization of amino acid residue at C-terminus

7. Additional 1D and 2D NMR spectra

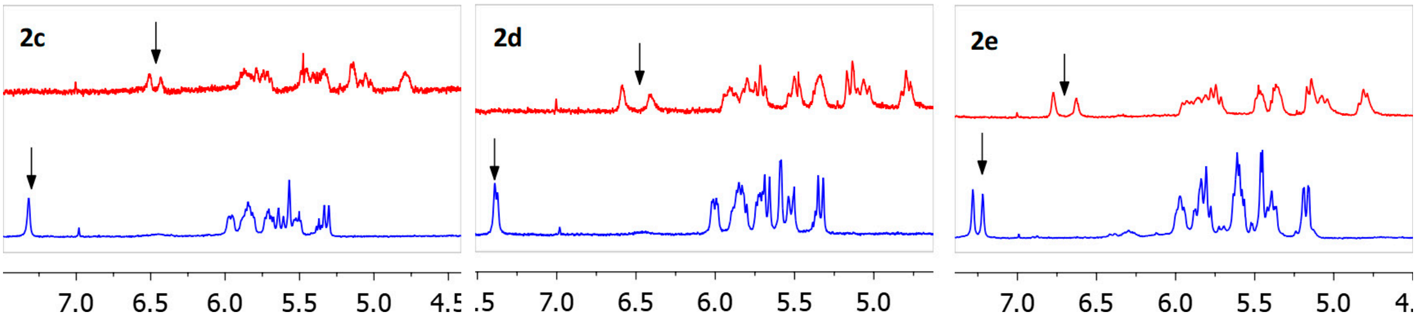

Figure S 47. Part of ^{1}H NMR spectra (300MHz) of oligourea-hydantoins **2c-2e** showing NH region. Red spectra were recorded in pure CD3CN, blue spectra were recorded in the mixture of DMSO-d_6 in CD_3CN. An arrow indicates hydantoin protons, doubled because of the epimerisation of the C-terminus amino acid residues

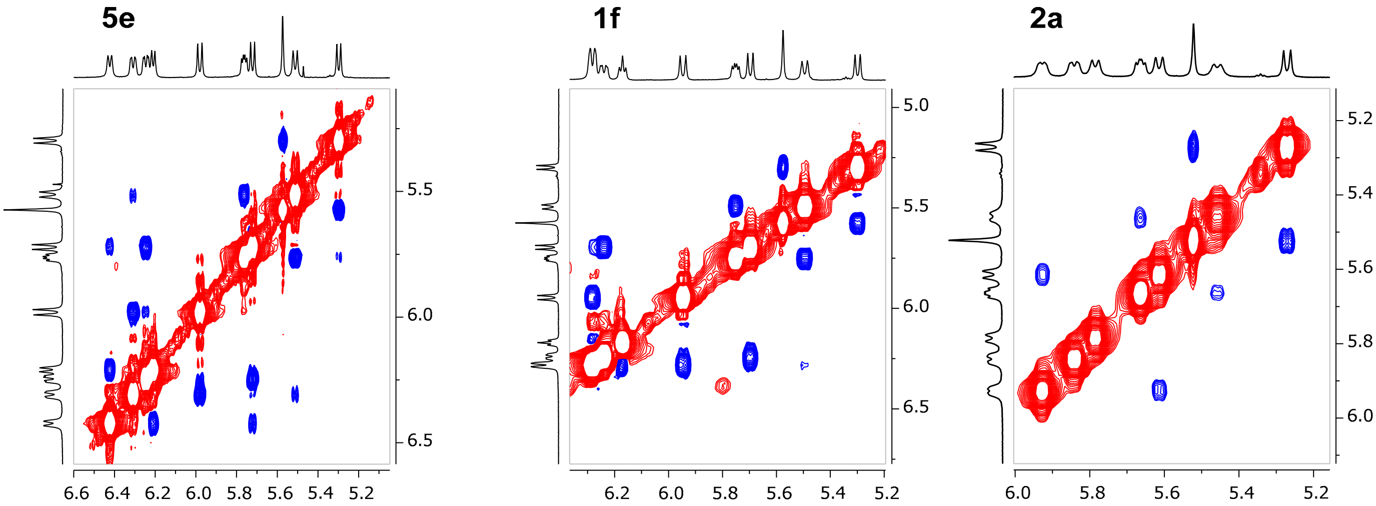

Figure S 48. Part of the NH/NH region of the ROESY spectra, showing NH/N'H cross peaks, characteristic for *trans/trans* conformation of urea groups in compounds **5e, 1f** and **2a**

8. RP-HPLC chromatograms of reaction mixtures of oligourea-esters 5 under alkaline conditions

Analytical RP-HPLC analyses were performed using a Jupiter 4u Proteo 90Å column (4.6 × 250 mm) at a flow rate of 1 mL × min^{-1}. The mobile phase was composed of 0.1% (v/v) TFA/H_2O (phase A) and 0.1% (v/v) TFA− CH_3CN (phase B). The detection was performed at 200 nm and a method 10% of B for 5 min. and a gradient 10-97% of B in 30 min was used.

a) reaction of 5a

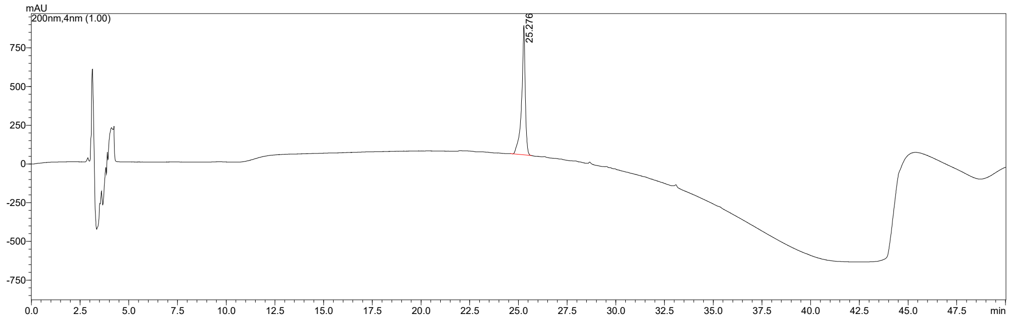

Figure S 49. RP-HPLC chromatogram of crude reaction mixture of **5a** under alkaline conditions. The product **2a** was formed

b) reaction of 5b

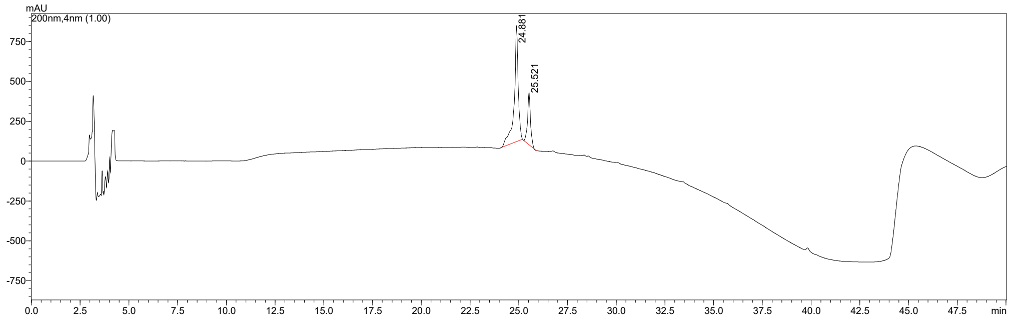

Figure S 50. RP-HPLC chromatogram of crude reaction mixture of **5b** under alkaline conditions. The products **1b**:**2b** were formed in 3:7 ratio.

c) reaction of 5c

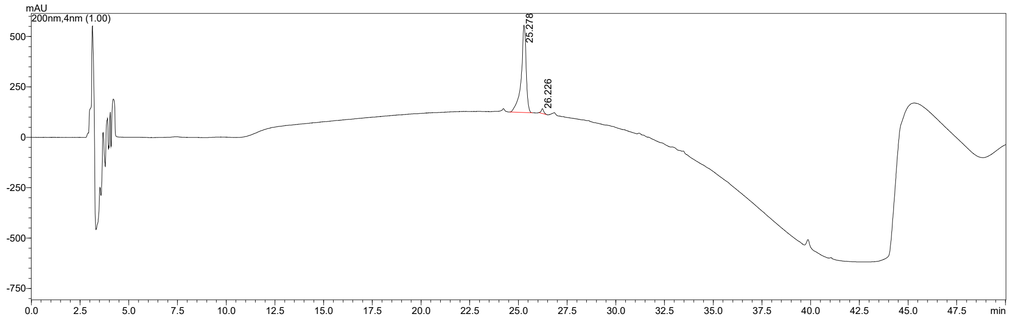

Figure S 51. RP-HPLC chromatogram of crude reaction mixture of **5c** under alkaline conditions. The products **1c**:**2c** were formed in 4:96 ratio.

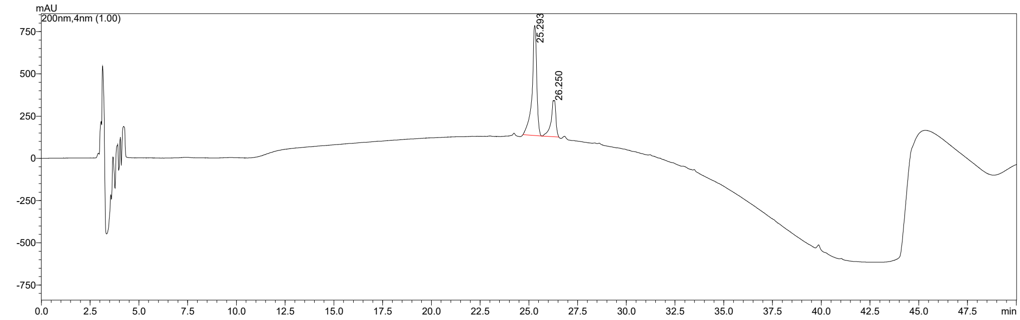

Figure S 52. RP-HPLC chromatogram of crude reaction mixture of **5c** under alkaline conditions after evaporation to dryness. The products **1c**:**2c** were formed in 3:7 ratio.

d) reaction of 5d

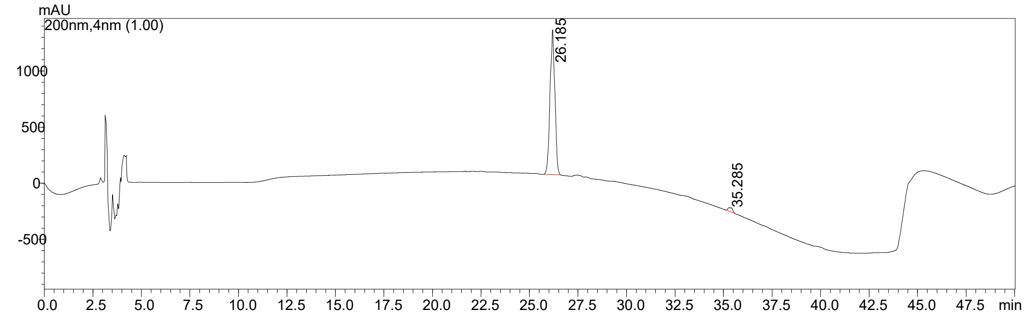

Figure S 53. RP-HPLC chromatogram of crude reaction mixture of **5d** under alkaline conditions. The product **2d** was formed

e) reaction of 5e

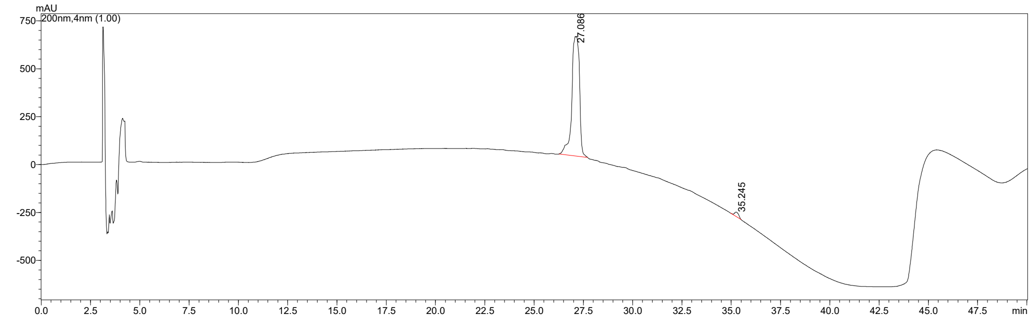

Figure S 54. RP-HPLC chromatogram of crude reaction mixture of **5e** under alkaline conditions. The product **2e** was formed

f) reaction of 5f

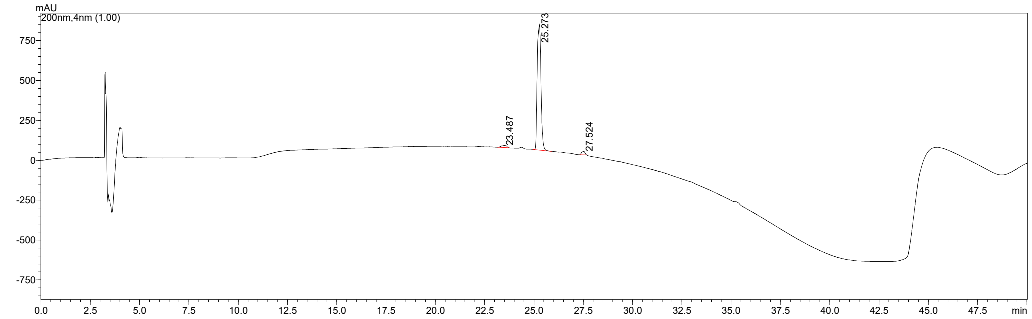

Figure S 55. RP-HPLC chromatogram of crude reaction mixture of **5f** under alkaline conditions. The product **1f** was formed

g) reaction of 5g

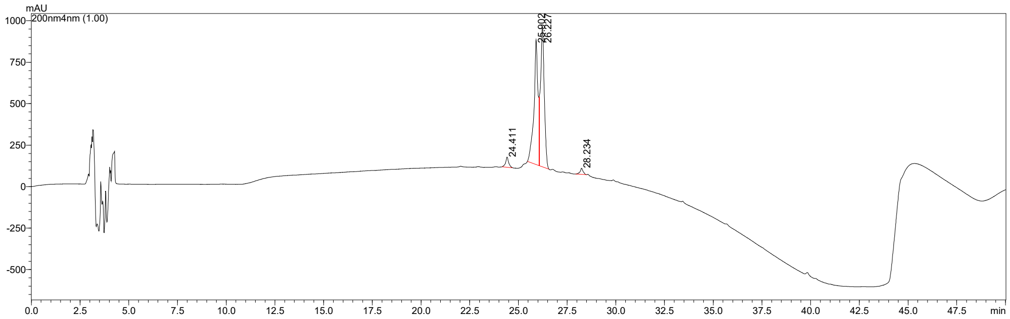

Figure S 56. RP-HPLC chromatogram of crude reaction mixture of **5g** under alkaline conditions. The products **1g**:**2g** were formed in 1:1 ratio.

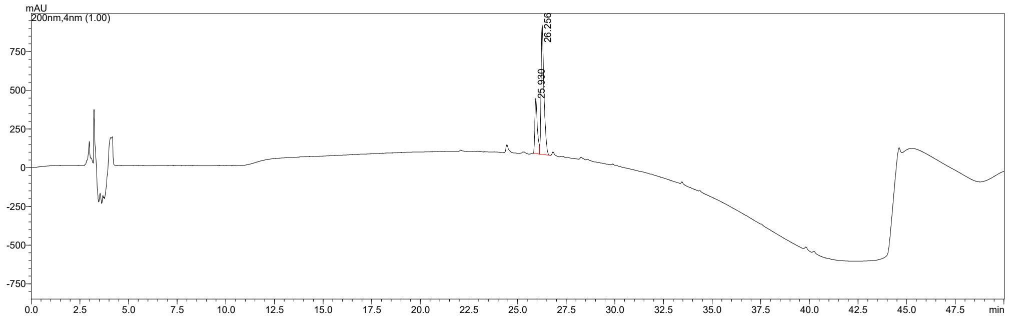

Figure S 57. RP-HPLC chromatogram of crude reaction mixture of **5g** under alkaline conditions after evaporation to dryness. The products **1g**:**2g** were formed in 7:3 ratio.

h) reaction of 5h

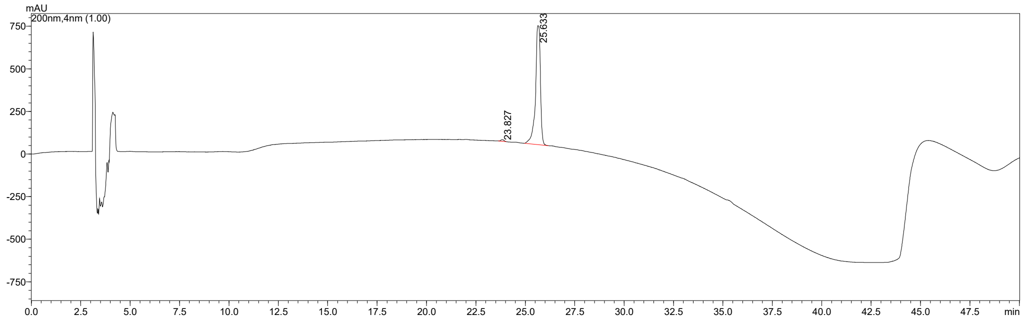

Figure S 58. RP-HPLC chromatogram of crude reaction mixture of **5h** under alkaline conditions. The product **1h** was formed.

i) reaction of 5i

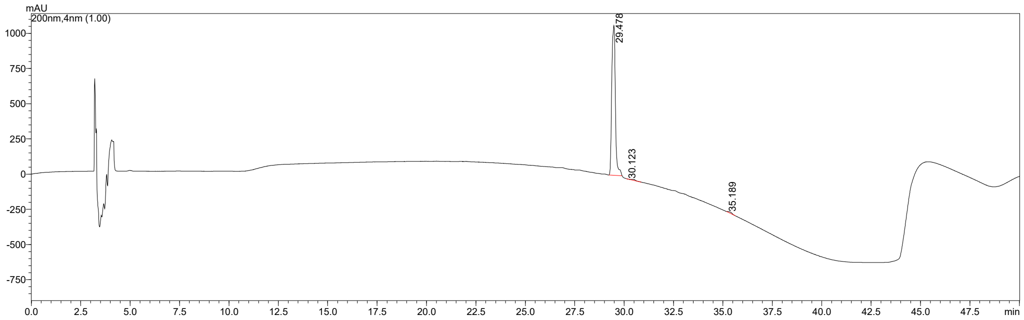

Figure S 59. RP-HPLC chromatogram of crude reaction mixture of **5i** under alkaline conditions. The product **1i** was formed.

j) reaction of 5j

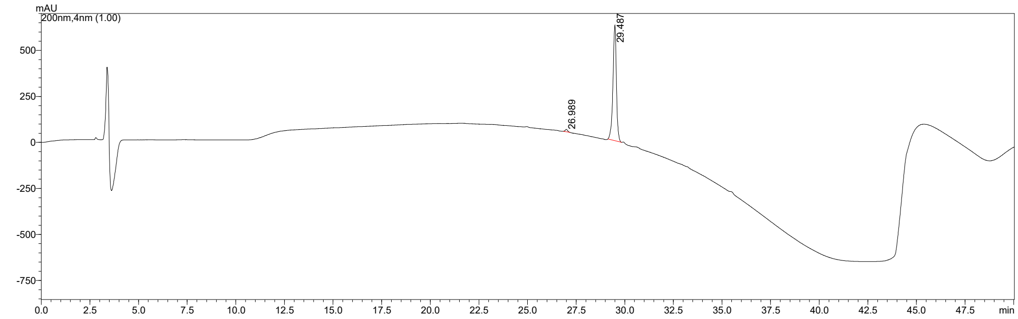

Figure S 60. RP-HPLC chromatogram of crude reaction mixture of **5j** under alkaline conditions. The product **1j** was formed.

9. HRMS of building blocks, oligourea esters and final compounds

a) building blocks BB1-4

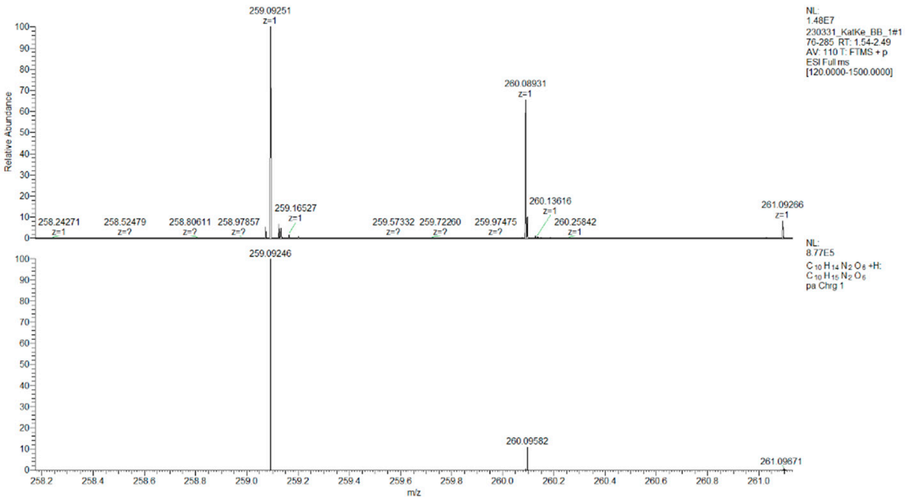

Figure S 61. HRMS spectra of building block **BB1**

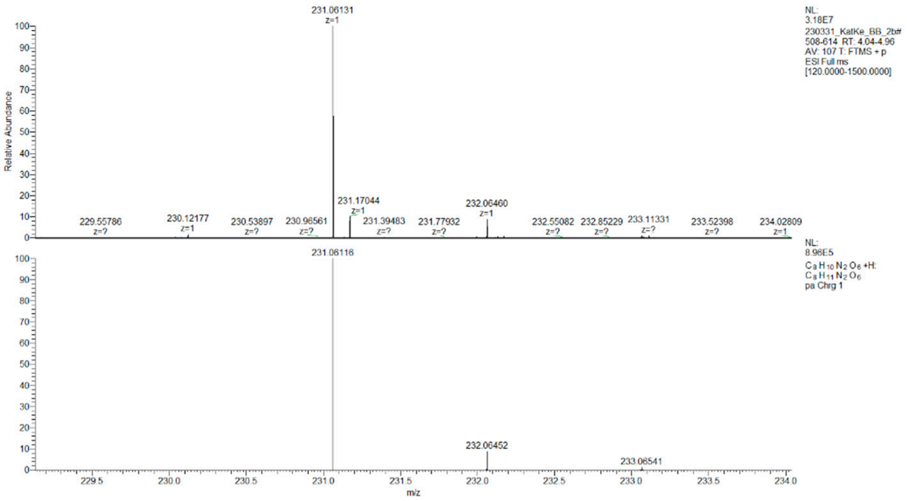

Figure S 62. HRMS spectra of building block **BB2b**

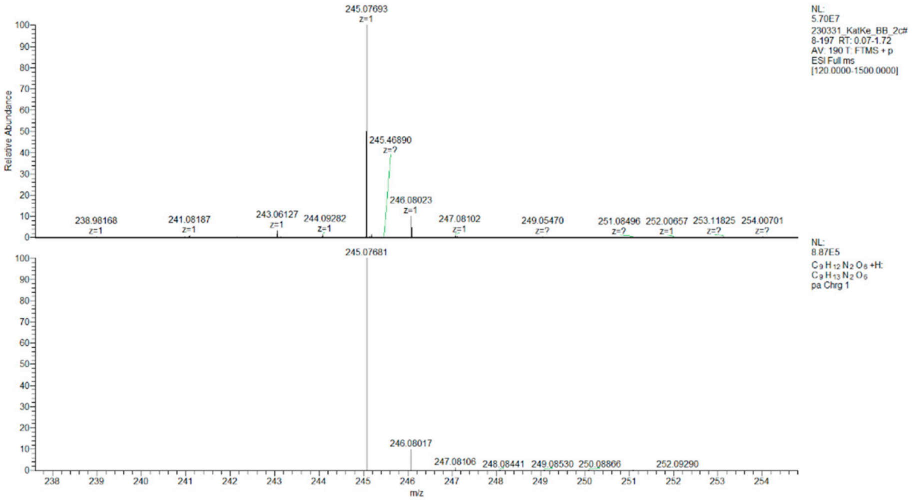

Figure S 63. HRMS spectra of building block **BB2c**

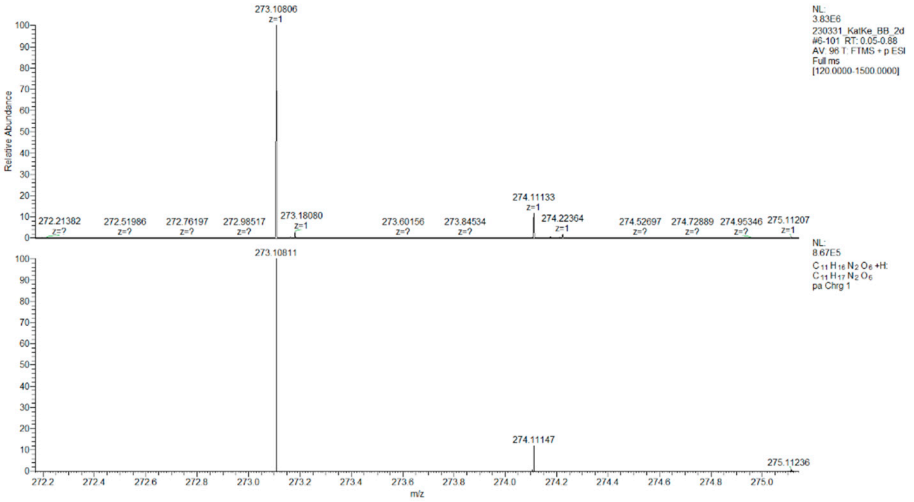

Figure S 64. HRMS spectra of building block **BB2d**

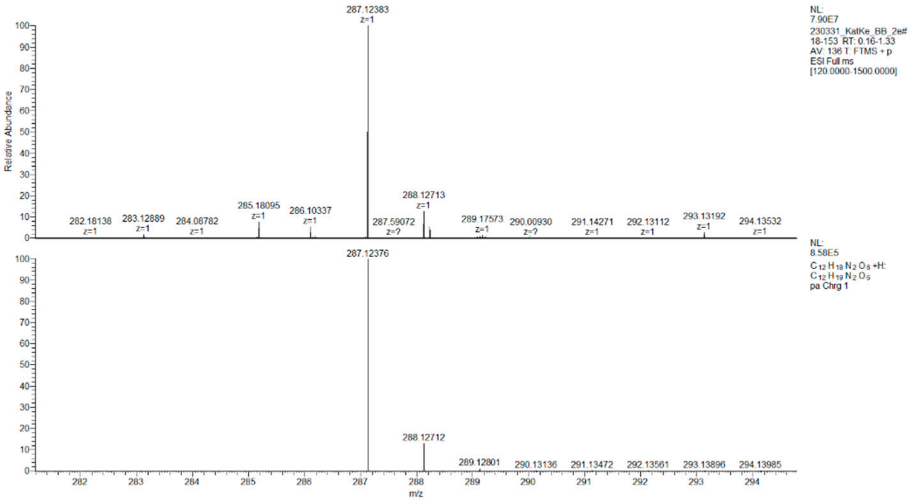

Figure S 65. HRMS spectra of building block **BB2e**

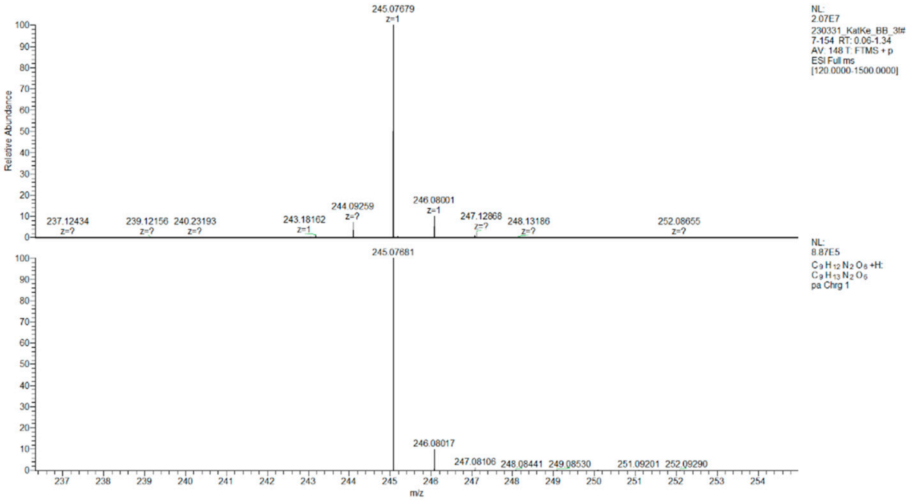

Figure S 66. HRMS spectra of building block **BB3f**

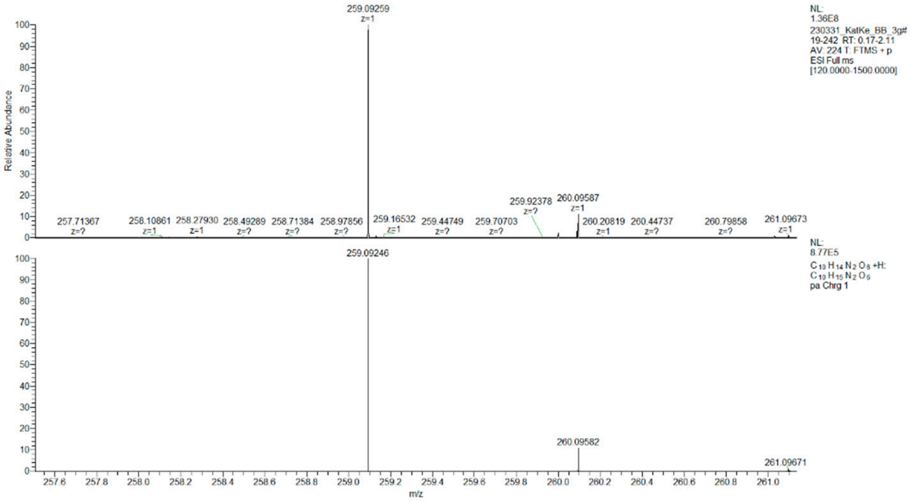

Figure S 67. HRMS spectra of building block **BB3g**

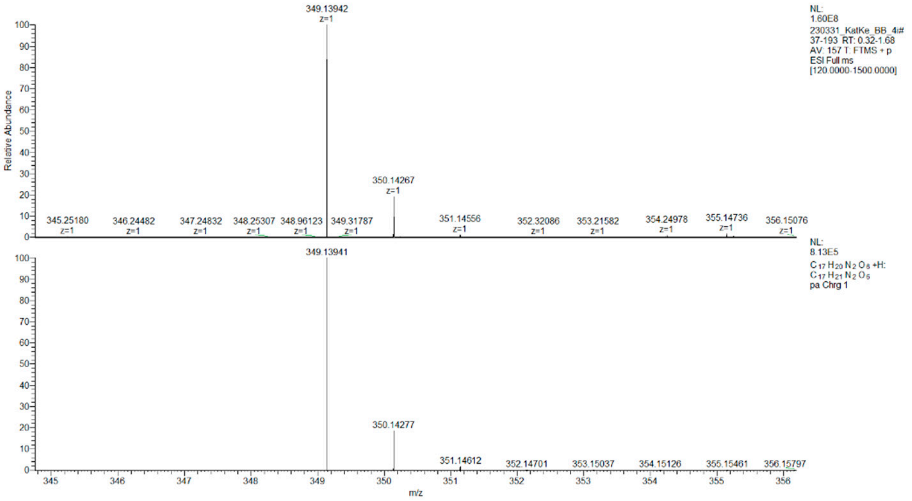

Figure S 68. HRMS spectra of building block **BB4i**

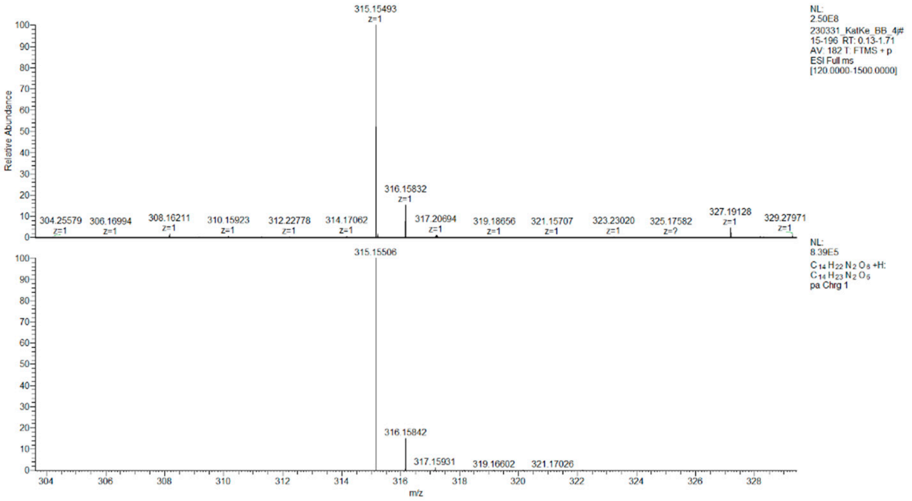

Figure S 69. HRMS spectra of building block **BB4j**

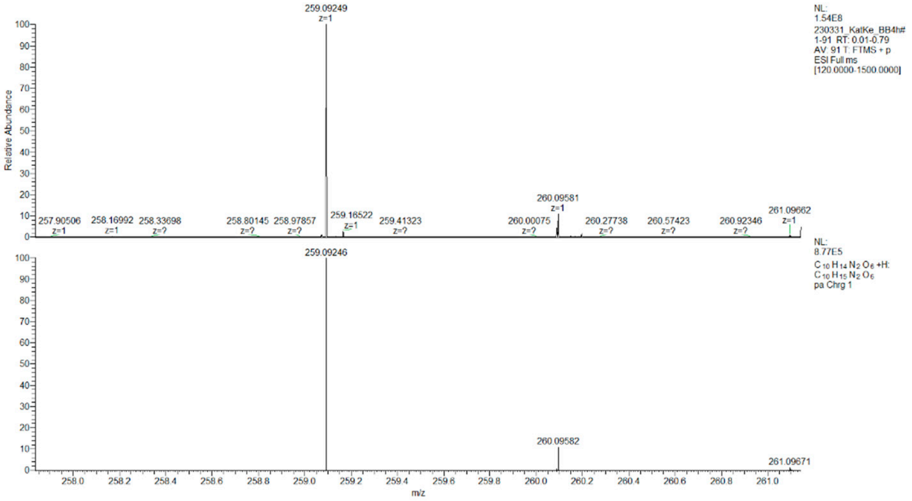

Figure S 70. HRMS spectra of building block **BB4h**

b) oligourea-esters 5

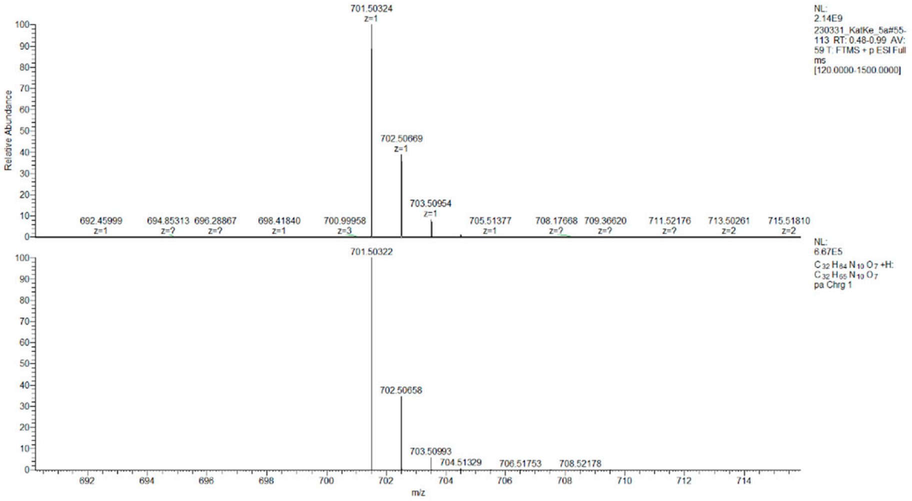

Figure S 71. HRMS spectra of compound **5a**

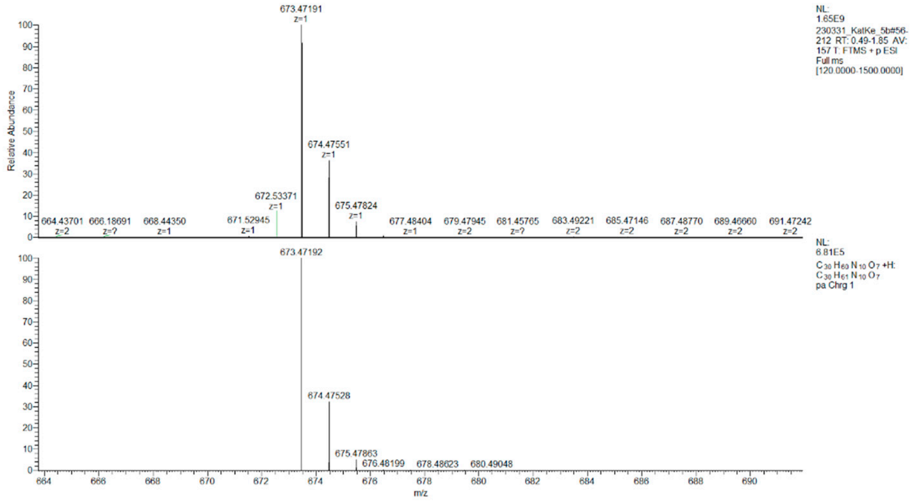

Figure S 72. HRMS spectra of compound **5b**

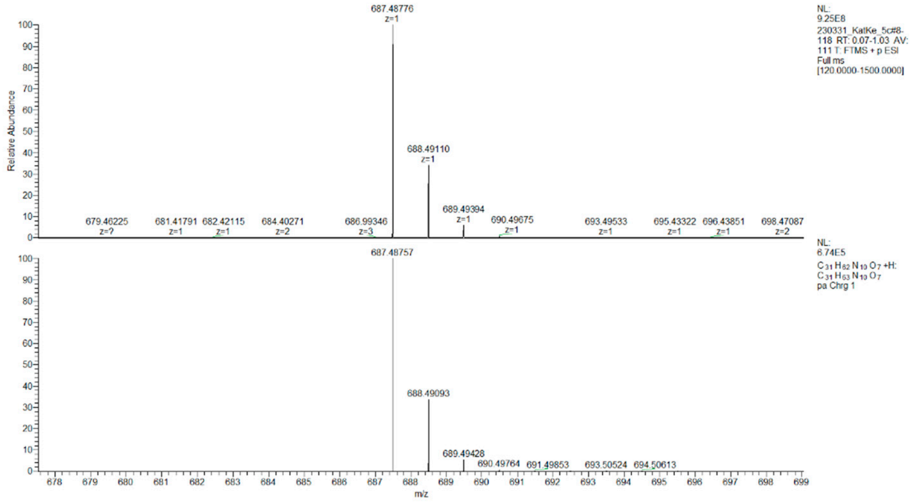

Figure S 73. HRMS spectra of compound **5c**

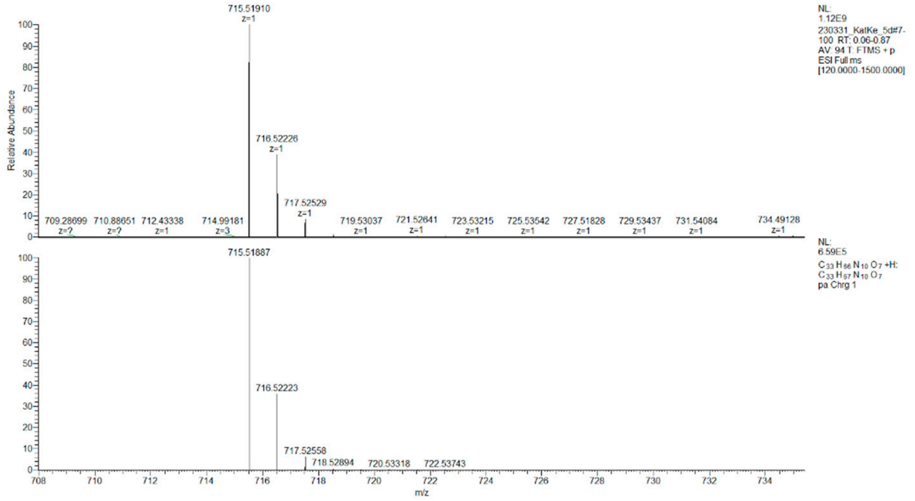

Figure S 74. HRMS spectra of compound **5d**

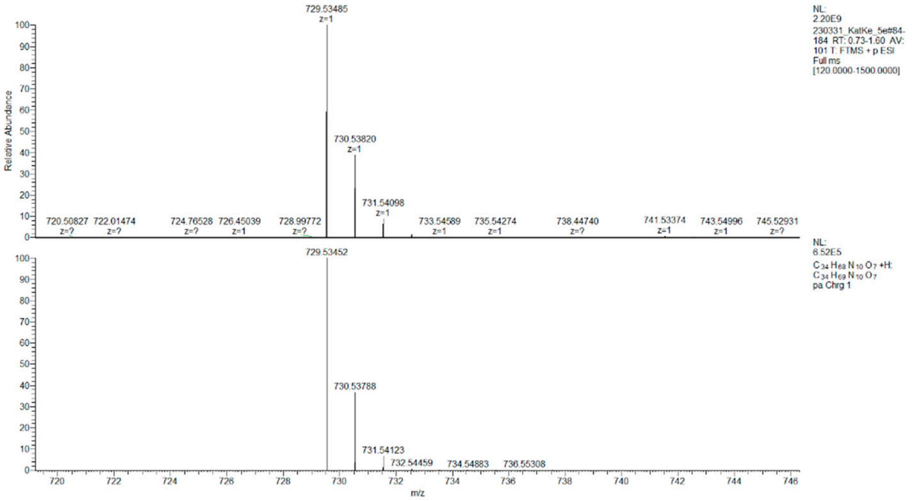

Figure S 75. HRMS spectra of compound **5e**

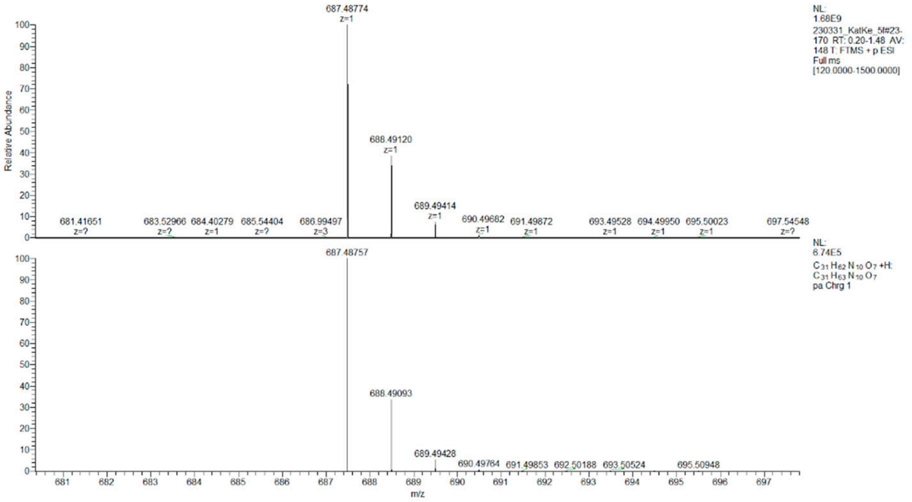

Figure S 76. HRMS spectra of compound **5f**

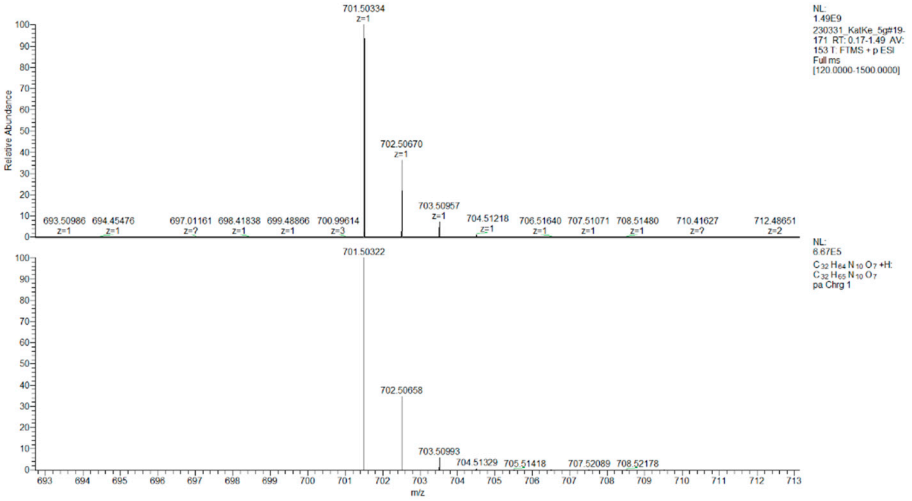

Figure S 77. HRMS spectra of compound **5g**

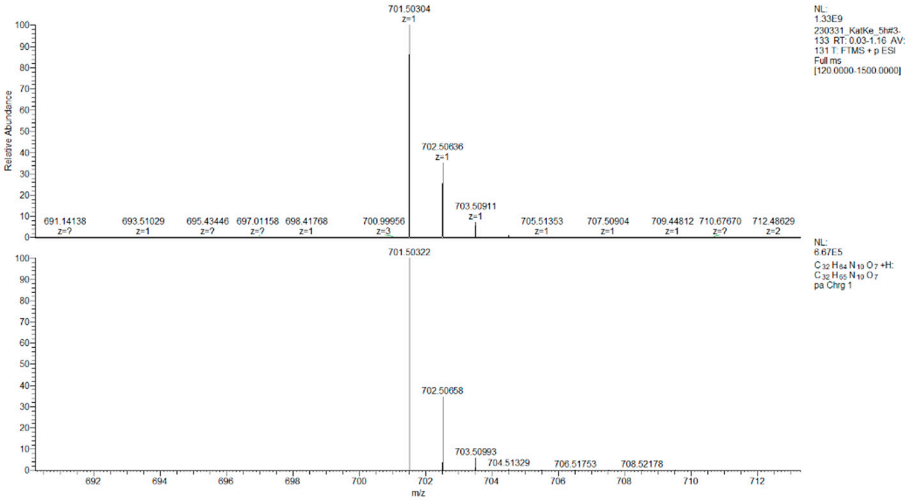

Figure S 78. HRMS spectra of compound **5h**

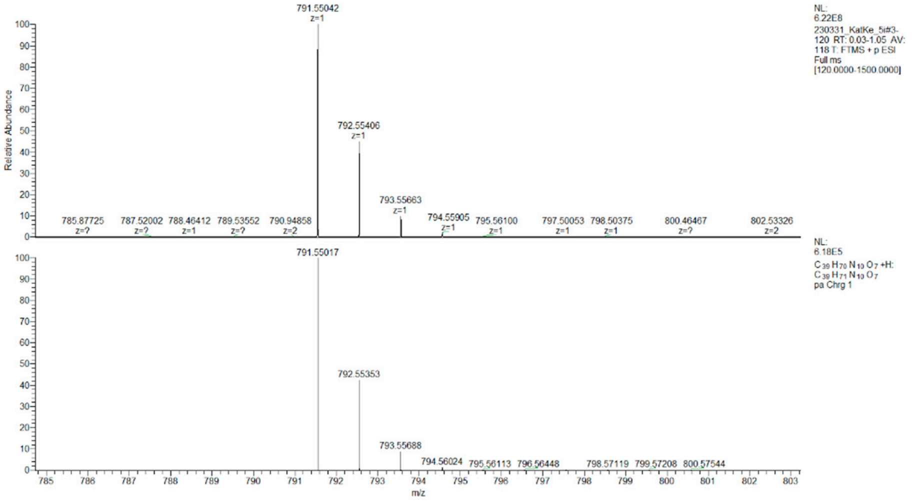

Figure S 79. HRMS spectra of compound **5i**

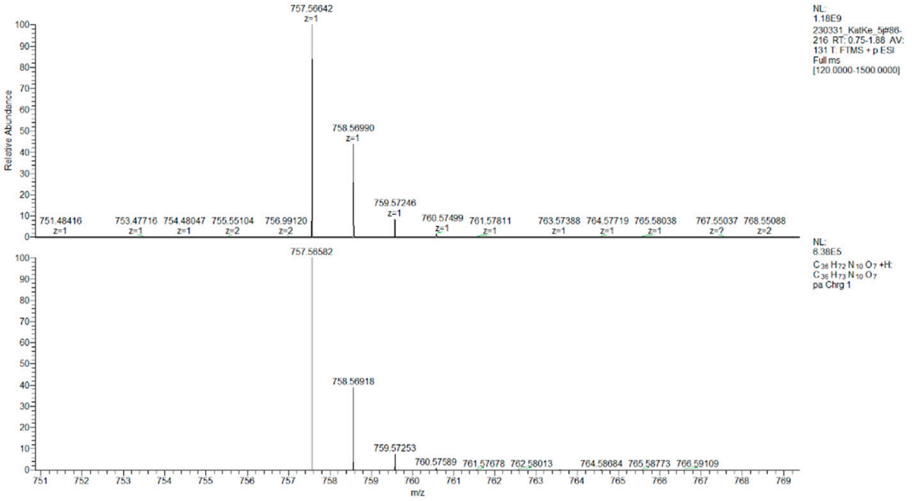

Figure S 80. HRMS spectra of compound **5j**

c) oligourea-acids 1

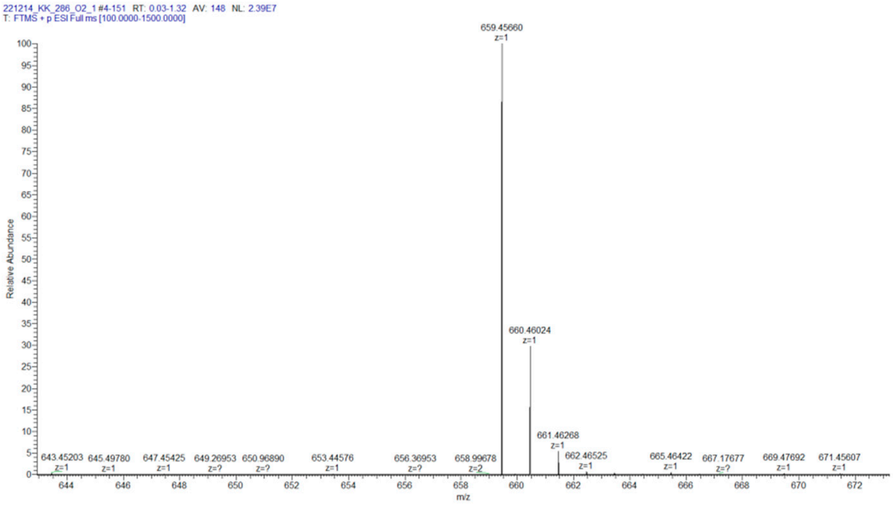

Figure S 81. HRMS spectra of compound **1b**.

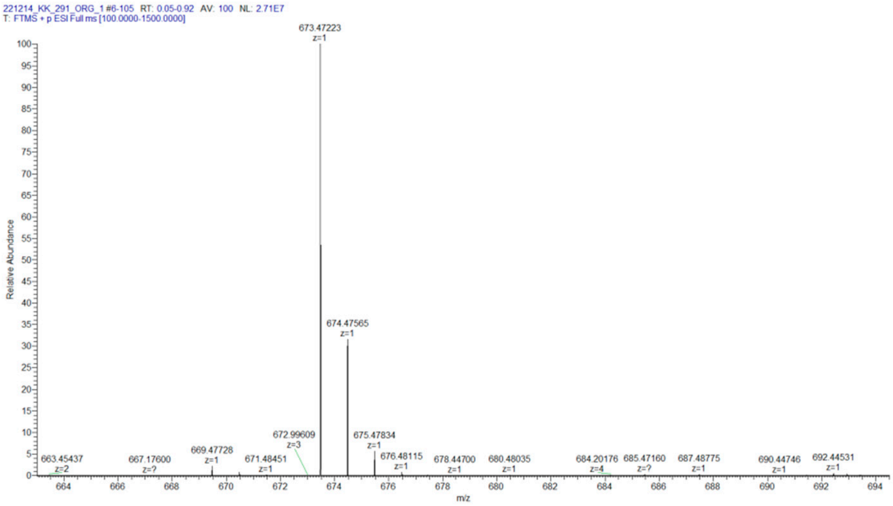

Figure S 82. HRMS spectra of compound **1c**

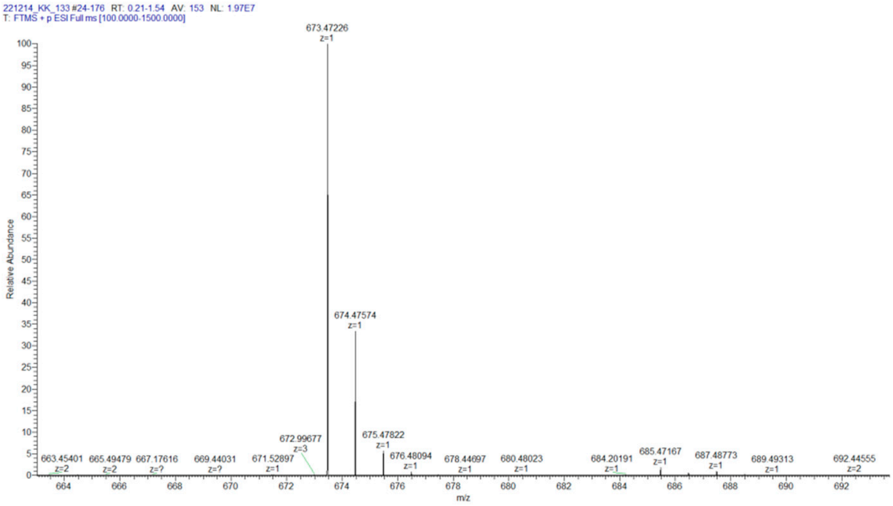

Figure S 83. HRMS spectra of compound **1f**

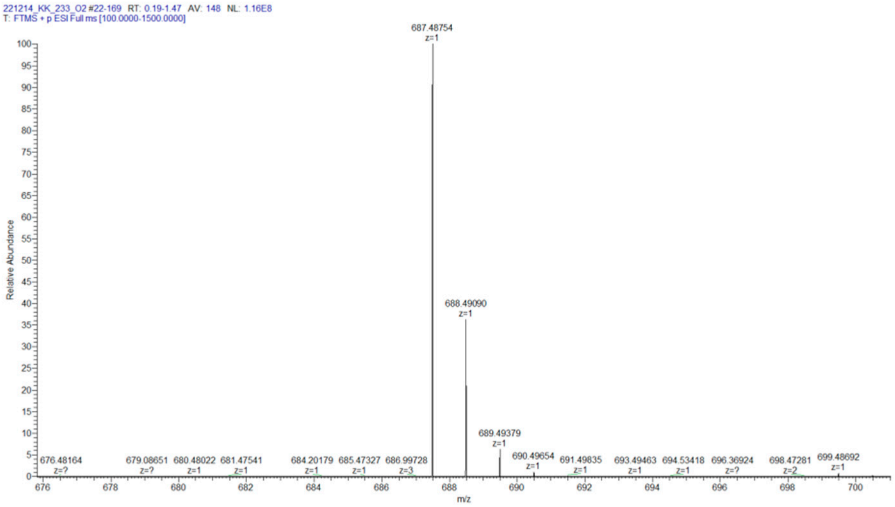

Figure S 84. HRMS spectra of compound **1g**

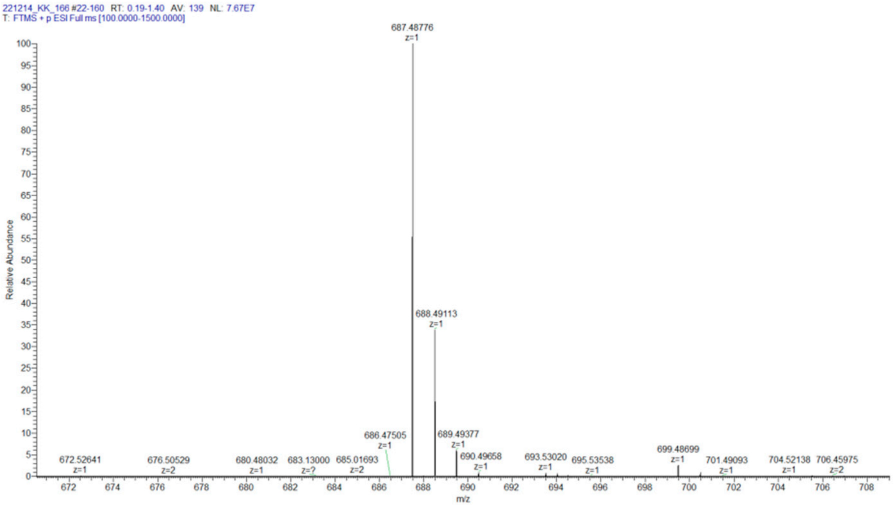

Figure S 85. HRMS spectra of compound **1h**

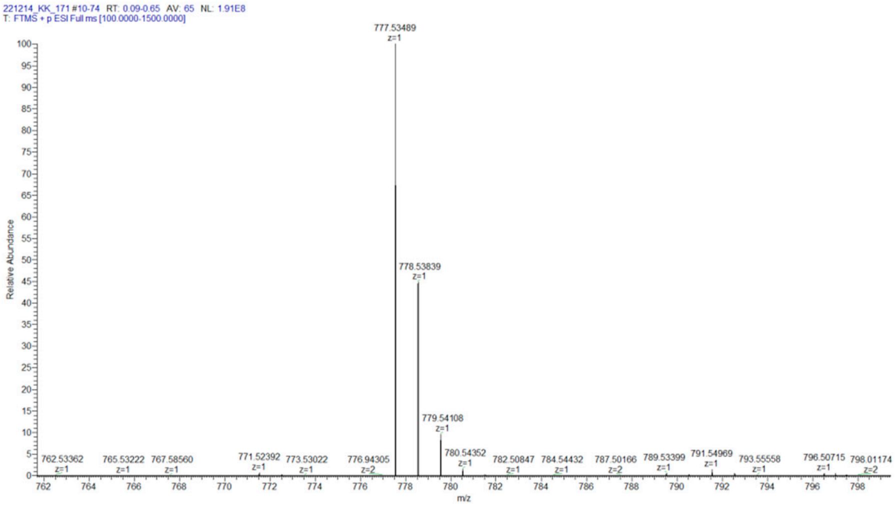

Figure S 86. HRMS spectra of compound **1i**

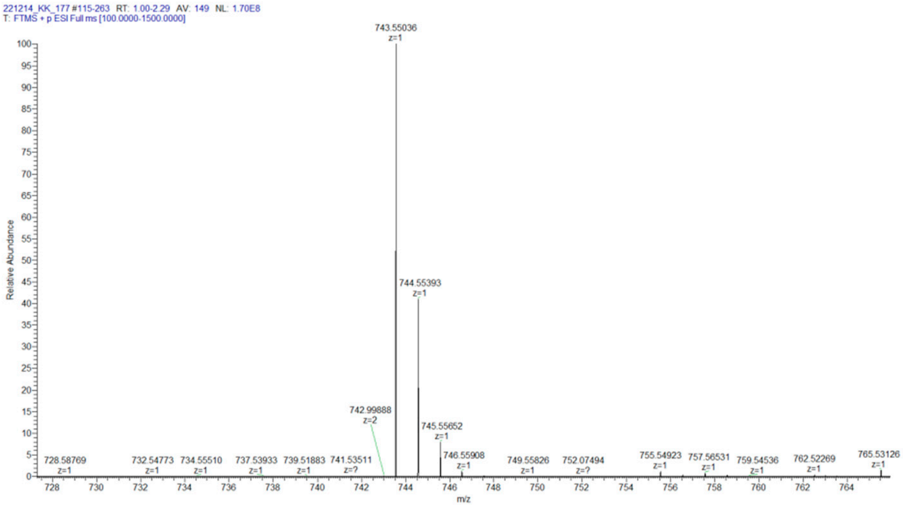

Figure S 87. HRMS spectra of compound **1j**

d) oligourea-hydantoin/dihydrouracil 2

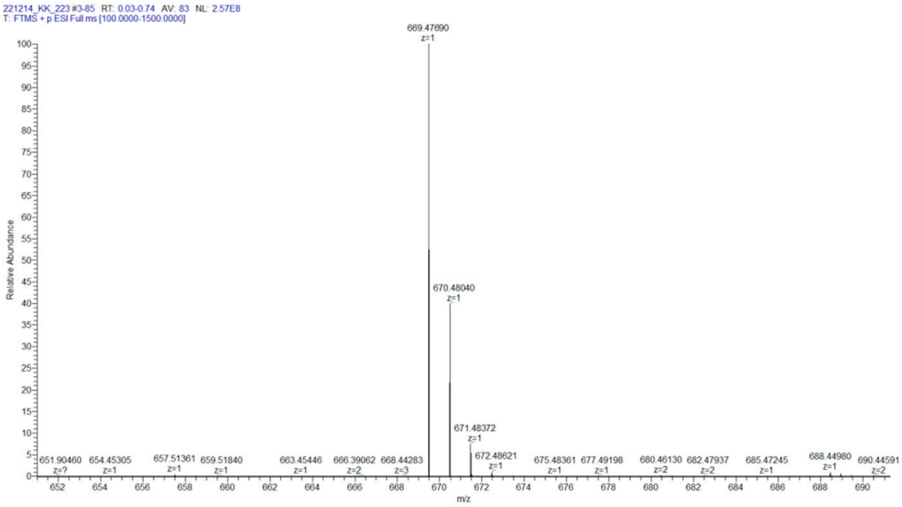

Figure S 88. HRMS spectra of compound **2a**

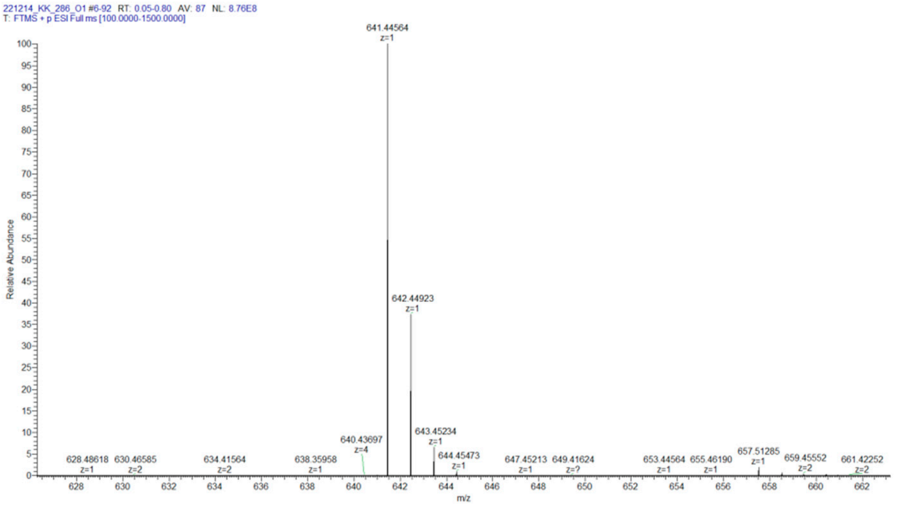

Figure S 89. HRMS spectra of compound **2b**

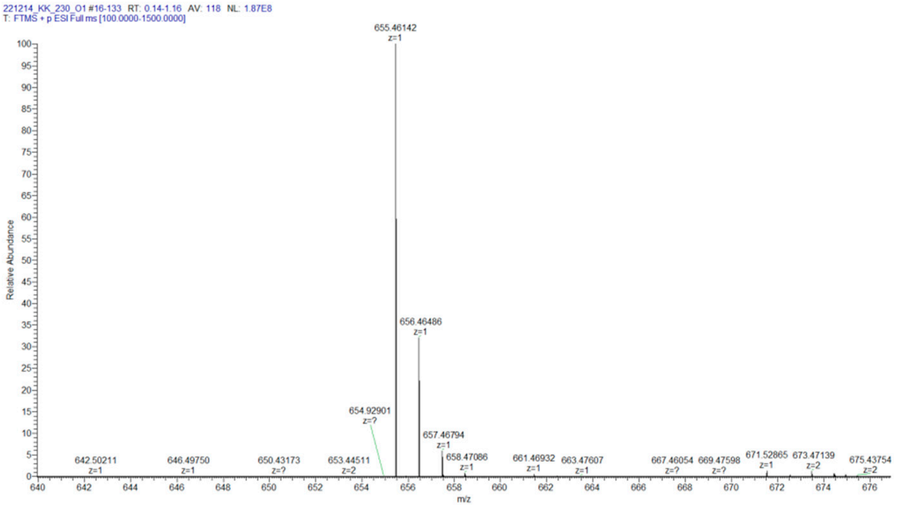

Figure S 90. HRMS spectra of compound **2c**

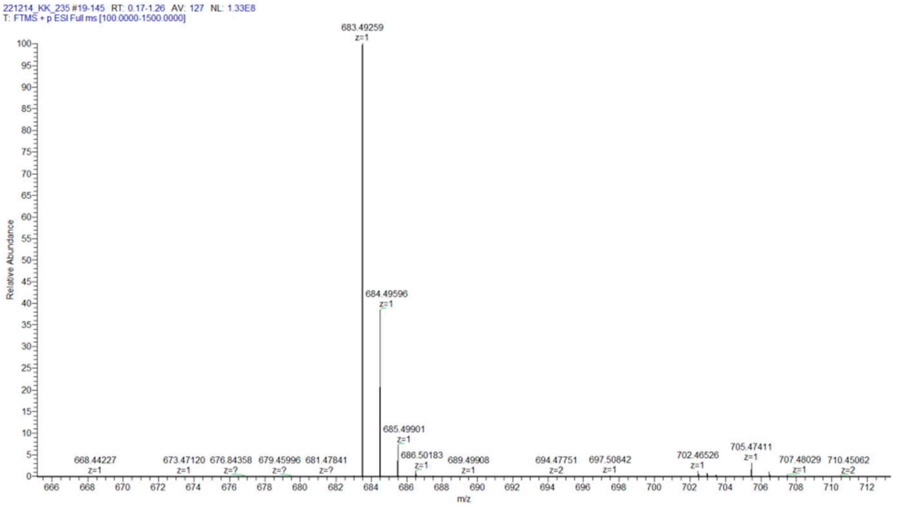

Figure S 91. HRMS spectra of compound **2d**

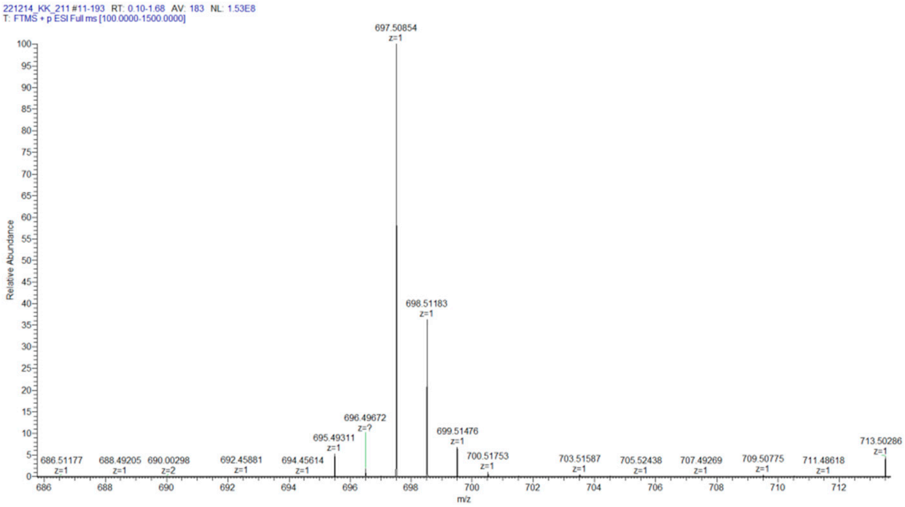

Figure S 92. HRMS spectra of compound **2e**

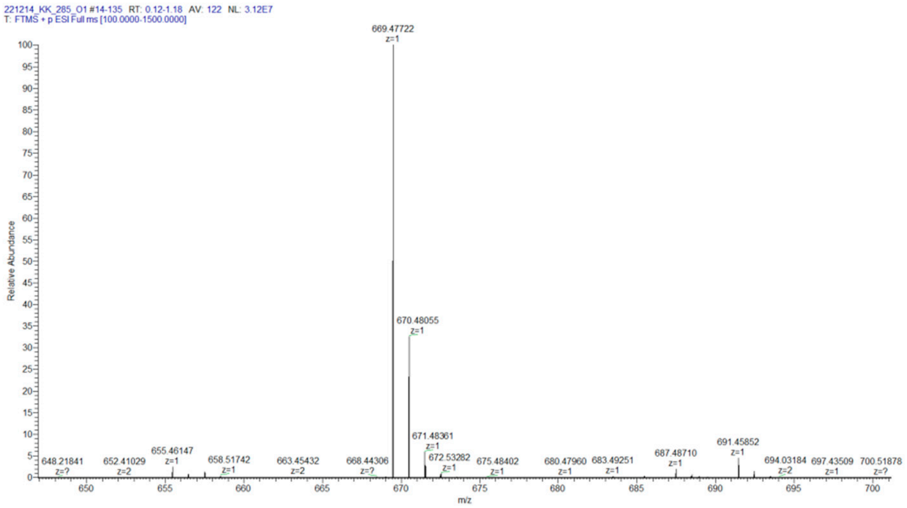

Figure S 93. HRMS spectra of compound **2g**

10. CD spectra of selected oligourea-esters 5, oligourea-acids 1 and oligourea-hydantoin/dihydrouracil 2

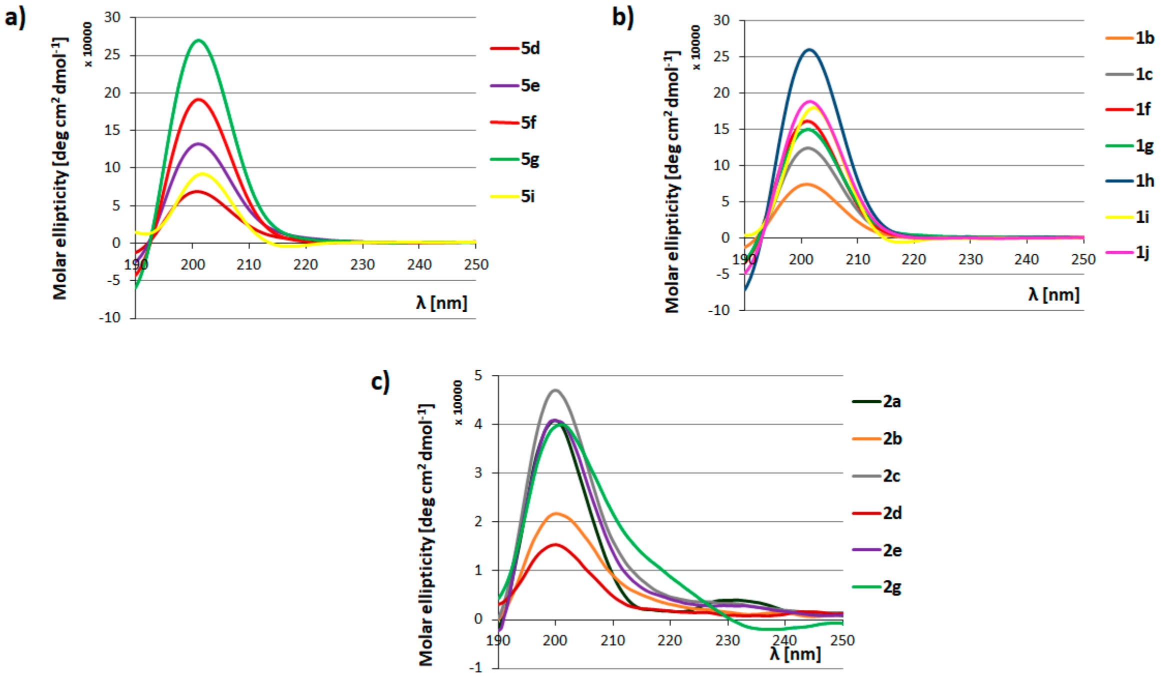

Figure S 94. ECD spectra measured at c= 0.2 mM in TFE, at 20°C: a) oligourea-esters; b) oligourea-acids; c) oligourea-hydantoins/dihydrouracil

11. Additional HPLC chromatograms

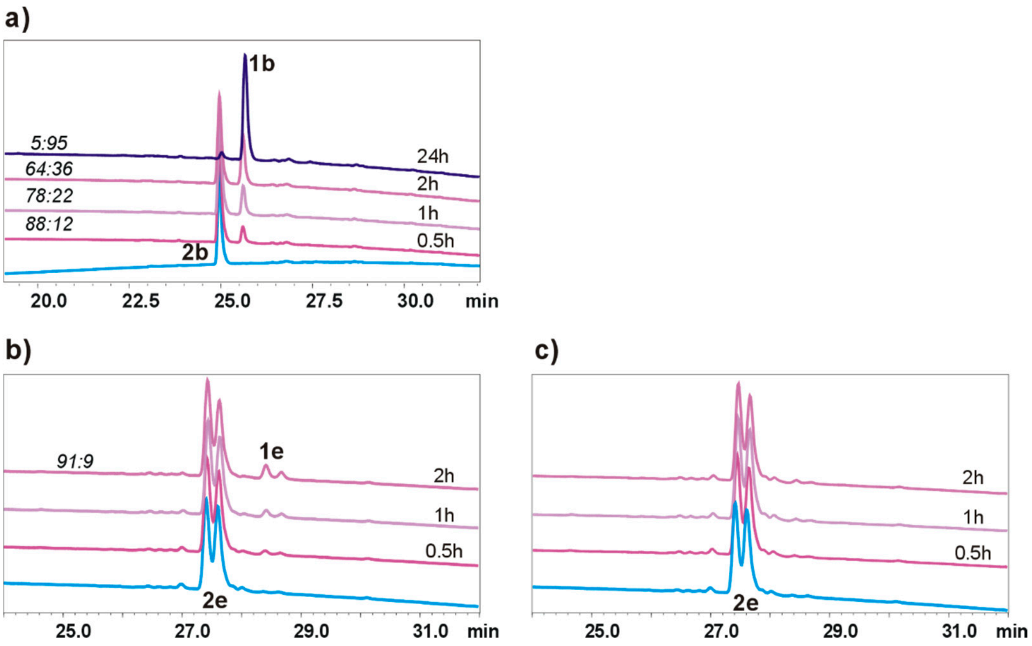

Figure S 95. Stability studies of oligourea-hydantoin under alkaline conditions: a) stability studies of compound **2b** at RT; b) stability studies of compound **2e** at 45°C; c) stability studies of compound **2e** at RT

12. Crystallography

The X-ray measurement of **2a** was performed at 130.0(5) K on a Bruker D8 Venture PhotonII diffractometer equipped with a INCOATEC IμS micro-focus source (CuKα, λ = 1.54178 Å) and a mirror monochromator. A total of 3586 frames were collected with Bruker APEX3 program.[1] The frames were integrated with the Bruker SAINT software package[2] using a narrow-frame algorithm. The integration of the data using a monoclinic unit cell yielded a total of 71438 reflections to a maximum ϑ angle of 59.09° (0.90 Å resolution), of which 22715 were independent (average redundancy 3.145, completeness = 99.4%, R_{int} = 4.85%, R_{sig} = 5.04%) and 16483 (72.56%) were greater than $2\sigma(F^2)$. The final cell constants of a = 14.9921(4) Å, b = 35.4478(9) Å, c = 15.0011(4) Å, β = 90.7754(18)°, V = 7971.4(4) Å^3, are based upon the refinement of the XYZ-centroids of 9904 reflections above 20 $\sigma(I)$ with 5.895° < 2ϑ < 117.8°. Data were corrected for absorption effects using the Multi-Scan method (SADABS).[3] The ratio of minimum to maximum apparent transmission was 0.863. The calculated minimum and maximum transmission coefficients (based on crystal size) are 0.784 and 0.964.

The structure was solved and refined using SHELXTL Software Package[4,5] using the space group $P2_1$, with Z = 1 for the formula unit, $C_{248}H_{474}N_{80}O_{53}$ and Flack parameter[6] yielding 0.01(8). The final anisotropic full-matrix least-squares refinement on F^2 with 1802 variables converged at $R1$ = 7.78%, for the observed data and $wR2$ = 23.16% for all data. The goodness-of-fit was 1.036. The largest peak in the final difference electron density synthesis was 0.460 e^-/Å^3 and the largest hole was -0.317 e^-/Å^3 with an RMS deviation of 0.060 e^-/Å^3. On the basis of the final model, the calculated density was 1.130 g/cm^3 and F(000), 2946 e^-. Crystal data and refinement parameters for **2a** are collected in **Table S 3**. The crystal contains in the asymmetric part four oligourea molecules and 2½ water moieties (hydrogen atoms not assigned resulting in three Alerts B in the checkCIF report). The scattering power of the crystal is limited giving another Alert B. In one of the oligourea the hydantoin part is disordered over two positions with refined occupancy ratio yielding 0.69(2):0.31(2) what is associated with two alternative positions of one of the water molecules and occupancy ratio equal 0.31(2):0.69(3). The crystal is probably composed of two domains rotated along Y axis. This is probably related to pseudotetragonal symmetry of the lattice. The structure was refined with TWIN instruction 0 0 -1 0 1 0 1 0 0 (4-fourfold rotation along Y) giving BASF parameter equal 0.062(2). Introduction of the TWIN refinement slightly improved final R parameters.

During structure refinement a number of restraints (distances, angles, ADPs) was used. All major component (>0.5) disordered moieties were refined anisotropically. All hydrogen atoms were placed in calculated positions and refined within the riding model. The temperature factors of hydrogen atoms were not refined and were set to be either 1.2 or 1.5 times larger than U_{eq} of the corresponding heavy atom. The atomic scattering factors were taken from the International Tables.[7] Molecular graphics was prepared using program Mercury 2022.3.0.[8] Atomic displacement parameters of **2a** are presented in **Figure S 96**. Packing of the molecules are presented in **Figure S 97**. The structure in the form of ribbon representation generated in CCP4mg package[9] is presented in **Figure S 98**.

Table S 3. Data collection and structure refinement parameters for **2a**

**Formula**	$C_{248}H_{474}N_{80}O_{53}$
M_x/ g·mol^{-1}	5425.04
T/ K	130.0(5)
λ/ Å	1.54178
**Crystal size**	0.056 × 0.172 × 0.389
**Space group**	$P2_1$
**Unit cell dimensions**	a = 14.9921(4) Å<br>b = 35.4478(9) Å β = 90.7754(18)°<br>c = 15.0011(4) Å
V/ Å^3, Z	7971.4(4), 1
D_x/ g·cm^{-3}	1.130
μ/ mm^{-1}	0.659
F(000)	2946
ϑ_{min}, ϑ_{max}	2.49°, 59.09°
**Index ranges**	$-15 \leq h \leq 16$<br>$-39 \leq k \leq 39$<br>$-16 \leq l \leq 16$
**Reflections collected/ independent**	71438 / 22715<br>R_{int} = 0.0485
**Completeness**	99.4%
**Absorption correction**	Multi-Scan
T_{max}, T_{min}	0.964, 0.784
**Refinement method**	Full-matrix LSQ on F^2
**Data / restraints / parameters**	22715 / 118 / 1802
**Goodness-of-fit on F^2**	1.036
**Final R indices**	16483 data; $I>2\sigma(I)$<br>$R1$ = 0.0778, $wR2$ = 0.2094

	all data<br>$R1$ = 0.1005, $wR2$ = 0.2316
**Absolute structure parameter**	0.01(8)
**$\Delta\rho_{max}$, $\Delta\rho_{min}$**	0.460, -0.317 eÅ^{-3}

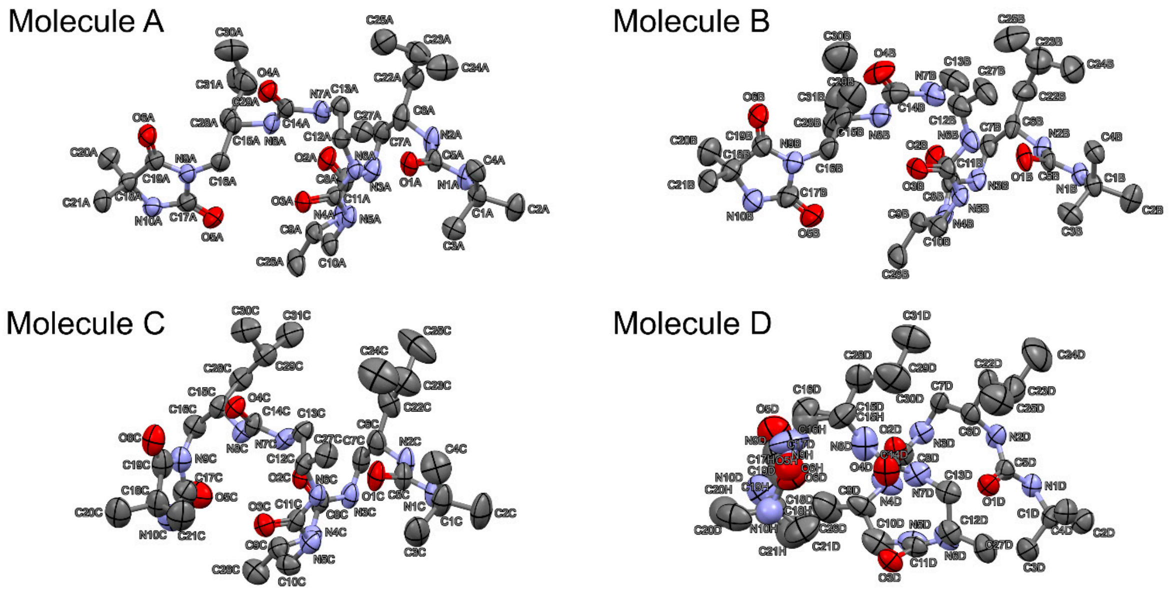

Figure S 96. Atomic displacement parameters of **2a** at 50% probability level presented for each molecule separately. Solvent moieties and hydrogen atoms omitted for clarity.

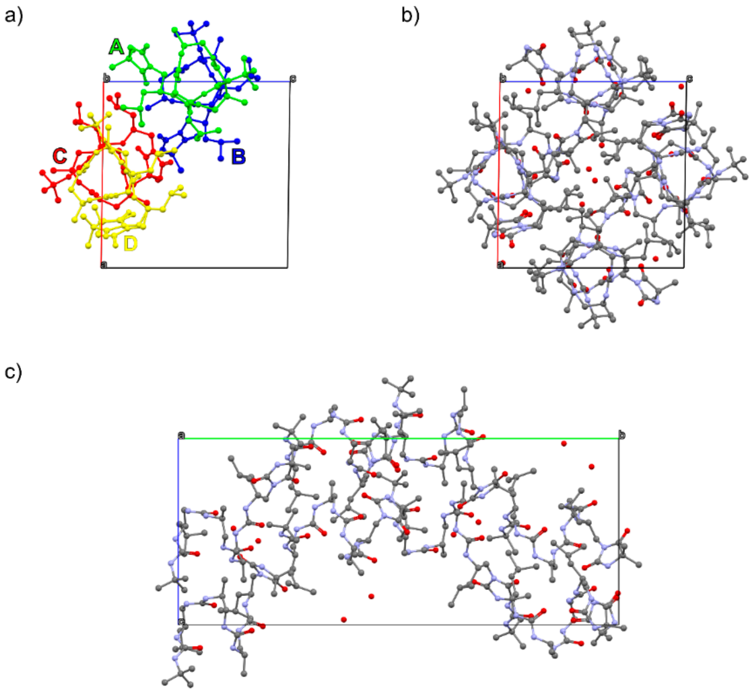

Figure S 97. Asymmetric part of the structure with differently coloured molecules a) and packing diagrams of **2a** - view along [010] b) and [100] c). H atoms omitted for clarity.

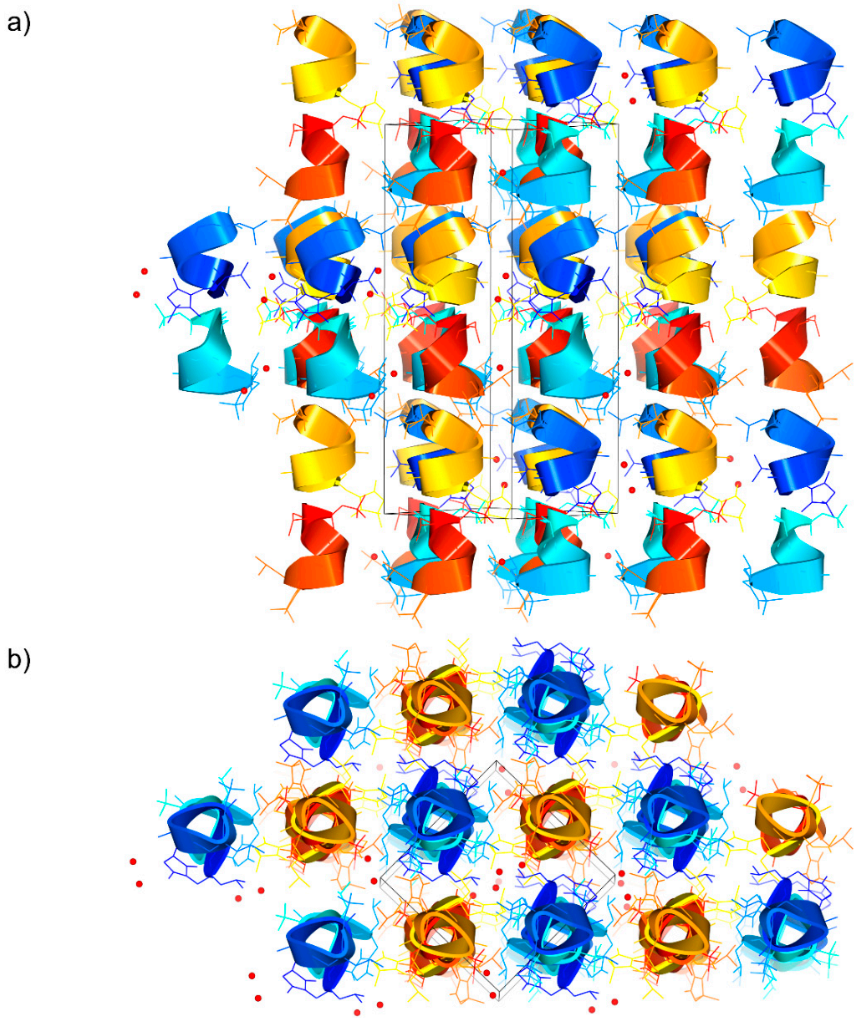

Figure S 98. Structure view using ribbon representation for each peptide molecule, view parallel to [010] a), view along [010] b).

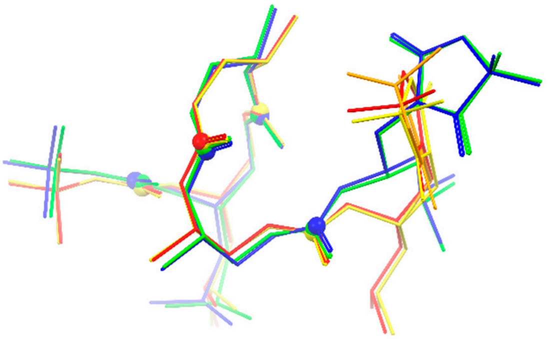

Figure S 99. Overlay of molecules A-D; matching for all urea carbons (indicated as balls)

Table S 4. The backbone torsion angles in **2a** (taken from the crystal structure)

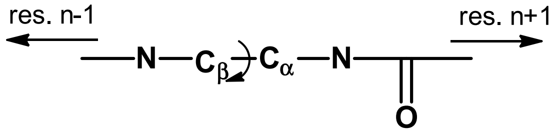

**Residue**	**A**	**B**	**C**	**D**
**Leu1u**	50(1)	54(1)	54(1)	54(1)
**Ala2u**	52.8(9)	57.4(9)	54(1)	55(1)
**Ala3u**	53.8(9)	47(1)	52.5(9)	55(1)
**Leu4(hyd)**	-159.3(7)	-163.6(8)	41(1)	43(2)

13. References

1. APEX3 V2019, Bruker Nano, Inc., **2019**.
2. SAINT V8.40A, Bruker Nano, Inc., **2019**.
3. SADABS V2016/2, Bruker Nano, Inc., **2019**.
4. *G. M. Sheldrick*, *Acta Cryst.*, **2015**, *A71*, 3-8.
5. G. M. Sheldrick, *Acta Cryst*., **2015**, *C71*, 3–8.
6. S. Parsons, H. D. Flack, T. Wagner, *Acta Cryst.*, **2013**, *B69*, 249–259.
7. *International Tables for Crystallography*, Ed. A. J. C. Wilson, Kluwer: Dordrecht, **1992**, Vol.C.
8. C. F. Macrae, I. Sovago, S. J. Cottrell, P. T. A. Galek, P. McCabe, E. Pidcock, M. Platings, G. P. Shields, J. S. Stevens, M. Towler and P. A. Wood, "Mercury 4.0: from visualization to analysis, design and prediction", *J. Appl. Cryst.*, **2020**, *53*, 226-235.
9. S. McNicholas, E. Potterton, K. S. Wilson, M. E. M. Noble, Presenting your structures: the CCP4mg molecular-graphics software, *Acta Cryst.*, **2011**, *D67*, 386-394.
